# Skill Session on Writing Patient Assessments for Pediatric Clerkship Students

**DOI:** 10.15766/mep_2374-8265.11029

**Published:** 2020-11-09

**Authors:** Sofia Khera, Sheela Gavvala, Raymond Parlar-Chun, Hanna Huh, Jean Hsu, Christine Ford

**Affiliations:** 1 Assistant Professor, Department of Pediatric Hospital Medicine, McGovern Medical School at the University of Texas Health Science Center at Houston; 2 Education Specialist, Office of Educational Programs, McGovern Medical School at the University of Texas Health Science Center at Houston

**Keywords:** Clinical Skills, Clinical Reasoning, Clerkship Curriculum, Patient Assessment, Diagnostic Reasoning, Pediatrics, Clinical/Procedural Skills Training, Editor's Choice

## Abstract

**Introduction:**

Formulating written patient assessments requires the student to synthesize subjective and objective information and use clinical reasoning to reach a diagnosis. Medical students lack this skill, and clinical experience is not enough to acquire it. This session provides a structured process for learning how to formulate a patient assessment.

**Methods:**

Third-year medical students participated in a large-group interactive skill session at the beginning of their pediatrics clerkship. Instructors following a scripted model walked students through practice examples to ultimately formulate a complete written patient assessment. The session covered data synthesis, use of appropriate medical terminology, and differential diagnosis development. Students used an audience response system to practice these skills.

**Results:**

Over 1 academic year, 250 medical students participated in six sessions, with an average of 40–50 attendees per session. Over 90% of students completed pre- and postsession written patient assessments. These assessments were evaluated using portions of the Pediatric History and Physical Exam Evaluation grading rubric. The session had a positive effect on patient assessment formulation skills, with a significant increase in scores after the session.

**Discussion:**

The session improved students' skill in generating more complete written patient assessments. Almost all students found the session valuable regardless of prior clinical experience. Nearly 50% of students felt inadequately prepared to formulate a written patient assessment prior to the session, revealing a skills gap for learners and a curricular teaching gap. This skill session provided a structured method and active learning format for teaching this essential clinical skill.

## Educational Objectives

By the end of this activity, learners will be able to:

1. Identify essential components of a written patient assessment.
2. Synthesize and interpret subjective and objective patient data using appropriate medical terminology and semantic qualifiers.
3. Formulate a complete patient assessment statement that also demonstrates clinical reasoning to reach a diagnosis and its differential.

## Introduction

Formulating written patient assessment statements that are precise and accurate is an essential clinical skill for medical students, encompassing synthesis, knowledge application, and clinical reasoning.^1^ Whereas the history, physical, and diagnostic data can be obtained directly from the patient or the patient's chart, the written patient assessment statement requires critical thinking to communicate the key aspects of the case that warrant inclusion, in addition to the application of medical terminology that is a summative reflection of the entire case.^2–4^

Emphasis on obtaining thorough histories is provided through curricula involving standardized patients and standardized testing such as clinical skills examinations^5^; however, there is a lack of structured curricula specifically on the formulation of written patient assessments.^4^ Most students learn by observation or by trial and error with inconsistent feedback.^1^ Many institutions have noted via needs assessments that the formulation of a written patient assessment is a clinical communication and documentation skill deficiency.^6^ In reviewing the literature, internal medicine and pediatrics are the primary clerkships where general patient documentation and presentation are identified as a skill deficiency.^7,8^ For example, the postgraduate program of the Department of Hospital Medicine at Ochsner Clinic in Louisiana conducts a note-writing workshop for students and residents in the first week of their internal medicine rotation.^9^ The Pediatric Boot Camp also includes, in its rigorous 1-day course, aspects of formulating assessment and plans among other clinical communication skills, such as how to answer pages and manage organization, but this widely encompassing course occurs prior to entering residency rather than at the start of clinical rotations for students.^10^

Currently, *MedEdPORTAL* does not offer any instructional activities solely dedicated to teaching written patient assessments for medical students. Most of the relevant *MedEdPORTAL* resources are part of larger educational interventions addressing an array of clinical skills, such as writing an accurate progress note or presenting an entire history and physical (H&P) orally, or are geared for students later in their clinical training.^10–12^

In response to this lack of established curricula on patient assessment writing, we developed a 90-minute, large-group, interactive session for pediatric clerkship students to guide learners through the process of formulating and writing an appropriate patient assessment.

## Methods

The educational skill session was added to the required core educational series during the first week of the pediatric clerkship at McGovern Medical School at the University of Texas Health Science Center. The skill session occurred six times total throughout the year, with each individual student participating once. Forty to fifty students were present per session with one faculty instructor. This educational skill session was novel because its focused content was not explicitly taught anywhere else in the medical student curriculum. The skill session first focused on identifying the essential information from the subjective and objective portions of the patient H&P required to formulate a patient assessment. Then, students practiced interpreting fragments of common subjective and objective patient data using medical terminology and descriptors. Finally, students actively revised examples of written patient assessments using precise medical terms and discussed their differential diagnosis using clinical reasoning.

### Educational Skill Session Development

At our institution, pediatric hospitalist attending physicians are some of the primary physicians who assign and review student H&Ps during the pediatric clerkship rotation. Attending physicians and students cited the patient assessment as the area most needing improvement in the student H&P, which was supported by prior studies.^6^ To address this deficiency, attending physicians developed a template that would provide some structure for the patient assessment portion of the H&P and a variety of material for students to practice prior to being expected to complete a patient assessment, all of which were incorporated into this educational session. Pediatric hospitalist physicians at the stated institution generated all the short sample example prompts throughout the skill session, based on some of the most common types of subjective and objective findings that clinicians encounter. They also wrote the sample H&Ps (Appendices C, D, and E) with comparable moderate complexity to allow students deliberate practice with using medical terminology and descriptors. The sample H&Ps were written intentionally to provoke thought on several plausible differential diagnoses so that students could practice their clinical reasoning.

### Educational Skill Session

The primary teaching materials consisted of the PowerPoint (Appendix A) with its associated instructor script (Appendix B) and adjunctive technology. Although not a requirement of the session, we used the audience response system Poll Everywhere for the session activities since it facilitated interactions with a large group and kept students engaged throughout. The educational skill session began with students writing a patient assessment for a sample H&P (Appendices C, D, and E). The instructors provided one selected sample H&P as a printed document. Students could also access the selected sample H&P in an online learning management system. This activity could also be done prior to the session if time is short.

After students finished writing their assessments, the educational intervention transitioned to an explanation of what an assessment was, its importance, and its essential components, addressing learning objective 1. This portion of the session provided a structured template of a general written patient assessment. Students were given sections of the validated Pediatric History and Physical Exam Evaluation (P-HAPEE) grading rubric^13^ to illustrate components necessary for a complete, effective patient assessment. This rubric demonstrated how students could move from an incomplete to an adequate to an excellent patient assessment (Appendix F).

After defining patient assessment, the instructor then explained the importance of using medical terms and semantic qualifiers in creating a complete patient assessment. Semantic qualifiers in this context meant words or phrases used to describe more precisely a medical problem or diagnosis. To practice using appropriate terminology and semantic qualifiers, students modified fragments or prompts of common subjective and objective patient data by adding more precise language. This activity moved students closer to accomplishing objective 2, as it laid the groundwork for synthesizing relevant patient information into appropriate medical terminology.

In the next activity, students received a fairly complete patient assessment and were asked to identify imprecise and incomplete language and to describe how they would change it. The instructor then demonstrated how those example patient assessments could be made more complete by discussing the students' suggested terminology changes and indicating ways they could further improve their patient assessment statements by examining the anchors for *meets expectations* and *exceeds expectations* according to the P-HAPEE rubric.^13^ Students had an active discussion with the instructor about differential diagnoses for these sample full patient assessments, with prompts to provide their rationale for those diagnoses and demonstrate their clinical reasoning. By engaging in dialogue about the reasons they chose particular semantic qualifiers and terminology, as well as differential diagnoses, students were able to demonstrate mastery of objective 2 and build towards achieving objective 3.

In the final group activity, students read a sample H&P, and a volunteer presented their patient assessment. The rest of the students offered suggestions to improve that patient assessment and demonstrated their clinical reasoning by discussing how various differential diagnoses could be supported by the stated information. To conclude the session, students formulated a second written patient assessment for the same H&P they had used at the beginning of the session (Appendix C, D, or E). After they finished, the instructor displayed examples of an appropriate patient assessment for the sample H&P (Appendix G) and led a discussion on other differential diagnoses with their associated clinical reasoning. By applying all the skills the educational session gave them and then utilizing higher-order questioning to check for accurate reasoning, students demonstrated their understanding not only of objective 3 but also of the preceding objectives.

The instructor provided a crossword puzzle for extra practice with medical semantics (Appendix H) and, a week later, sent students the answers to the crossword puzzle (Appendix I) as well as the general template for a written patient assessment. The schedule below provides the suggested timing for each component of the educational skill session, along with the corresponding slides in the PowerPoint presentation (Appendix A):

• At the beginning of the session, students write/type a patient assessment for one of the provided sample H&Ps (Appendix C, D, or E): 20 minutes.
• State the learning objectives of the session (slides 3–4): 4 minutes.
• Define what a patient assessment is and its importance (slide 5): 1 minute.
• Identify essential components of a written patient assessment using a general structured template (slide 6): 3 minutes.
• Transition to discuss what makes an assessment good versus what makes it better (slide 7); reference Appendix F grading tool: 3 minutes.
• Define medical terms and semantic qualifiers (slide 8): 1 minute.
• Practice synthesizing subjective information often encountered when obtaining a patient's history using Poll Everywhere (slides 9–18): 6 minutes.
• Practice interpreting objective information often encountered when reviewing a patient's physical and diagnostic studies using Poll Everywhere (slides 19–31): 7 minutes.
• Review full assessment examples and practice making them better with precise synthesizing medical terms using Clickable Image functionality in Poll Everywhere (slides 32–33); discuss differential diagnosis for the full assessment examples and utilize clinical reasoning to support or refute stated differential by students (slides 32–33): 10 minutes.
• Review a full H&P; ask class to state a full assessment, critiquing how to make it better by using medical terms; and discuss differential diagnosis using clinical reasoning (slides 34–35): 10 minutes.
• At the end of the session, students write/type another assessment for their provided sample H&P (Appendix C, D, or E); answers to samples are provided in the teaching PowerPoint (Appendix A) as well as separately (Appendix G): 15 minutes.
• Discuss patient assessment for the sample H&P and differential diagnoses using clinical reasoning (Appendix G; slides 37–39): 5 minutes.

### Evaluation of the Educational Skill Session

To evaluate the educational skill session's effectiveness in improving students' clinical skills in assessment writing, we collected the two written patient assessments the students completed at the beginning and the end of the educational skill session. These anonymous unpaired patient assessments were then scored by three blinded attending physicians using portions of the validated P-HAPEE (Appendix F).^13^ We chose to use this rubric because it had been validated and previously assessed for very good overall interrater reliability, as well as very good interrater reliability when using individual sections/questions.^14^ Rubric sections/questions 8 and 9 (Assessment and Differential Diagnosis, respectively) were used to assess information synthesis and clinical reasoning (Appendix F).

Each grader attended one of the educational skill sessions during the academic year. Following completion of all the skill sessions, the graders were trained to use these specific portions of the rubric by completing a 1-hour training session in which they were provided with the general patient assessment template that was given to students, copies of the sample H&Ps for reference, the patient assessment examples that correlated with each sample H&P, and the selected portions of the rubric itself. During the training session, graders worked through several unidentified written patient assessments from the data bank of completed patient assessments by the students. Each section on the rubric had a scaled score, with 1 being *poor quality* or *below expectations* and 5 being *excellent quality* or *exceeding expectations.* In the training session, these patient assessments were first individually graded using the specific portions of the rubric. Graders then discussed their scores as a group and analyzed how and why their scores differed. Following this discussion, some graders preferred to change their scores, which reflected increased conformity through training. After this training session, graders were each provided with all the random unidentified written patient assessments to score on their own without any further discussion among themselves. The scores were analyzed for statistical significance using the Kruskal-Wallis test. Interrater reliability was described using both percent agreement and kappa coefficients. Intrarater reliability was not analyzed. Results were also stratified for those students who attended the session after already having had some clerkship experience.

We chose to stratify for clerkship experience because it was one of the most impactful variables that could indirectly improve students' clinical skills in patient assessment formulation. Specifically, we stratified for those students attending the skill session already having completed the internal medicine clerkship as this was the longest core clerkship third-year students completed at our institution with the most exposure to H&Ps. Because of the length of this clerkship, students would have had the most opportunity to receive indirect and/or direct teaching from an attending or resident, as well as more practice formulating patient assessments, because of higher patient loads per student. A variable that could not be controlled for was prior teaching from a clinical educator because it was difficult to infer what type of teaching the students might have received and what kind of effect that might have had on their written patient assessment skills.

## Results

Of the 250 students who attended the session in 2018–2019, 238 (95%) and 202 (93%), completed the pre- and postsession patient assessments, respectively (*p* = .26). Due to our clerkship schedule structure, students took the pediatrics clerkship in one of six blocks during the academic year, giving them variable amounts of clinical experience prior to participating in this session. Using the P-HAPEE rubric,^13^ we found a statistically significant increase in learning assessment and differential diagnosis scores following the skill session intervention (Table 1). The skill session had a greater effect size compared to the increase with clinical experience. Kappa coefficient scores showed fair interrater reliability for patient assessment scores and substantial agreement for differential diagnoses scores (Table 2).

**Table 1.**
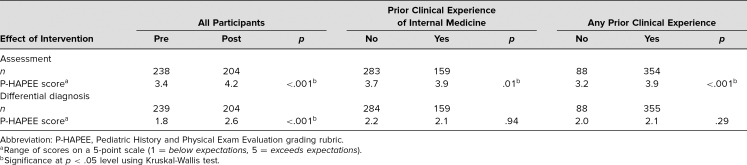
Effect of Intervention on Skill of Formulating Assessment and Differential Diagnosis

**Table 2.**
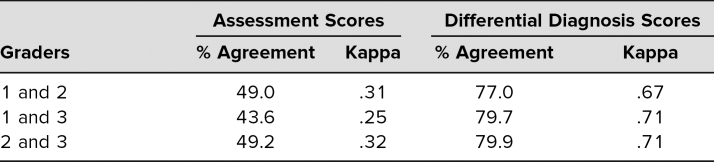
Interrater Reliability Among Assessment and Differential Diagnosis Scores (*n* = 443)

Ninety-eight percent of students found the skill session valuable despite prior clinical experience. Among all of the skill session attendees, regardless of prior clinical experience, 50% (125 of 250) self-reported that they had received no prior instruction on how to formulate an assessment. Students voiced appreciation for this focused skill session and also identified the absence of formal patient assessment writing instruction as a curriculum gap in their preclinical and clinical medical student education, as demonstrated by the identified themes and sample comments below:

• Curricular teaching gap:
  ○ “We have never been taught this so I've just been copying the format of previous notes.”
  ○ “Really appreciated opportunity to work on skills that we rarely have been taught/walked through. Really helpful session.”
  ○ “This was really, really helpful but would have been great in transition to clerkships instead of having to figure a lot of it out through trial and error.”
  ○ “Thank you for doing this workshop. Even though it's our last rotation of 3rd year, I never really knew what to write for assessment and now this workshop addresses that.”
• Structured methodology of teaching:
  ○ “Clear instructions as to how to form an assessment! It was SO nice to have an actual session on this topic—rather than just random feedback throughout the rotation.”
  ○ “Gives us a systematic way to think about an assessment and what components to include.”
  ○ “It taught us a concrete method of composing an assessment.”

## Discussion

To address the lack of established curricula on patient assessment writing, we developed a 90-minute, large-group, interactive session for pediatric clerkship students to guide learners through the process of formulating and writing an appropriate patient assessment. Students demonstrated a significant positive impact in this key clinical skill following the session, with objective analysis of assessment and differential diagnosis scores showing a statistically significant increase.

### Reflective Critique

One of the challenges we encountered in implementing this session was finding 90 minutes of instructional time in an already-full clerkship schedule. Given the session's emphasis on higher-order questioning, application, and critical thinking, we felt the longer session time was warranted. However, other institutions may find it more feasible to have students complete the pre- and postsession patient assessment writing activity outside the session time.

This educational session focuses only on applying patient assessment writing skills to pediatric scenarios, but the skill of writing appropriate patient assessments is universal. Because we received so much feedback from students about the positive impact of this session and a desire to learn this information sooner, we have been working with curriculum leadership to implement this educational skill session during students' weeklong Transition to Clerkship course, which immediately precedes the start of their clerkship year. This version of the session will include patient assessment practice examples from a variety of specialty areas to broaden its scope and applicability.

Using isolated sections of the P-HAPEE^13^ tool worked well as an evaluation metric and continues to provide standardized guidance for students and for attending physicians who review student H&Ps. Explanations for the interrater reliability being only fair for scores of the patient assessment portion in our study may be due to having only three graders, rather than a minimum of 19 as in prior studies where there was very good agreement in patient assessment scores.^13,14^ Also, the patient assessment portion of a write-up requires subjective and objective data interpretation, making it inherently the most difficult portion to grade.^15^

The results of this skill session do have some limitations. First, due to the anonymous nature of the surveys, observations could not be paired to see a direct effect of the skill session on each individual's performance. Second, we did not assess longitudinal skill retention since it was difficult to control for extraneous variables such as students receiving informal feedback or unknown structured or unstructured teaching from other residents or attending physicians.^16^ Fifty percent of students reported they had not received any instruction on writing patient assessments prior to the session; however, because this is a self-reported number, we cannot be sure that it is a true reflection of students' clinical instruction.

Additionally, students at our institution take their clerkships in a variable order, meaning some students reach the pediatrics clerkship with a great deal of clinical experience while others come with no experience. This is why we stratified the results for clerkship rotations completed, if any, in the analysis of this educational intervention rather than the time of year. Another factor that could have contributed to the gains seen in scores could be repeating the same exercise in and of itself; however, because patient assessment formulation requires higher-order learning application rather than recall, this factor was deemed not to be a significant influence on the results.

Finally, we recognize that because our intervention was not held at a single point in time for all students, those who took the clerkship and received the training earlier in the academic year had an advantage over their peers and could have received higher clinical evaluation scores. Other institutions that implement this intervention may consider how the timing of the session could impact students' performance throughout their clerkships and what skill level is appropriate for a third-year medical student. The authors of the P-HAPEE stated that they would expect students to meet expectations, a score of 3, at the end of their third year.^13^ In our anecdotal experience, attending physicians generally expect a third-year student to be able to perform patient assessment at the level of a P-HAPEE^13^ score of 3 to meet expectations for the clerkship rotation and a 5 to exceed expectations in most summative student core clerkship evaluations pertaining to the core competency of Interpersonal and Communication Skills.^4,17^

We attempted to establish content validity by identifying student learning objectives for the session and engaging in instructional methods that required students to demonstrate mastery of the learning objectives. Additionally, pediatric hospitalists created and reviewed our sample H&Ps for moderate complexity and multiple possible differential diagnoses to ensure that students would be presented with appropriately constructed content. We did not address response process validity since this was the first iteration of the educational session, but we would include this measure in future interventions.

### Next Steps and Future Directions

In future iterations, the skill session could be adjusted in its methodology to account for level of knowledge as students gain more clinical experience. For example, students who had no clinical experience appreciated emphasis on learning objective 2, covering short fragment examples of subjective and objective information; however, students who had clinical experience felt that less time could be spent on the short examples and more time on objective 3, modifying full assessments and practicing clinical reasoning in discussion of differential diagnoses. Additionally, the scope of the session could be broadened to include example patient assessments from other specialties, which would also help to identify subtle differences in how patient assessments are written across specialties. To improve clinical communication skills in all aspects, this session could be paired with another one that gives students practice stating their patient assessments using medical terminology and then using conversational language appropriate for patients' understanding.

## Appendices

PowerPoint Presentation.pptxInstructor Script.docxSample H&P 1.docxSample H&P 2.docxSample H&P 3.docxP-HAPEE Isolated Scoring Tool.docxAssessment Examples for Sample H&Ps.docxMedical Semantics Crossword.pdfCrossword Puzzle Answers.docx
All appendices are peer reviewed as integral parts of the Original Publication.
