# Supplementary material for: Skill Session on Writing Patient Assessments for Pediatric Clerkship Students
Source: MedEdPORTAL. 2020 Nov 9;16:11029. doi: 10.15766/mep_2374-8265.11029 (PMC7666838; doi:10.15766/mep_2374-8265.11029)
Supplement: Supplementary file 1 — PowerPoint Presentation.pptxInstructor Script.docxSample H&P 1.docxSample H&P 2.docxSample H&P 3.docxP-HAPEE Isolated Scoring Tool.docxAssessment Examples for Sample H&Ps.docxMedical Semantics Crossword.pdfCrossword Puzzle Answers.docx [file mep_2374-8265.11029-s001.zip › A. PowerPoint Presentation.pptx]

## Slide 1
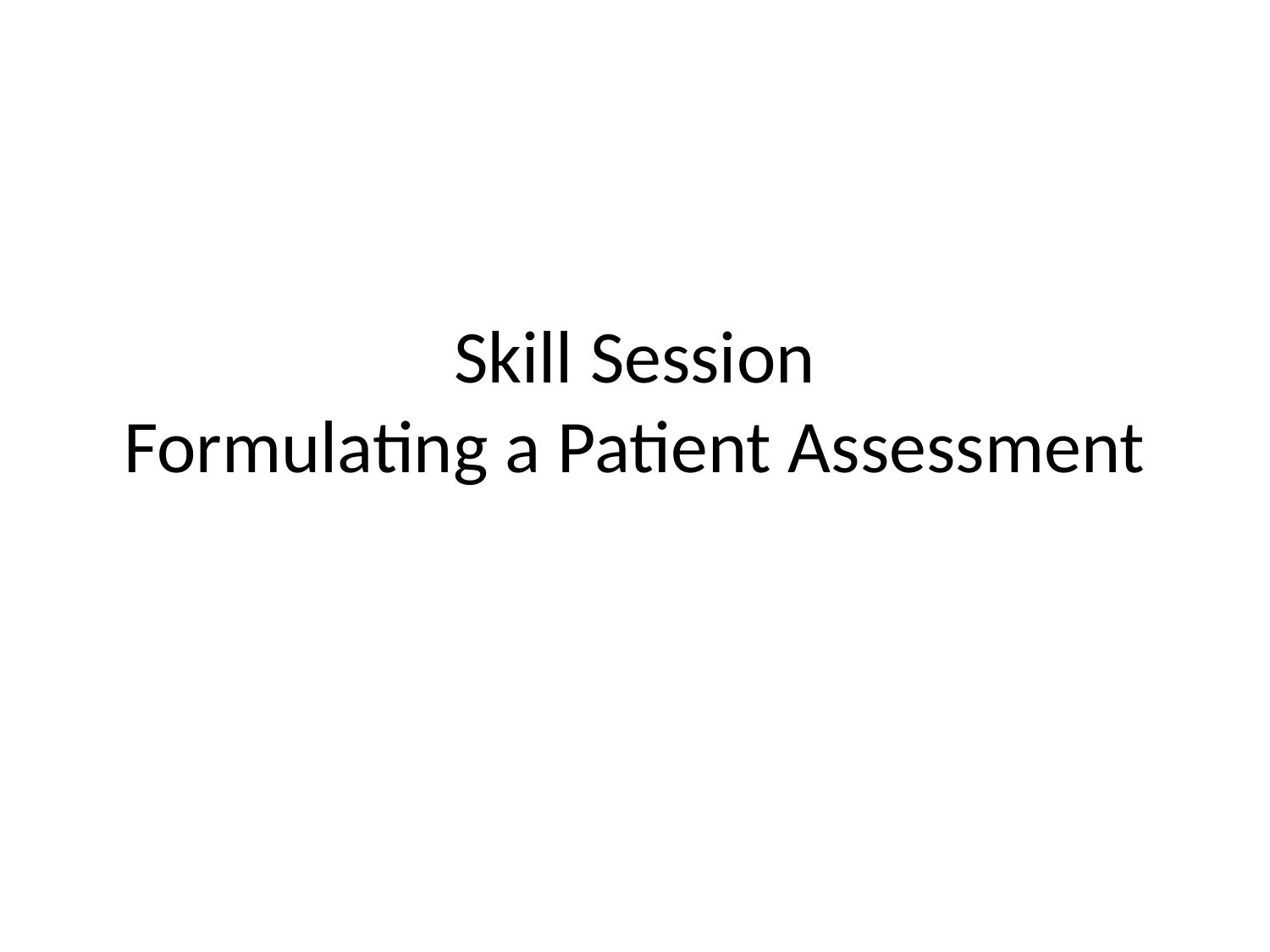

# Skill Session Formulating a Patient Assessment

## Slide 2
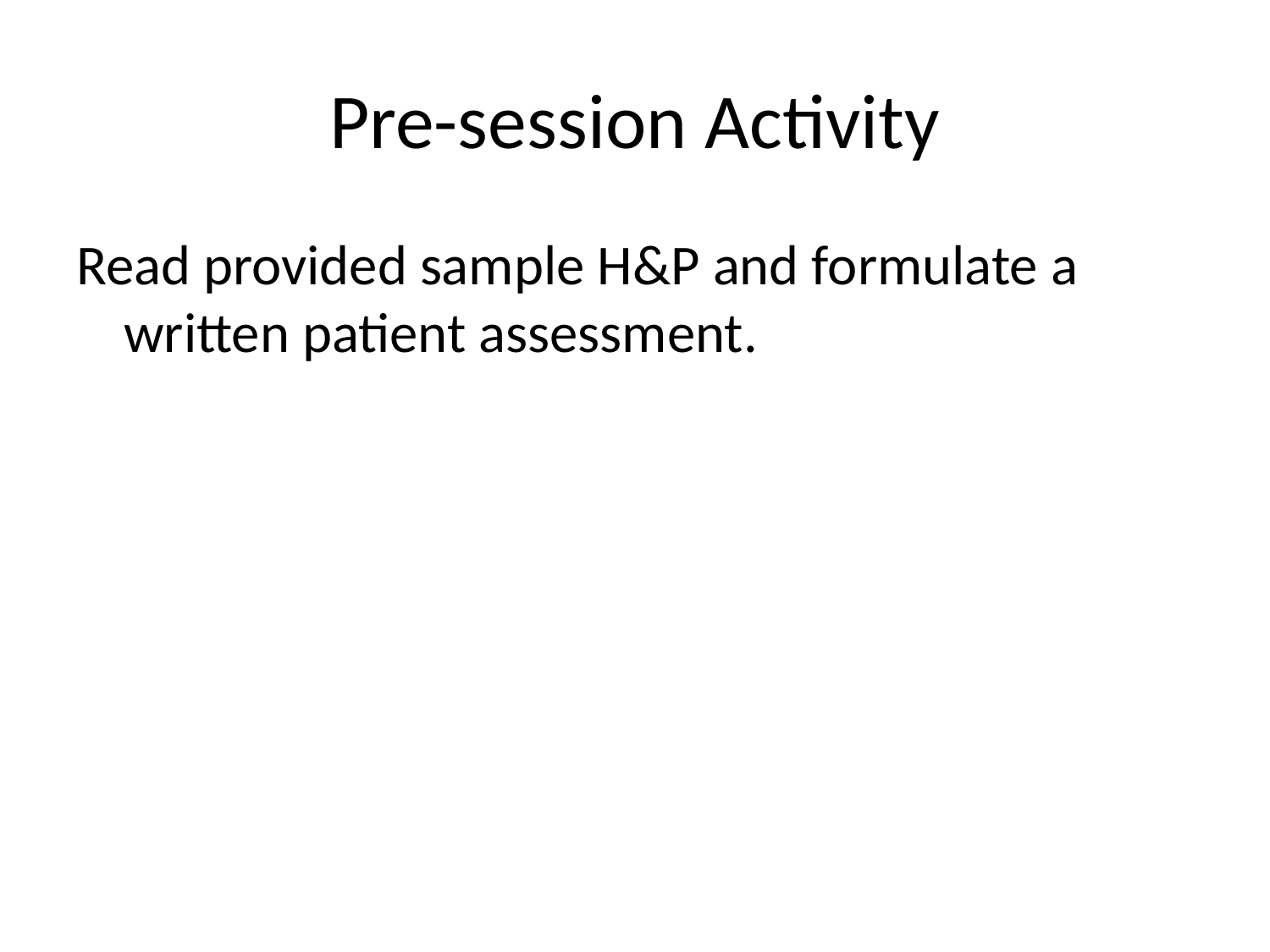

# Pre-session Activity
Read provided sample H&P and formulate a written patient assessment.

## Slide 3
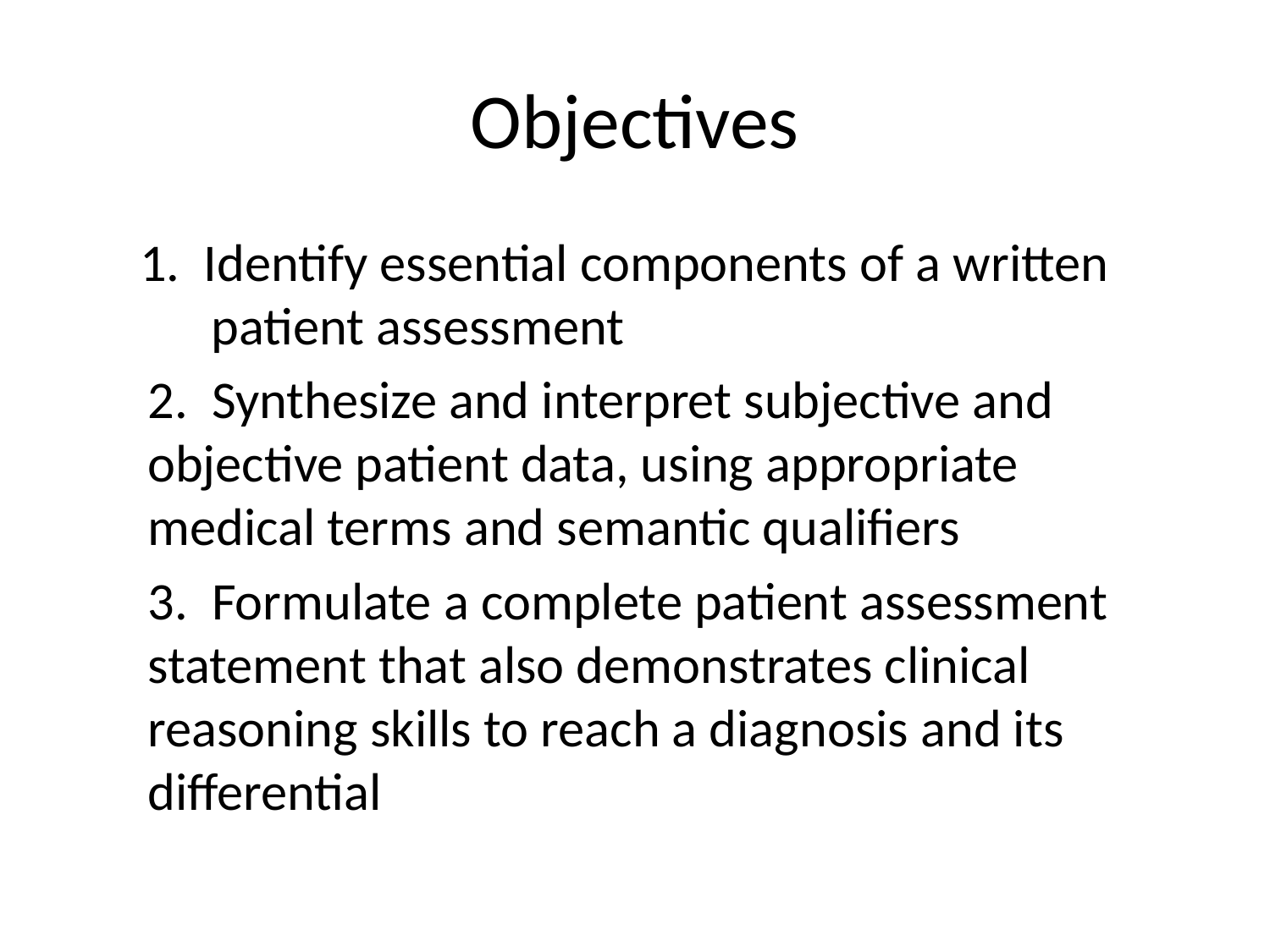

# Objectives
1. Identify essential components of a written patient assessment
	2. Synthesize and interpret subjective and 	objective patient data, using appropriate 	medical terms and semantic qualifiers
	3. Formulate a complete patient assessment 	statement that also demonstrates clinical 	reasoning skills to reach a diagnosis and its 	differential

## Slide 4
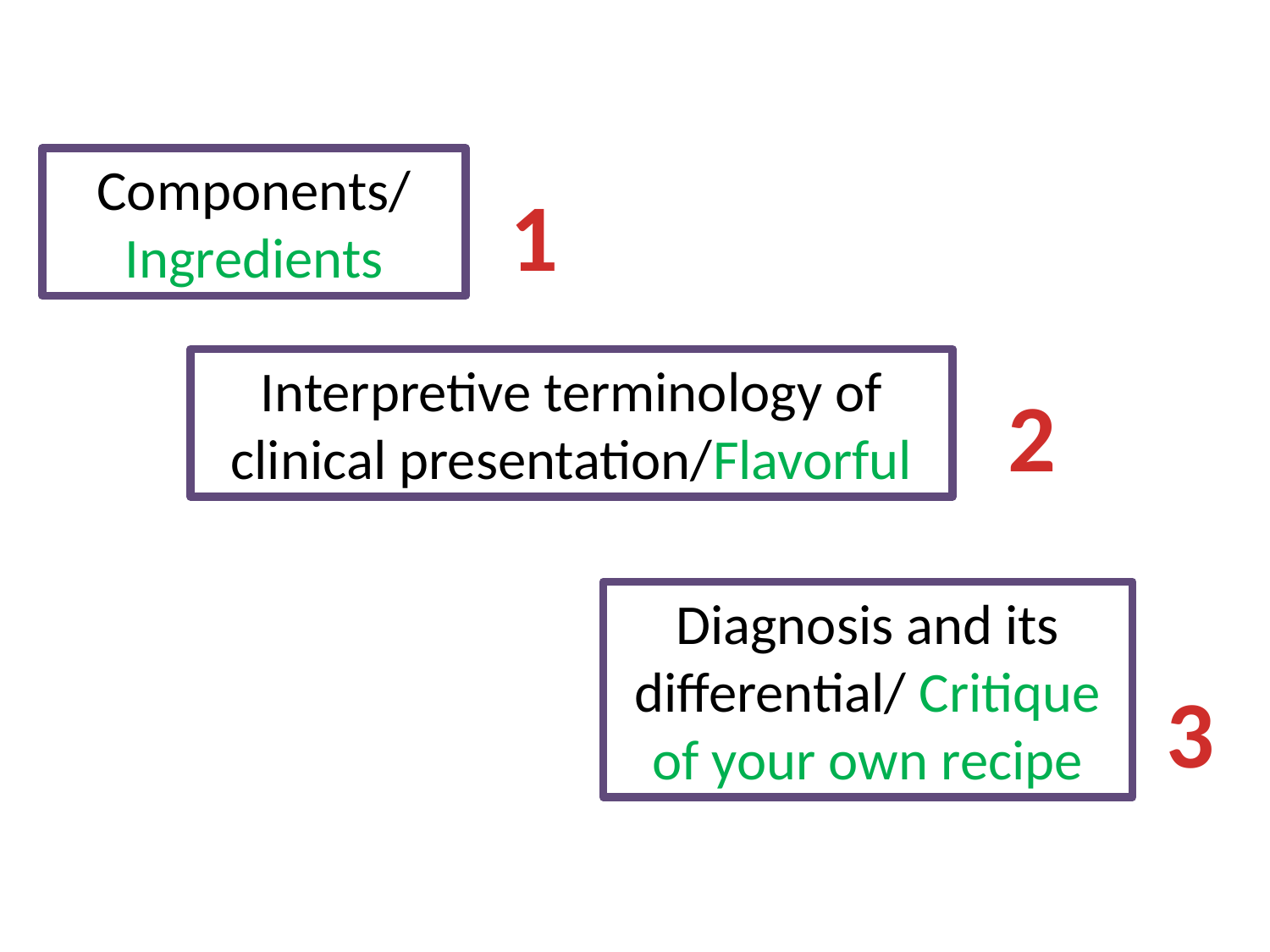

Components/
Ingredients
1
Interpretive terminology of clinical presentation/Flavorful
2
Diagnosis and its differential/ Critique of your own recipe
3

## Slide 5
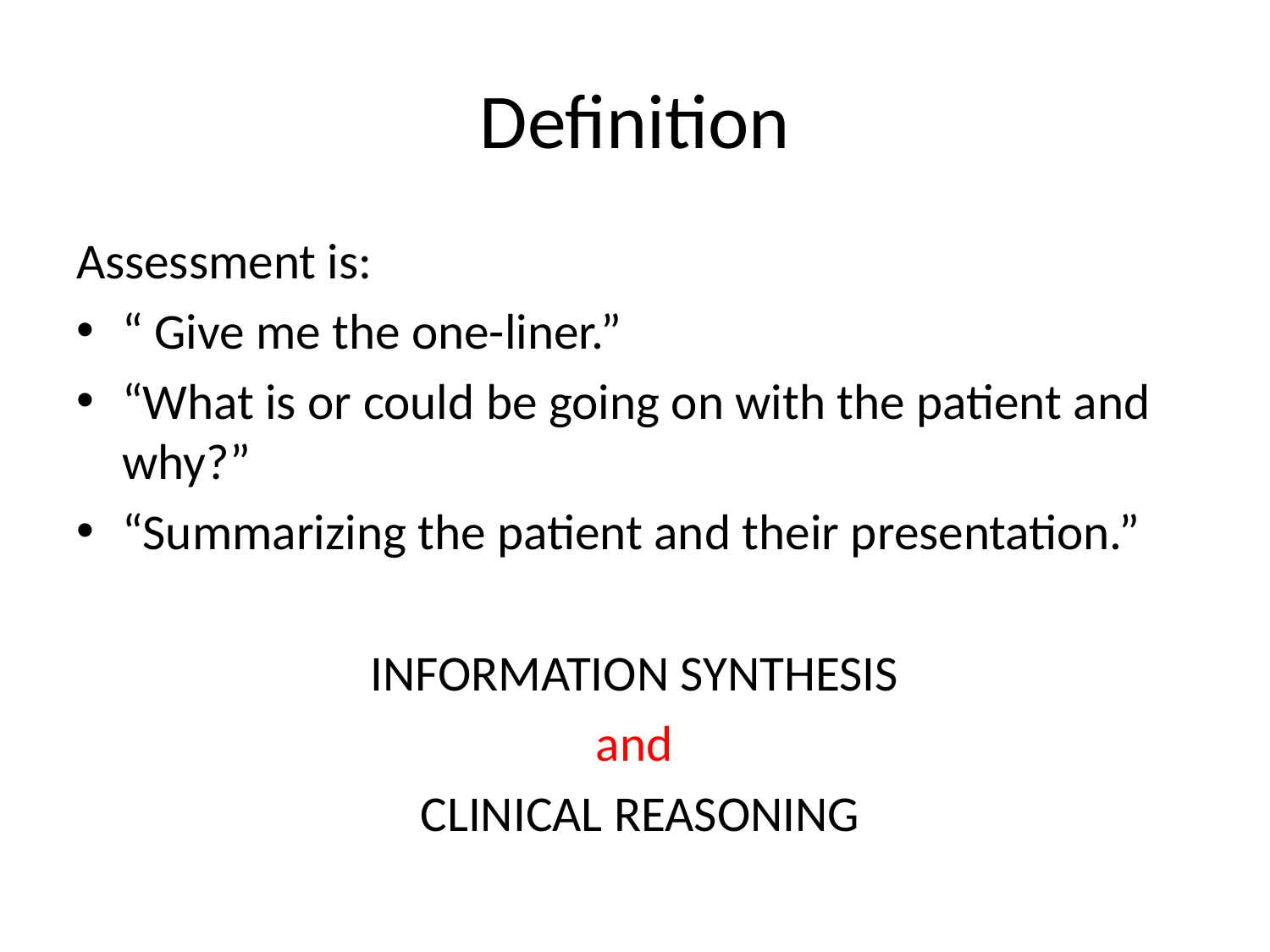

# Definition
Assessment is:
“ Give me the one-liner.”
“What is or could be going on with the patient and why?”
“Summarizing the patient and their presentation.”
INFORMATION SYNTHESIS
and
CLINICAL REASONING

## Slide 6
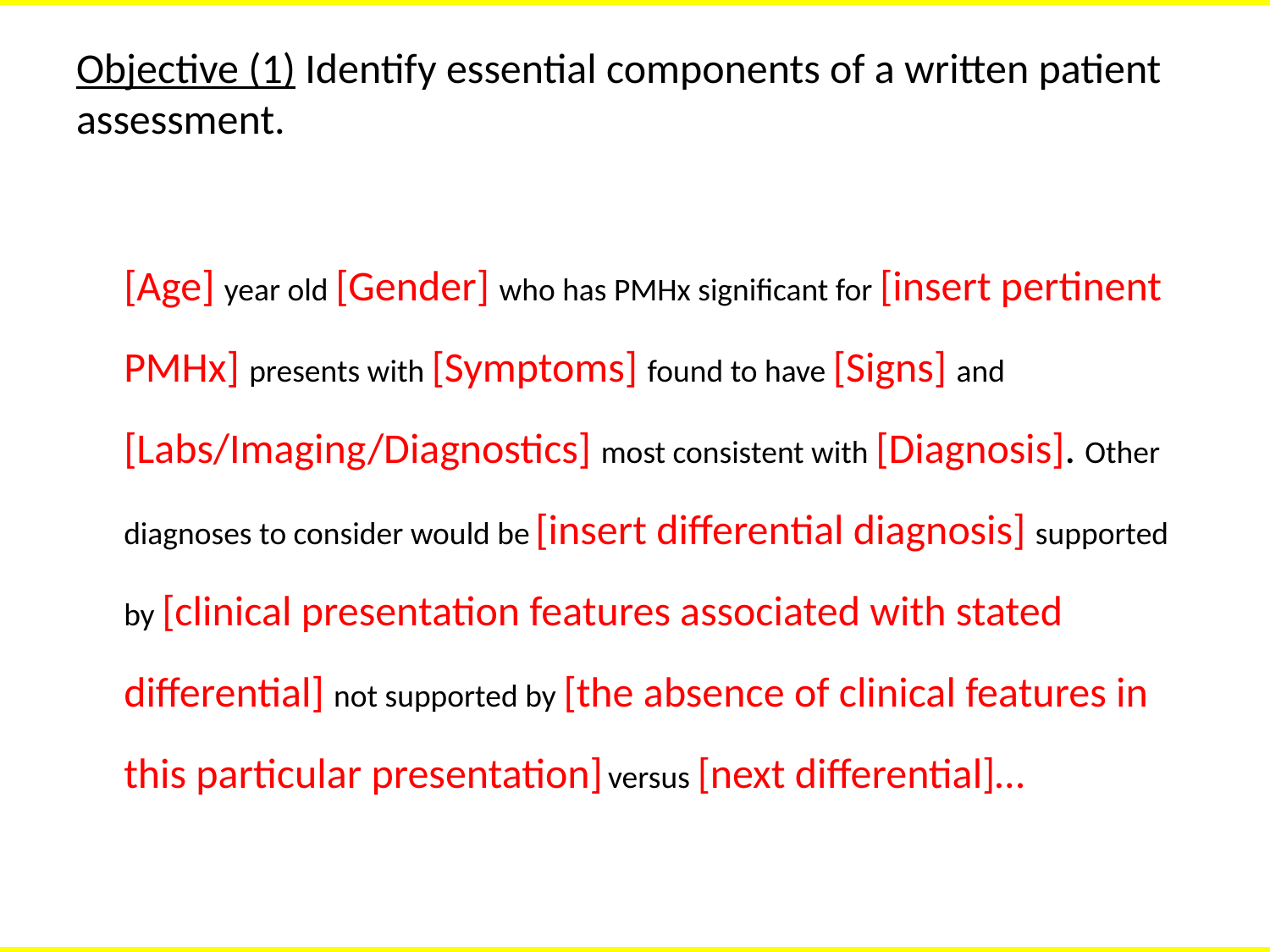

# Objective (1) Identify essential components of a written patient assessment.
	[Age] year old [Gender] who has PMHx significant for [insert pertinent PMHx] presents with [Symptoms] found to have [Signs] and [Labs/Imaging/Diagnostics] most consistent with [Diagnosis]. Other diagnoses to consider would be [insert differential diagnosis] supported by [clinical presentation features associated with stated differential] not supported by [the absence of clinical features in this particular presentation] versus [next differential]…

## Slide 7
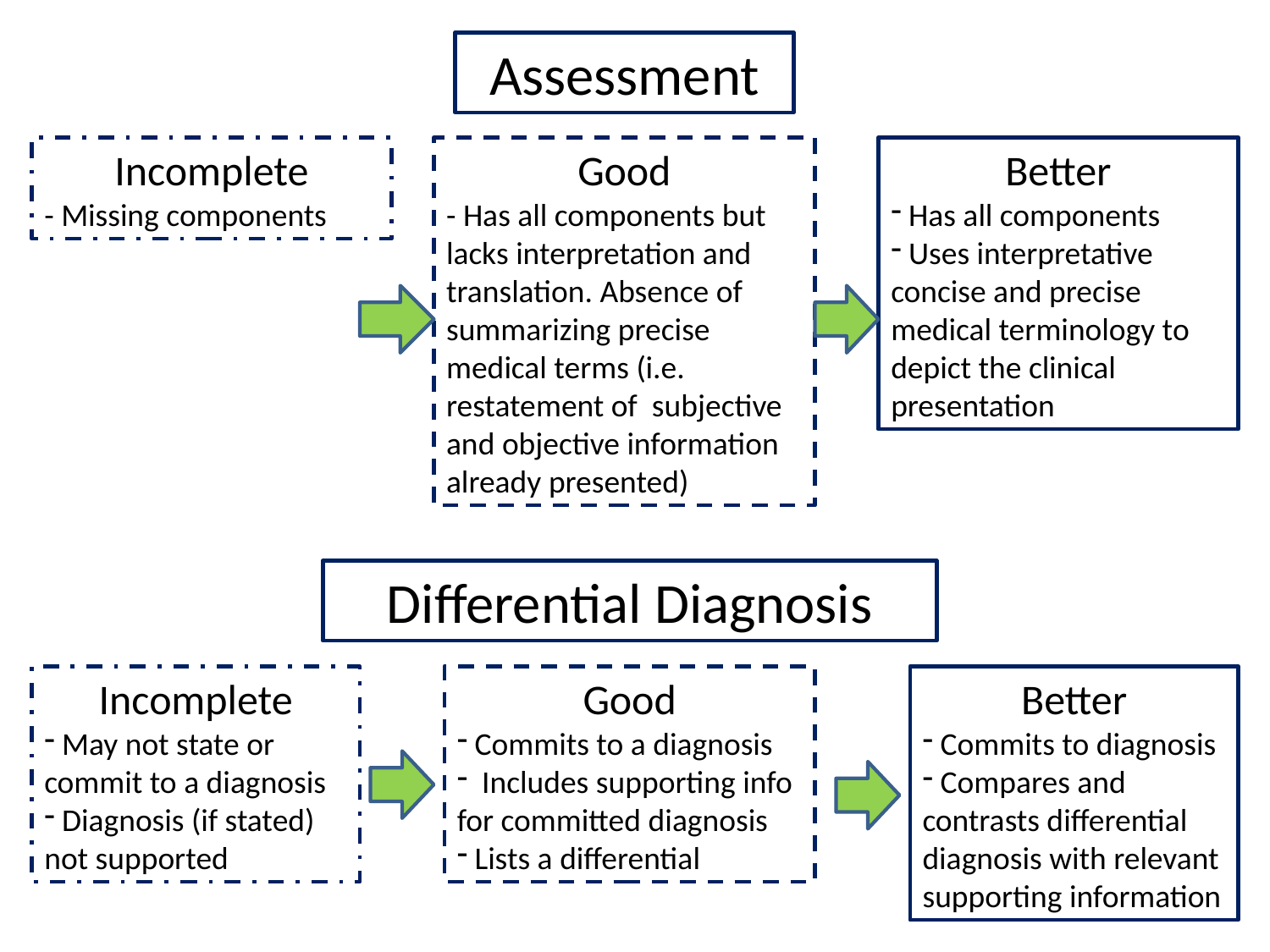

Assessment
Incomplete
- Missing components
Better
 Has all components
 Uses interpretative concise and precise medical terminology to depict the clinical presentation
Good
- Has all components but lacks interpretation and translation. Absence of summarizing precise medical terms (i.e. restatement of subjective and objective information already presented)
Differential Diagnosis
Incomplete
 May not state or commit to a diagnosis
 Diagnosis (if stated) not supported
Good
 Commits to a diagnosis
 Includes supporting info for committed diagnosis
 Lists a differential
Better
 Commits to diagnosis
 Compares and contrasts differential diagnosis with relevant supporting information

## Slide 8
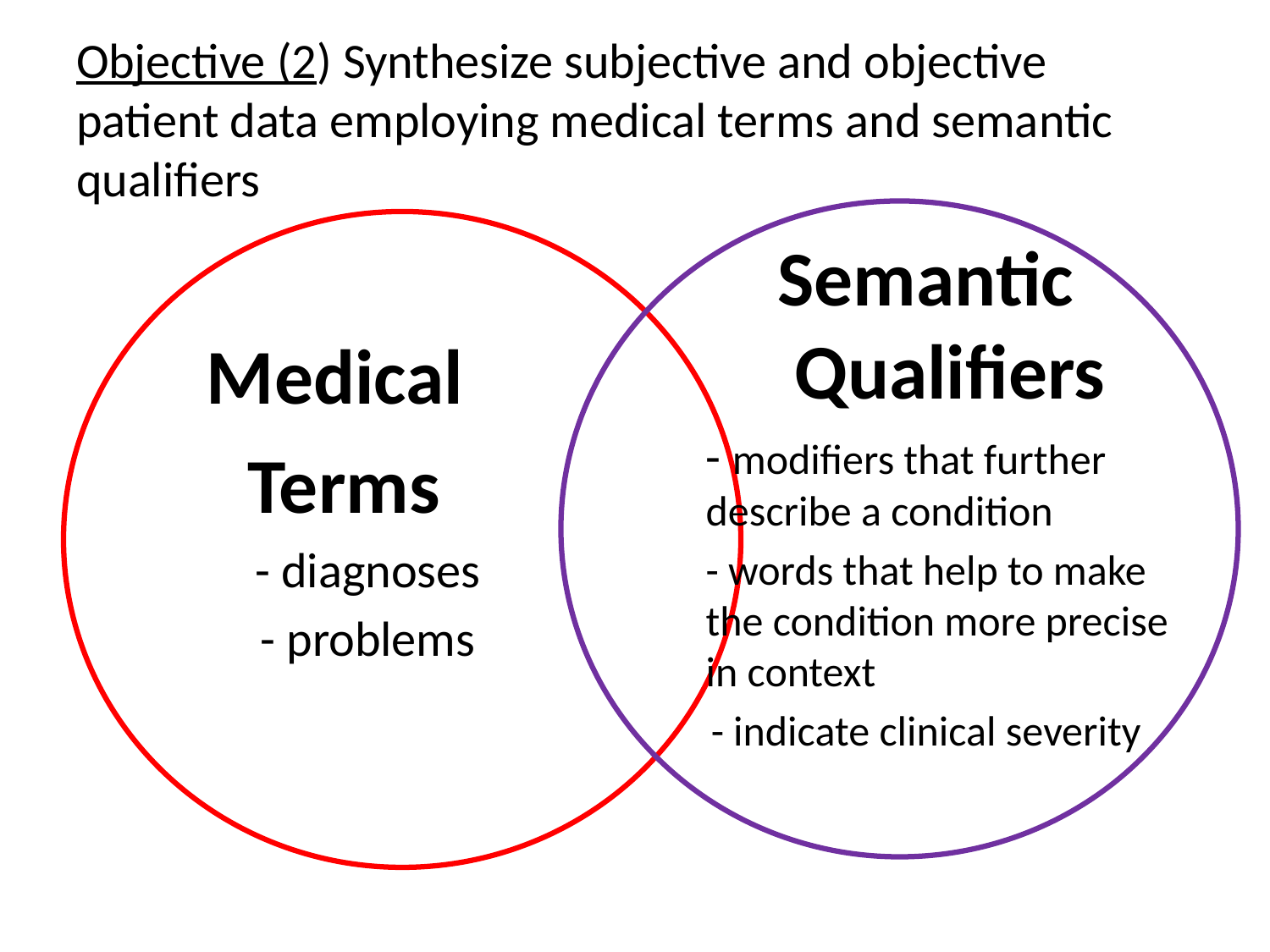

# Objective (2) Synthesize subjective and objective patient data employing medical terms and semantic qualifiers
Medical
Terms
	- diagnoses
	- problems
Semantic Qualifiers
	- modifiers that further describe a condition
	- words that help to make the condition more precise in context
- indicate clinical severity

## Slide 9
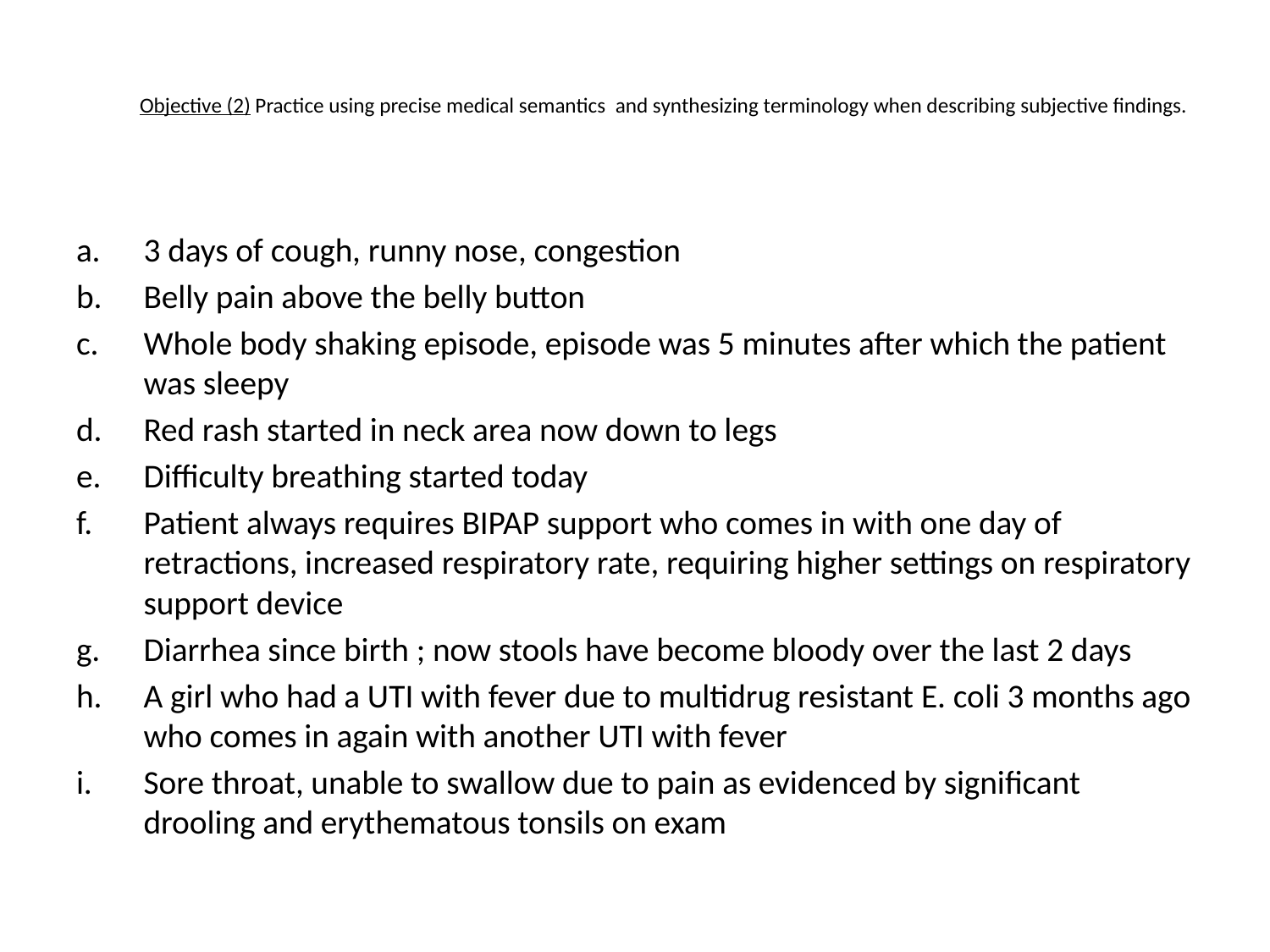

# Objective (2) Practice using precise medical semantics and synthesizing terminology when describing subjective findings.
3 days of cough, runny nose, congestion
Belly pain above the belly button
Whole body shaking episode, episode was 5 minutes after which the patient was sleepy
Red rash started in neck area now down to legs
Difficulty breathing started today
Patient always requires BIPAP support who comes in with one day of retractions, increased respiratory rate, requiring higher settings on respiratory support device
Diarrhea since birth ; now stools have become bloody over the last 2 days
A girl who had a UTI with fever due to multidrug resistant E. coli 3 months ago who comes in again with another UTI with fever
Sore throat, unable to swallow due to pain as evidenced by significant drooling and erythematous tonsils on exam

## Slide 10
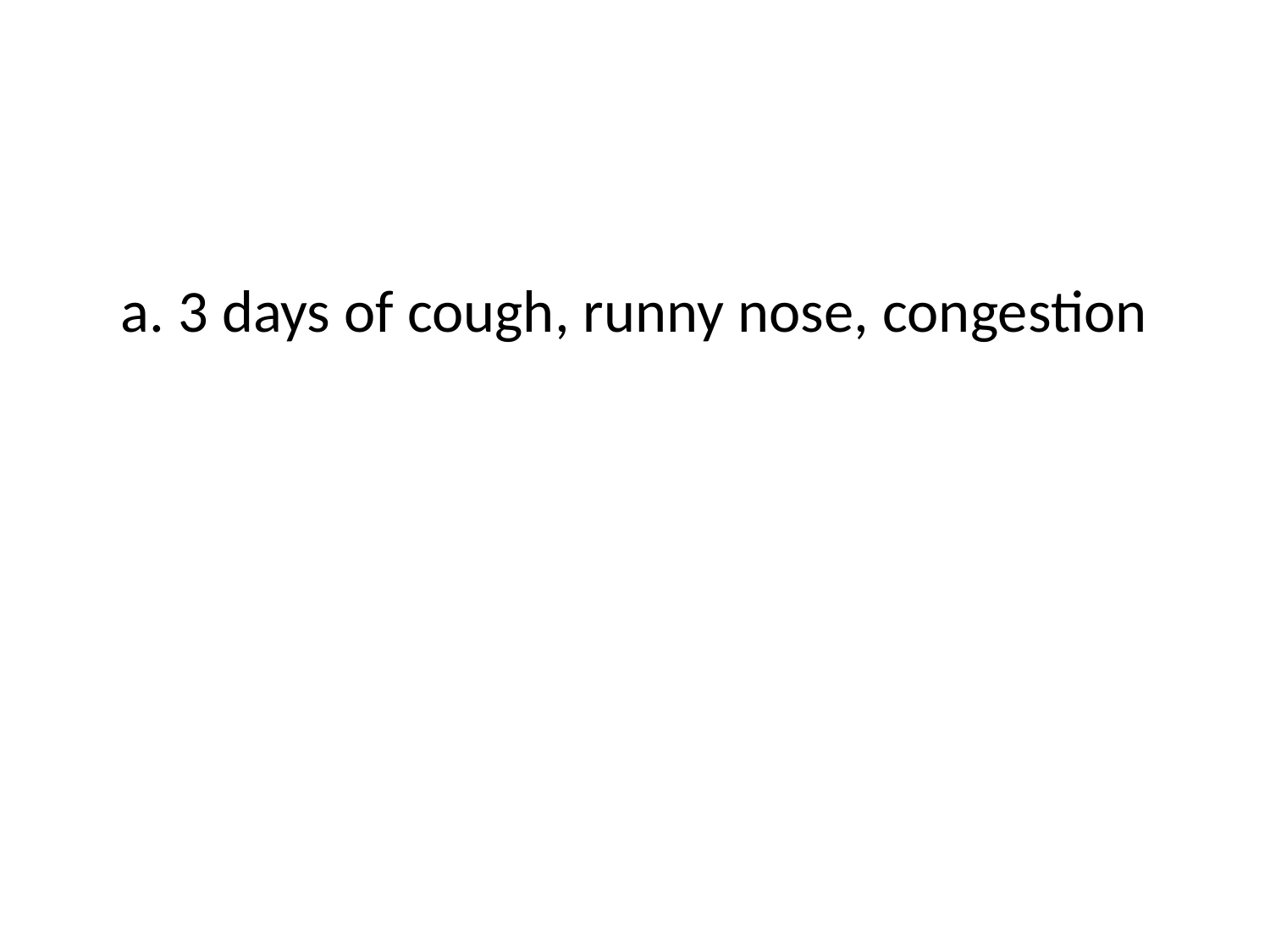

# a. 3 days of cough, runny nose, congestion

## Slide 11
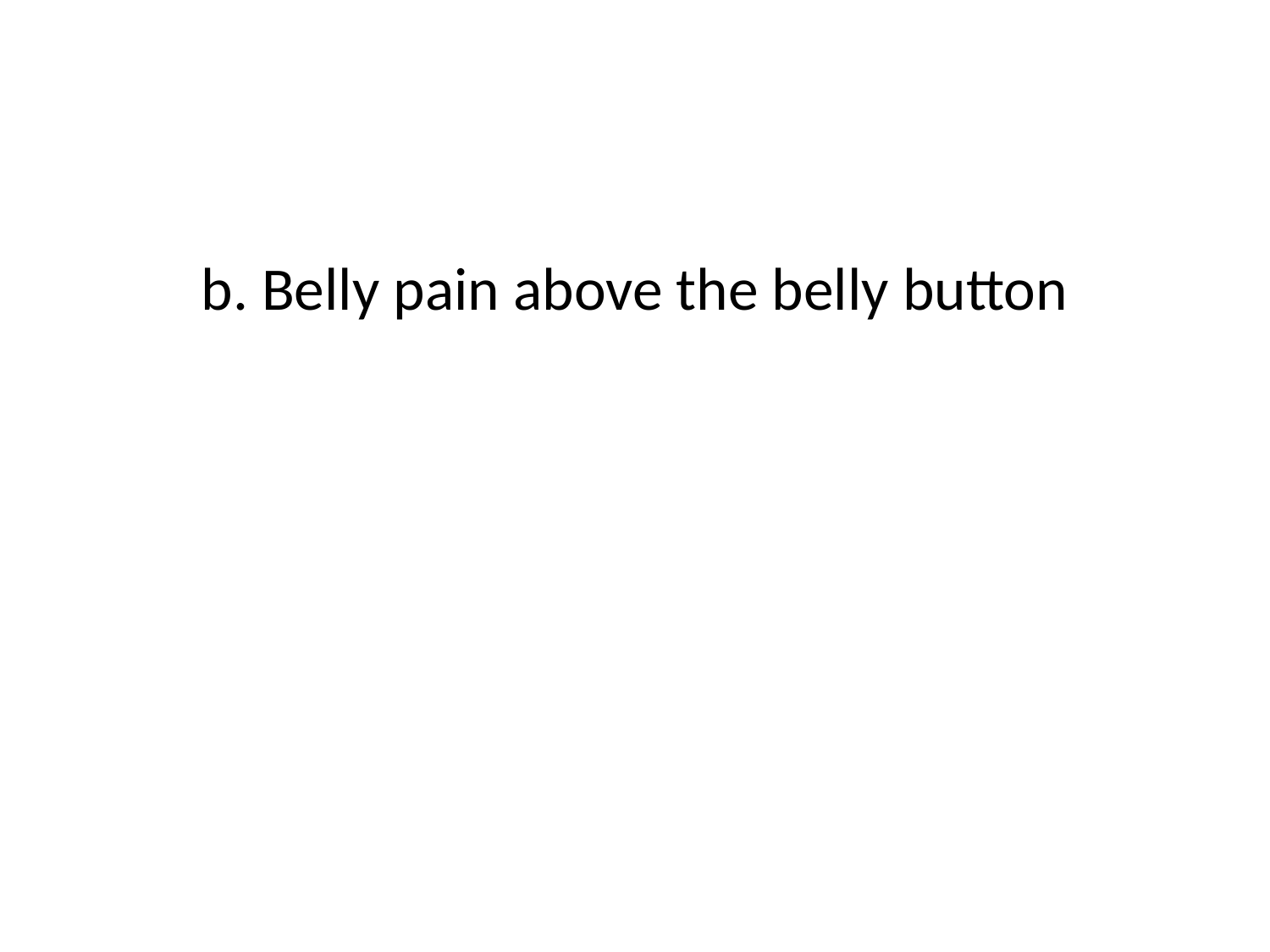

# b. Belly pain above the belly button

## Slide 12
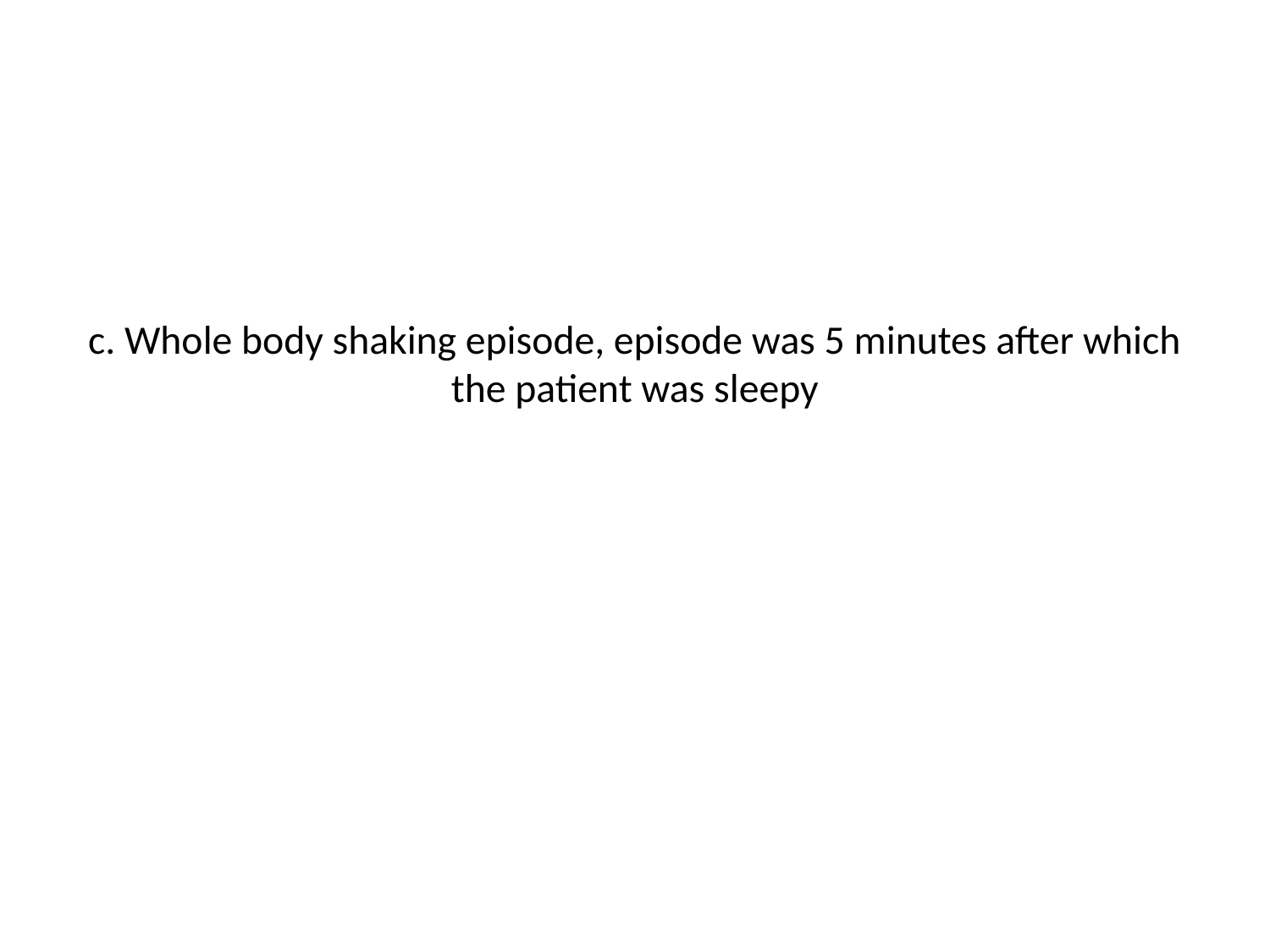

# c. Whole body shaking episode, episode was 5 minutes after which the patient was sleepy

## Slide 13
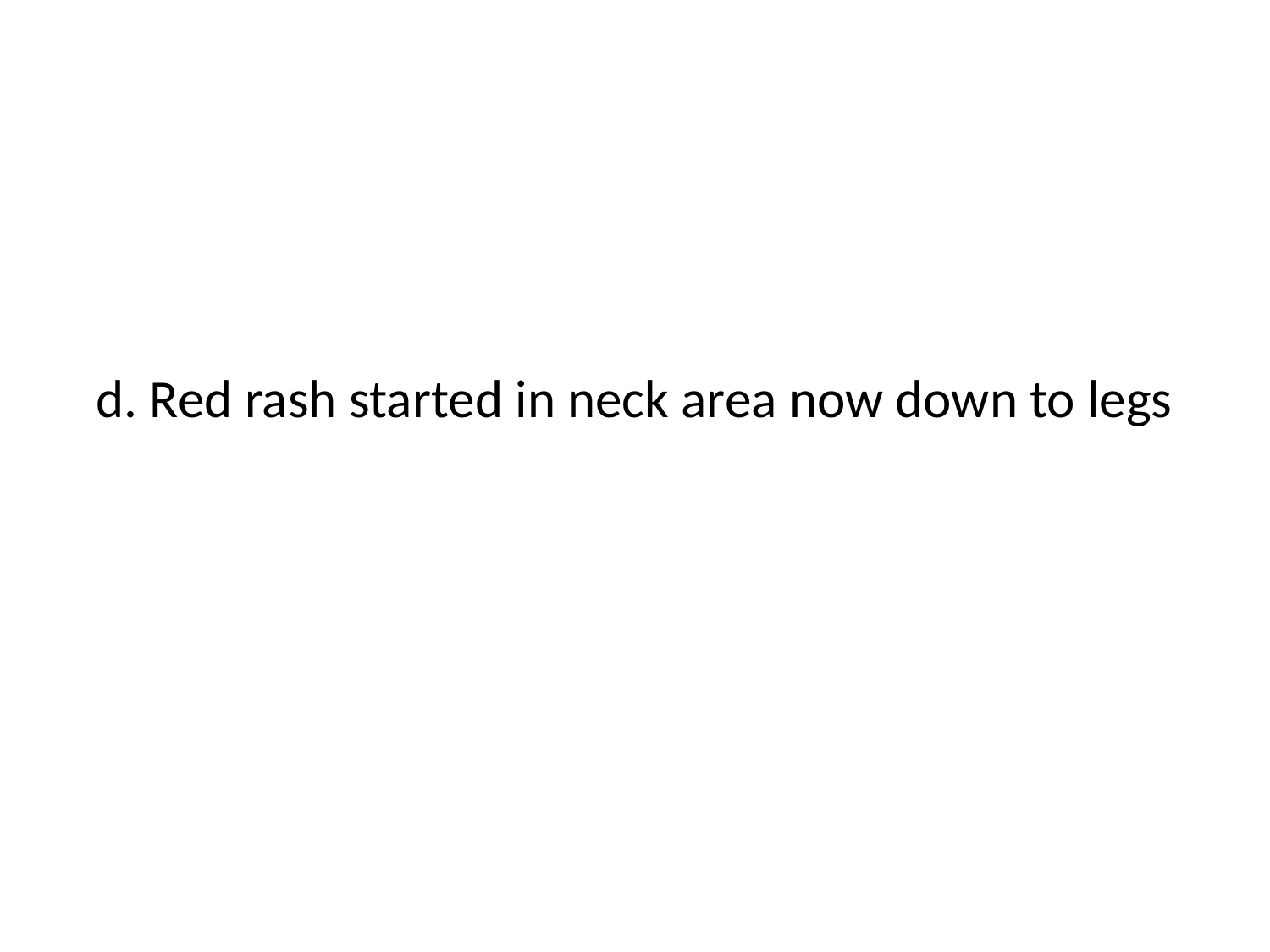

# d. Red rash started in neck area now down to legs

## Slide 14
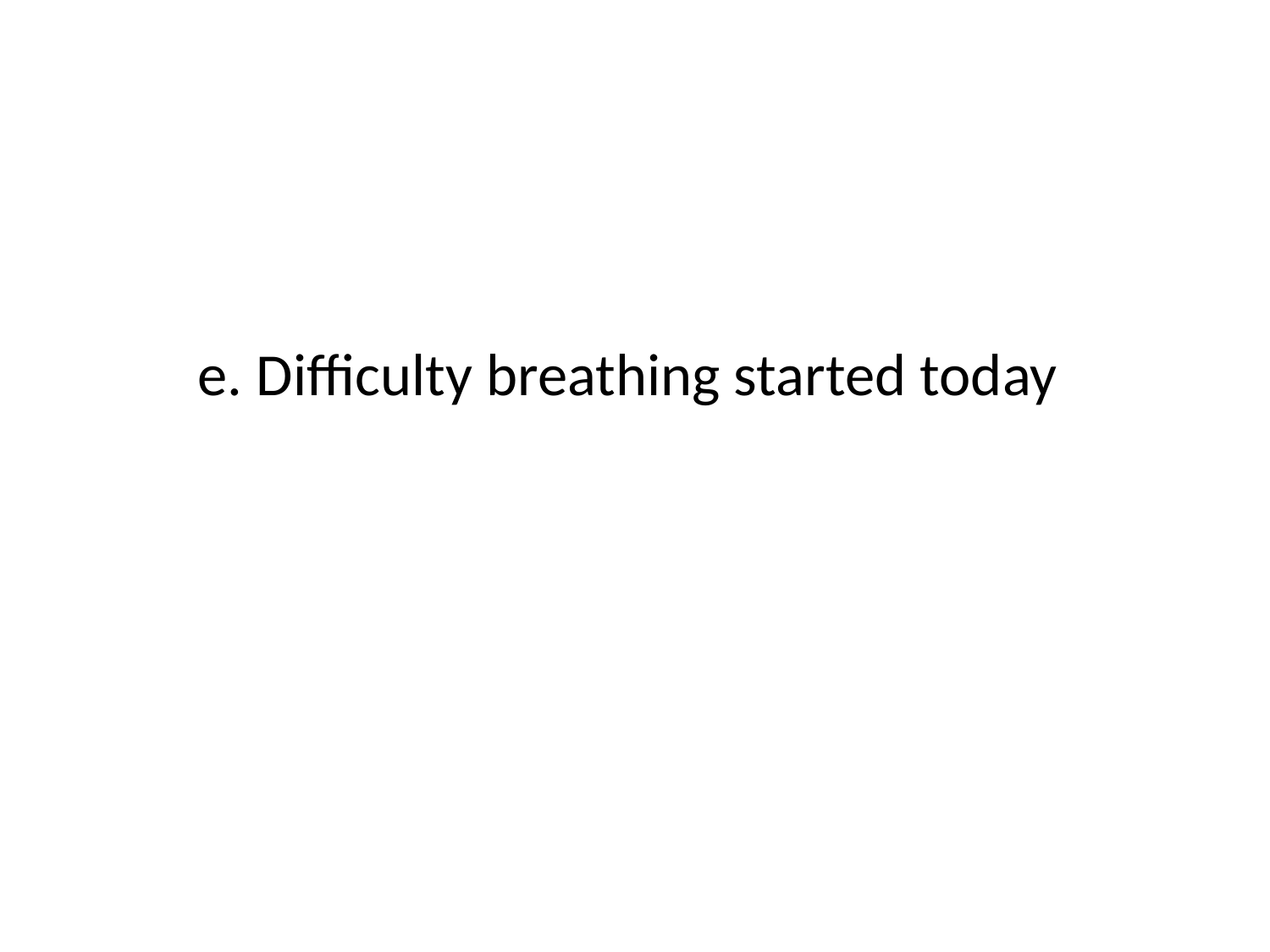

# e. Difficulty breathing started today

## Slide 15
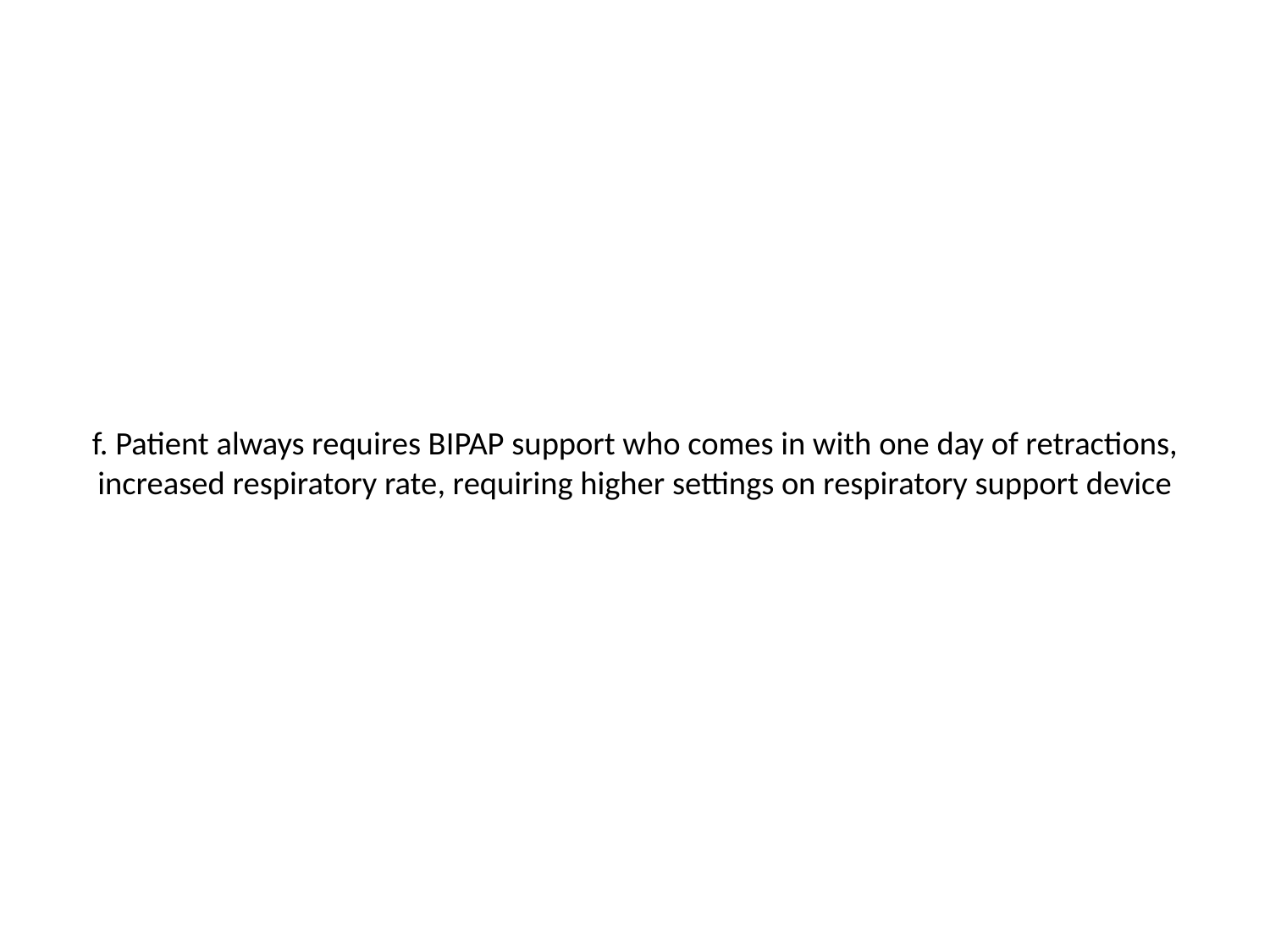

# f. Patient always requires BIPAP support who comes in with one day of retractions, increased respiratory rate, requiring higher settings on respiratory support device

## Slide 16
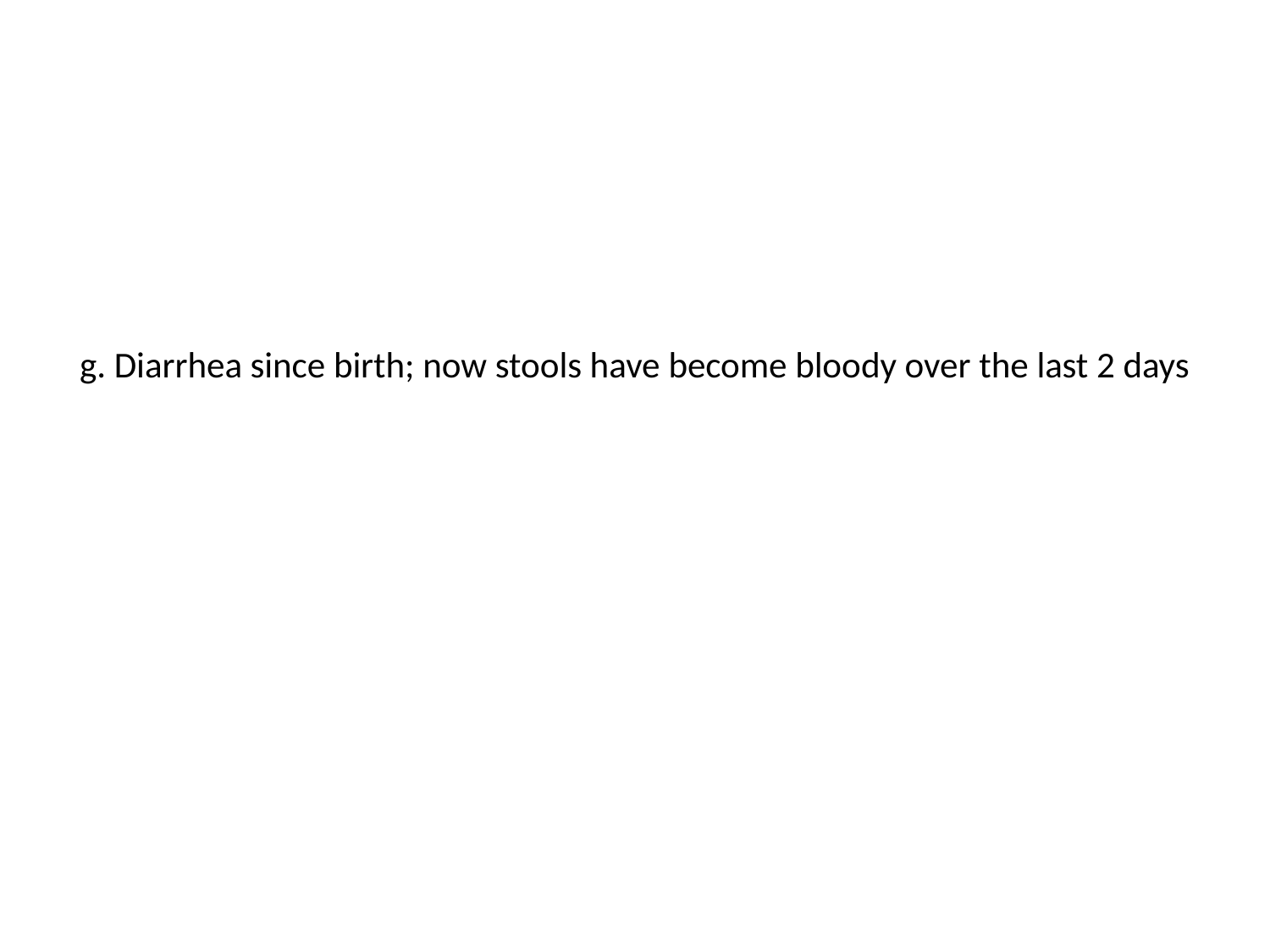

# g. Diarrhea since birth; now stools have become bloody over the last 2 days

## Slide 17
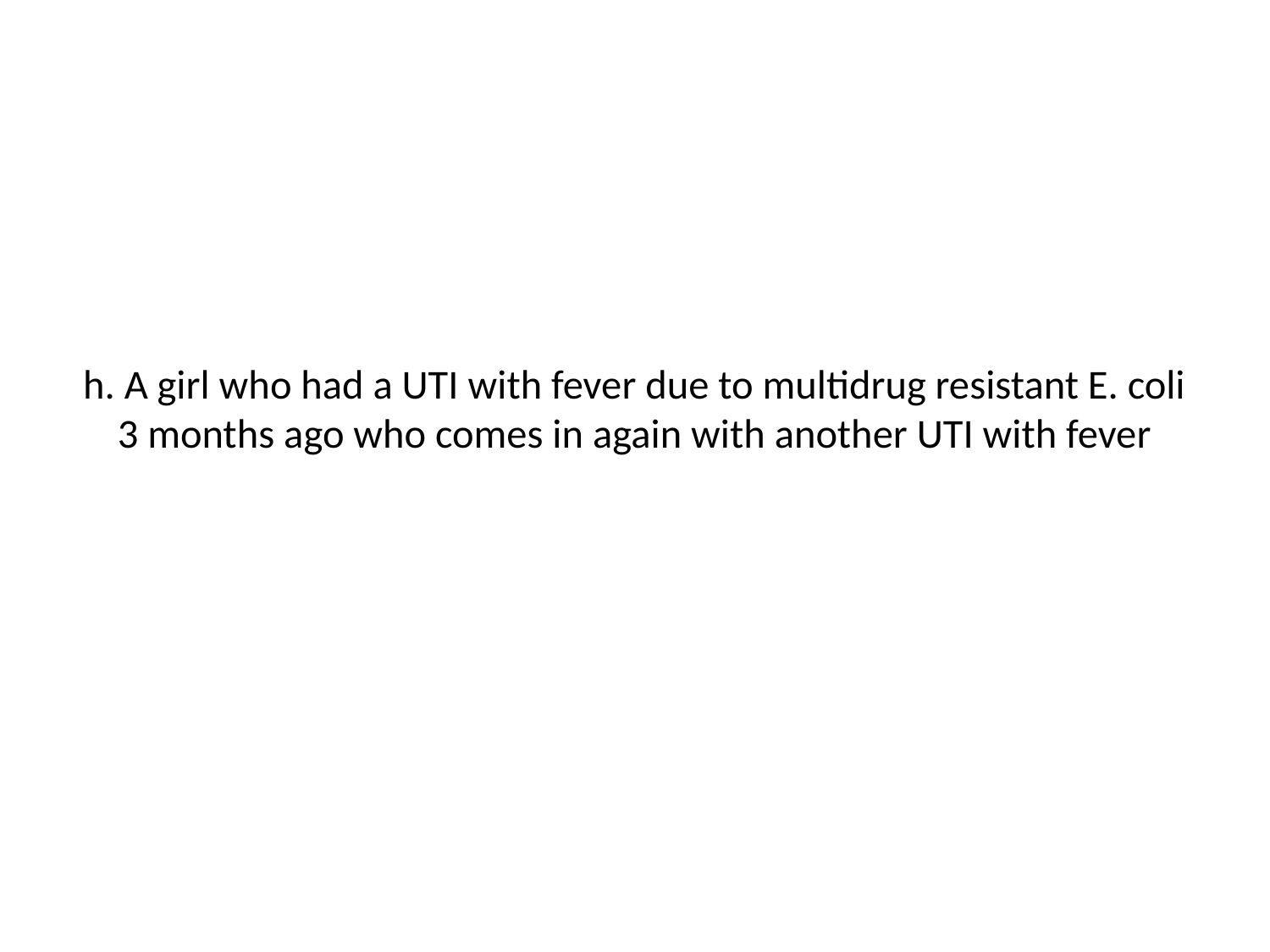

# h. A girl who had a UTI with fever due to multidrug resistant E. coli 3 months ago who comes in again with another UTI with fever

## Slide 18
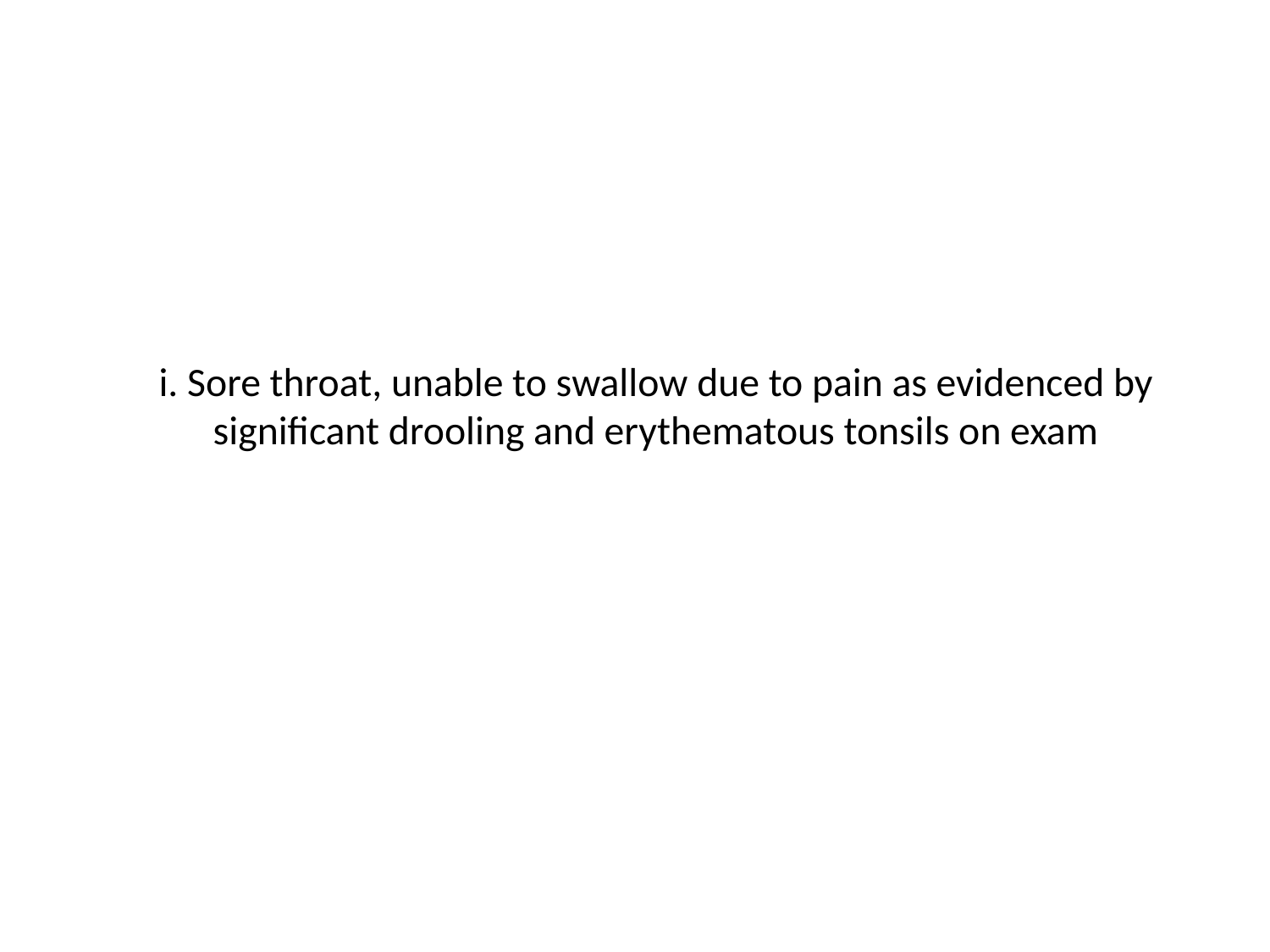

# i. Sore throat, unable to swallow due to pain as evidenced by significant drooling and erythematous tonsils on exam

## Slide 19
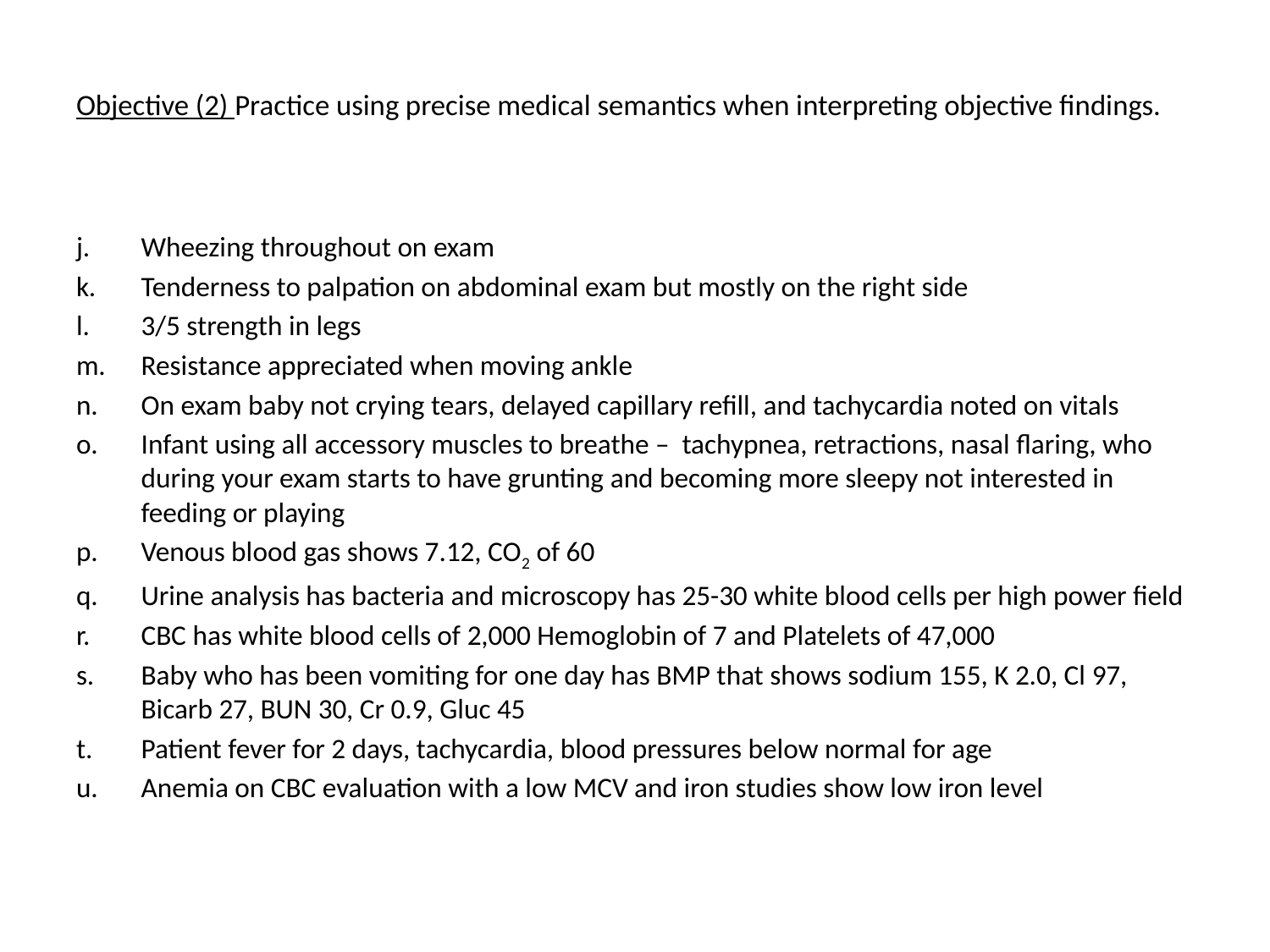

# Objective (2) Practice using precise medical semantics when interpreting objective findings.
j.	Wheezing throughout on exam
k.	Tenderness to palpation on abdominal exam but mostly on the right side
l.	3/5 strength in legs
m.	Resistance appreciated when moving ankle
n.	On exam baby not crying tears, delayed capillary refill, and tachycardia noted on vitals
o.	Infant using all accessory muscles to breathe – tachypnea, retractions, nasal flaring, who during your exam starts to have grunting and becoming more sleepy not interested in feeding or playing
p.	Venous blood gas shows 7.12, CO2 of 60
q.	Urine analysis has bacteria and microscopy has 25-30 white blood cells per high power field
r.	CBC has white blood cells of 2,000 Hemoglobin of 7 and Platelets of 47,000
s.	Baby who has been vomiting for one day has BMP that shows sodium 155, K 2.0, Cl 97, Bicarb 27, BUN 30, Cr 0.9, Gluc 45
t.	Patient fever for 2 days, tachycardia, blood pressures below normal for age
u.	Anemia on CBC evaluation with a low MCV and iron studies show low iron level

## Slide 20
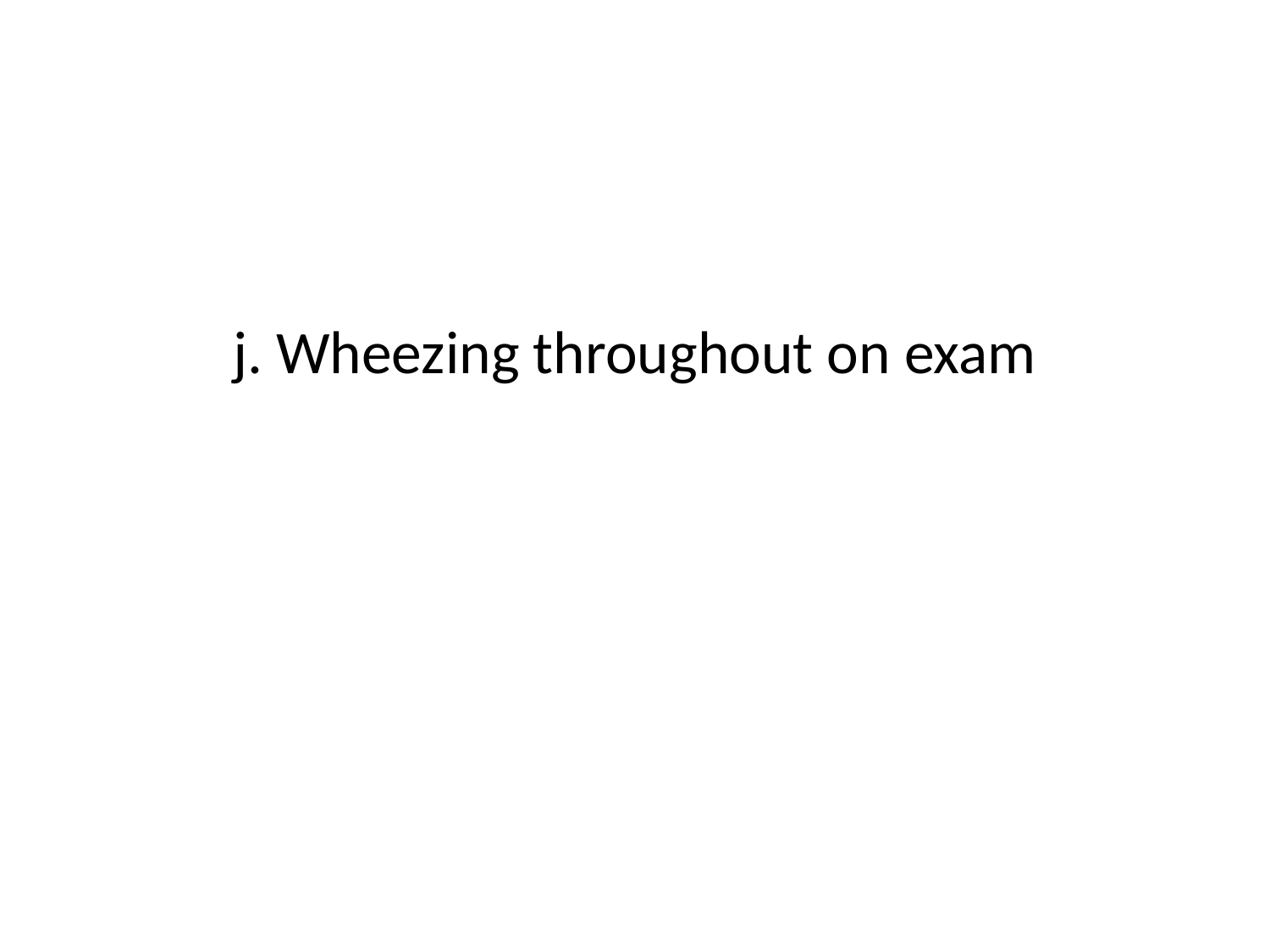

# j. Wheezing throughout on exam

## Slide 21
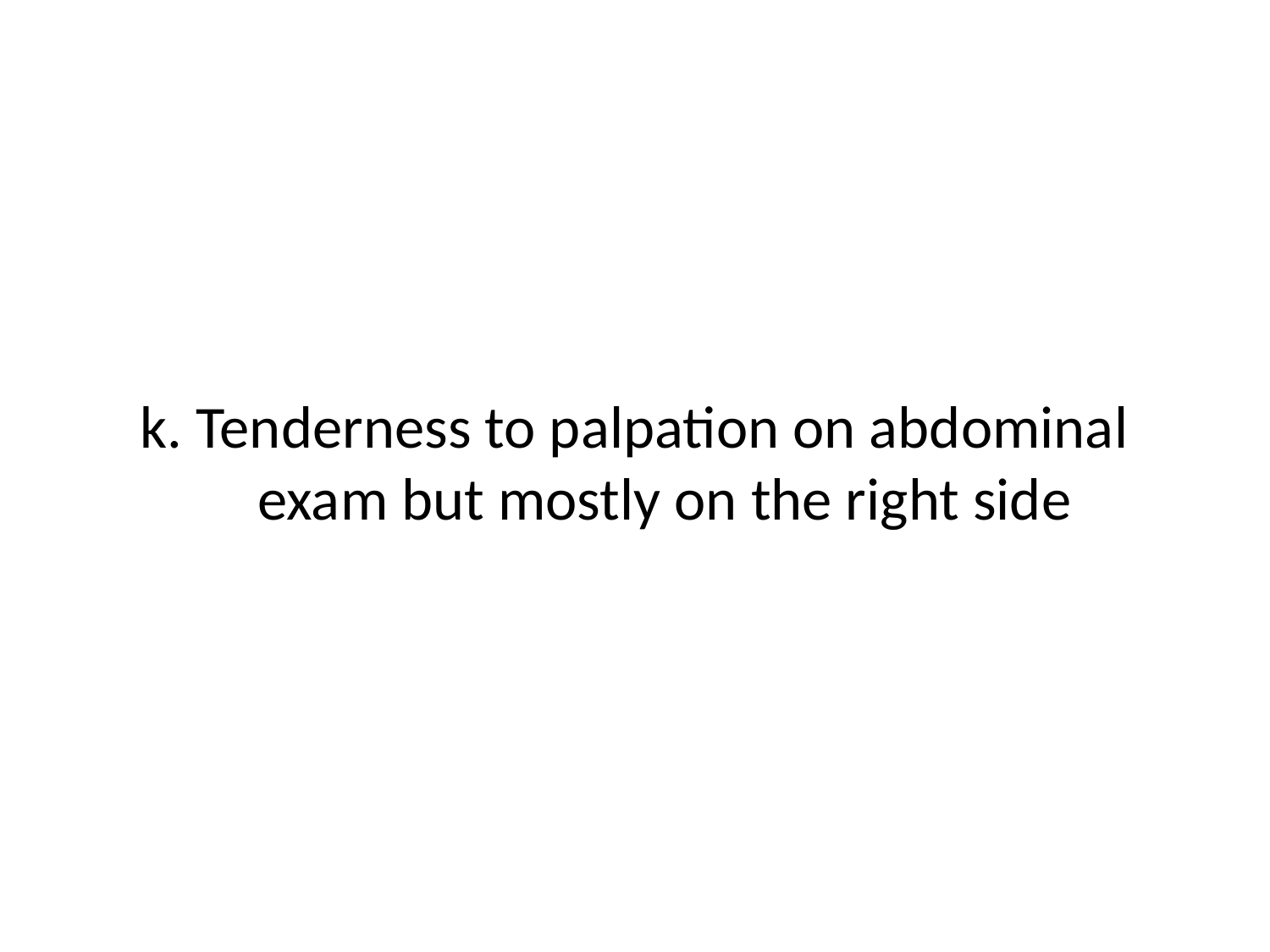

# k. Tenderness to palpation on abdominal exam but mostly on the right side

## Slide 22
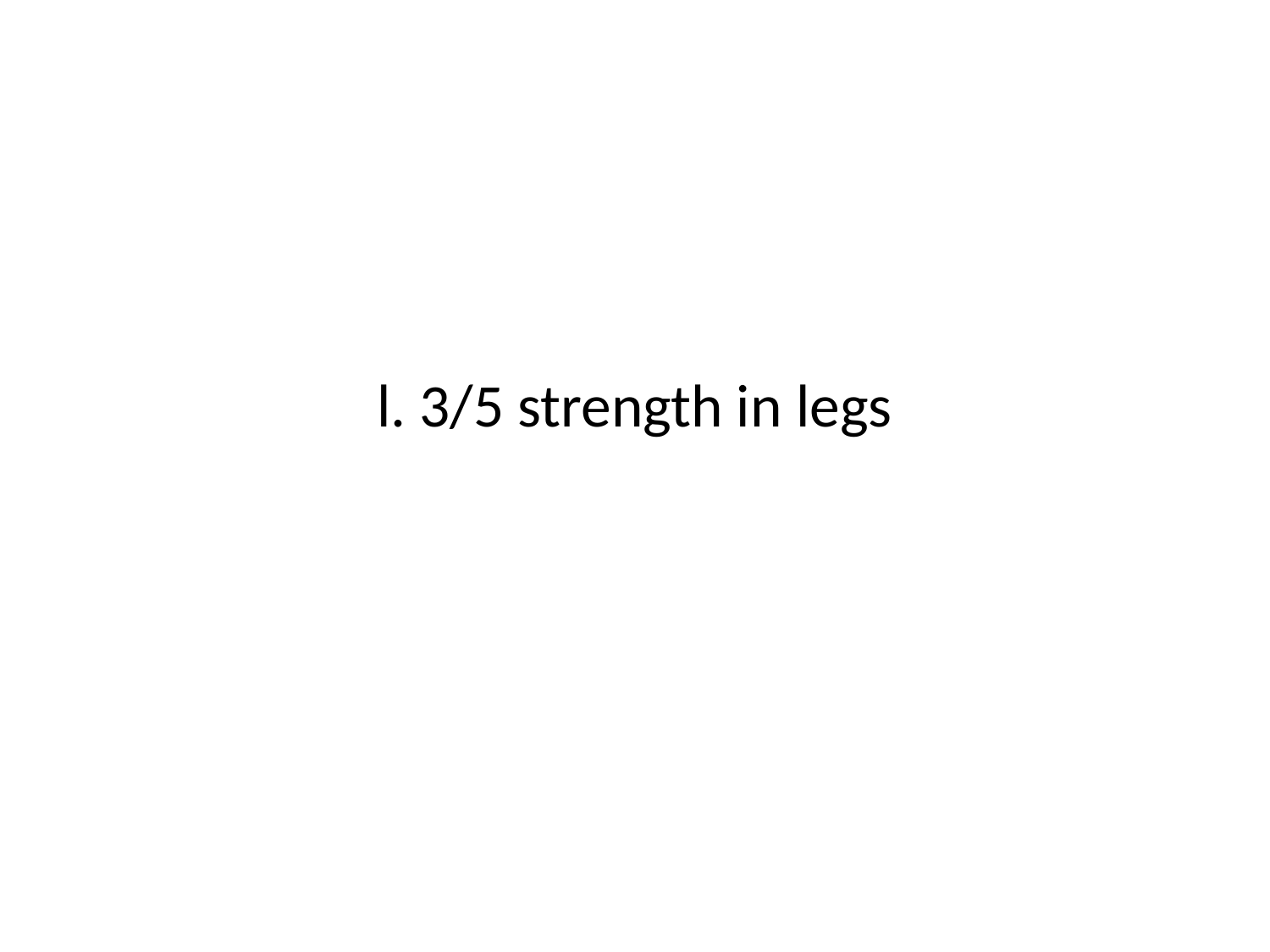

# l. 3/5 strength in legs

## Slide 23
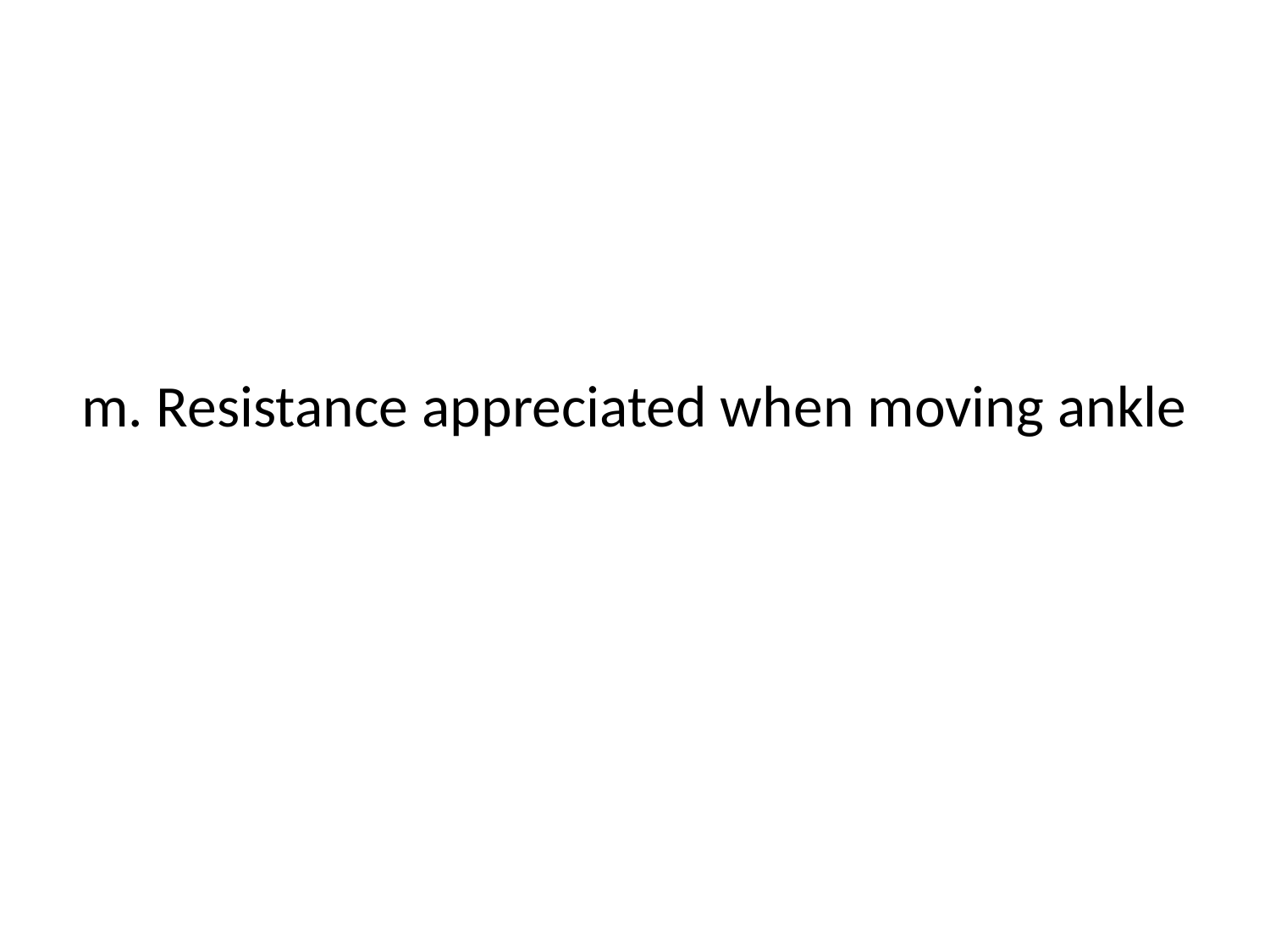

# m. Resistance appreciated when moving ankle

## Slide 24
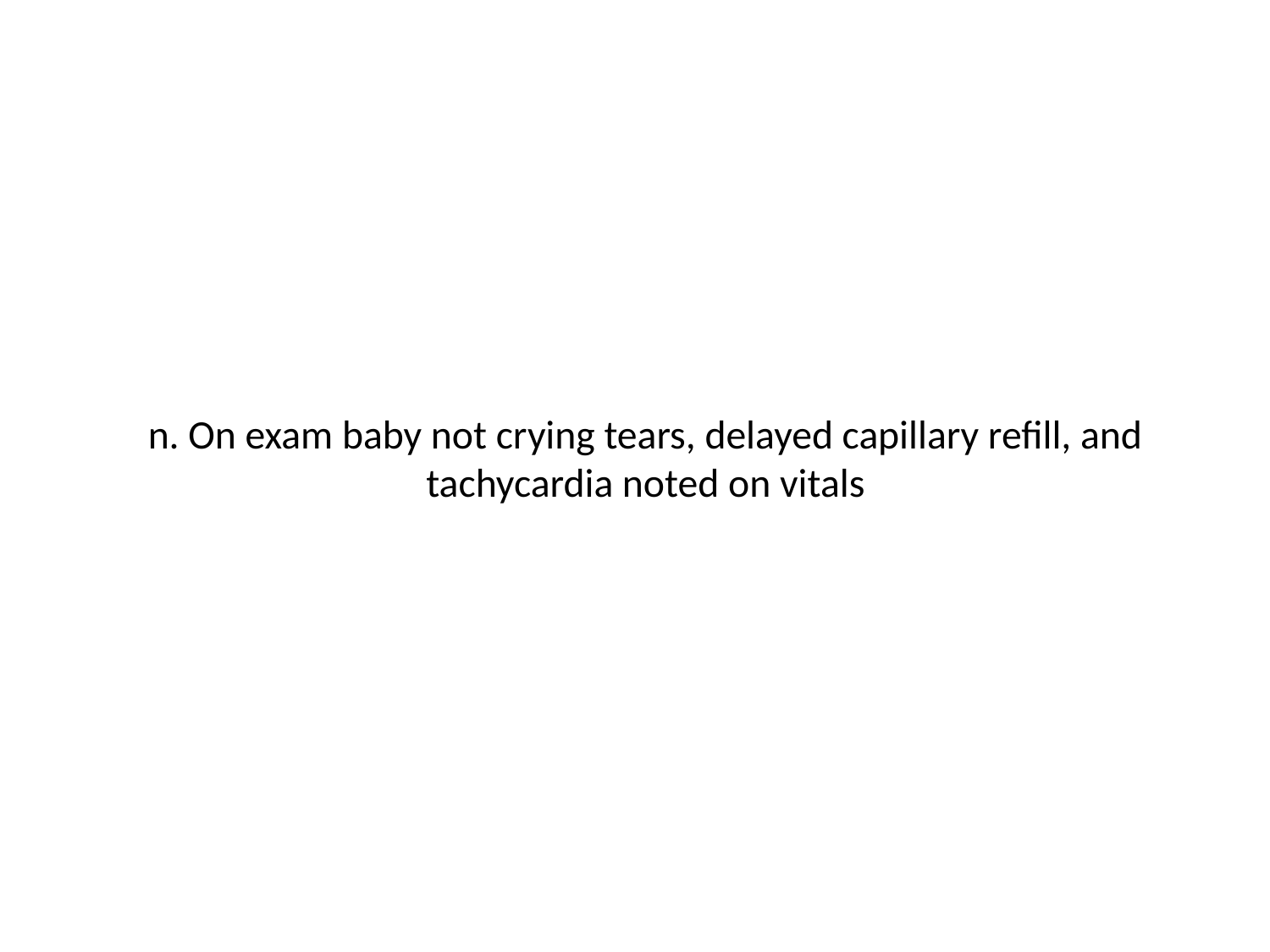

# n. On exam baby not crying tears, delayed capillary refill, and tachycardia noted on vitals

## Slide 25
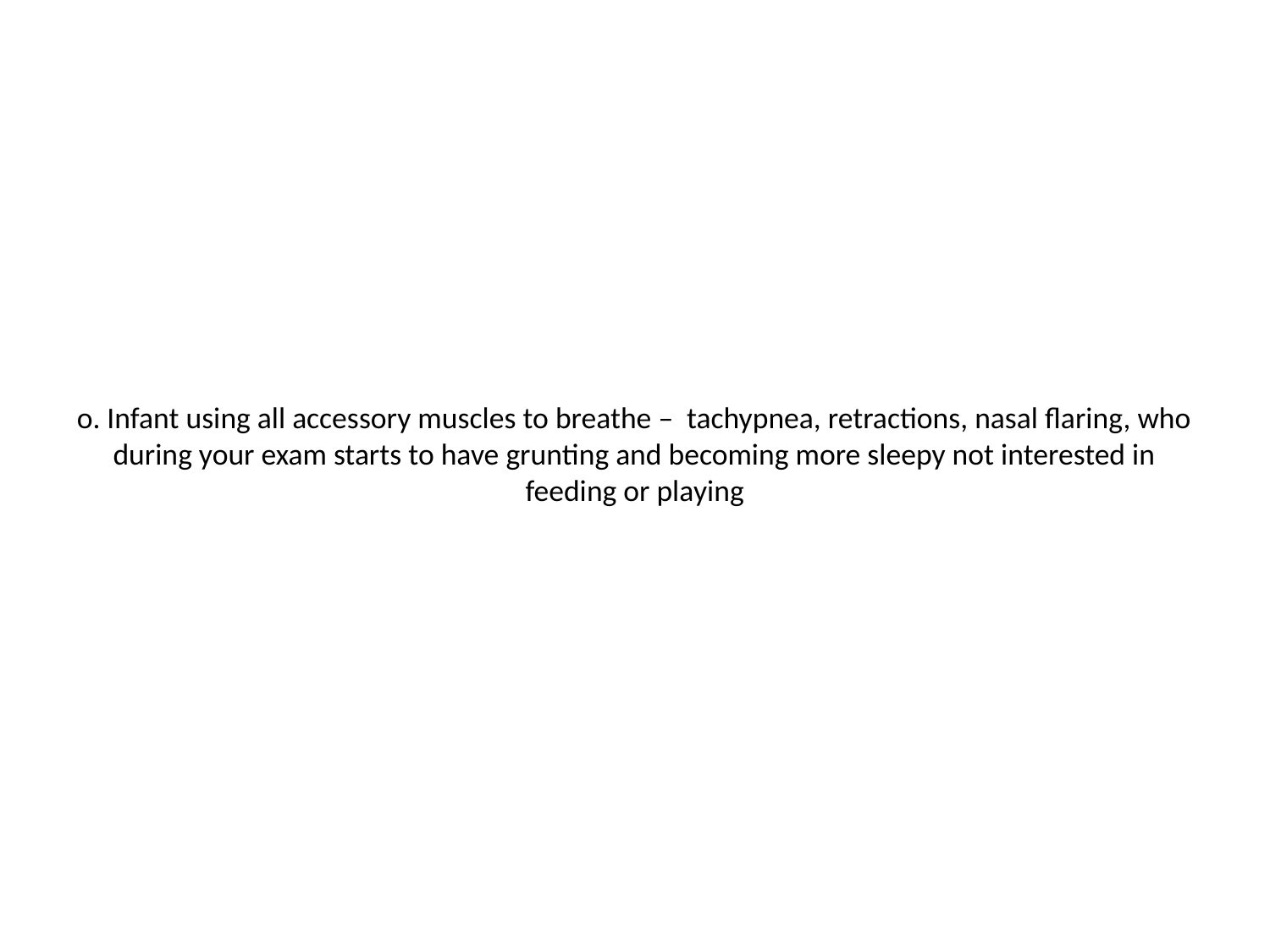

# o. Infant using all accessory muscles to breathe – tachypnea, retractions, nasal flaring, who during your exam starts to have grunting and becoming more sleepy not interested in feeding or playing

## Slide 26
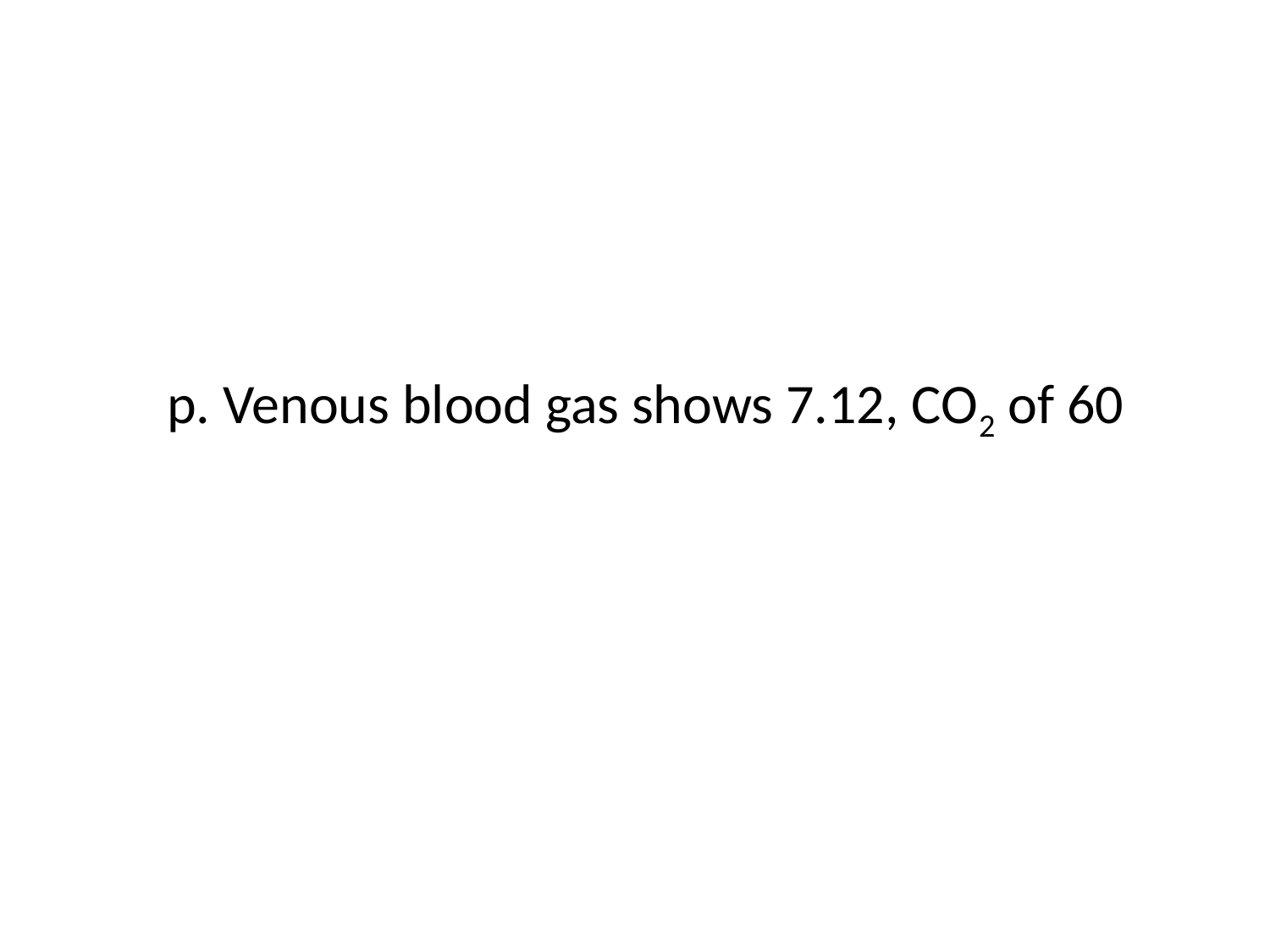

# p. Venous blood gas shows 7.12, CO2 of 60

## Slide 27
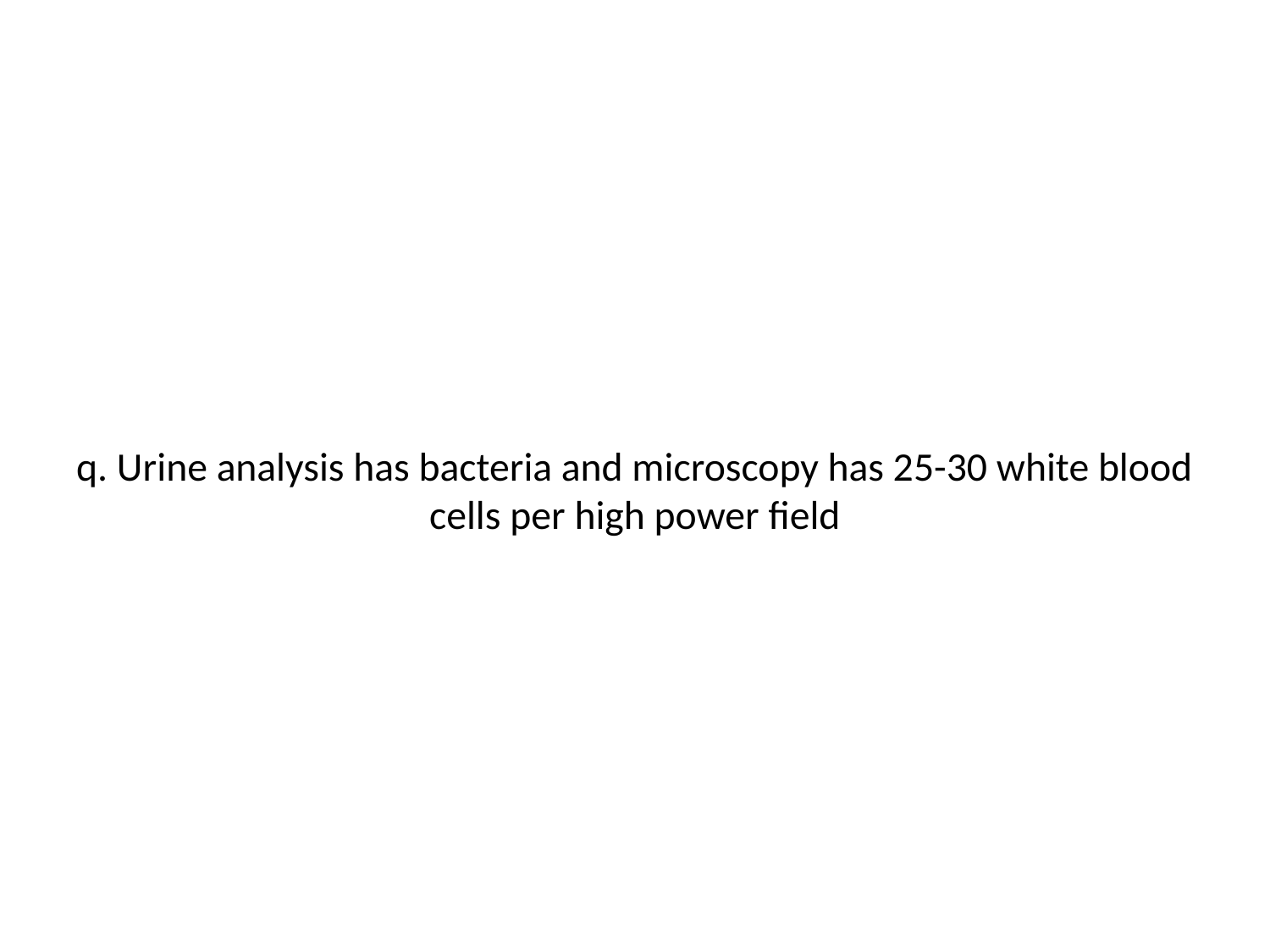

# q. Urine analysis has bacteria and microscopy has 25-30 white blood cells per high power field

## Slide 28
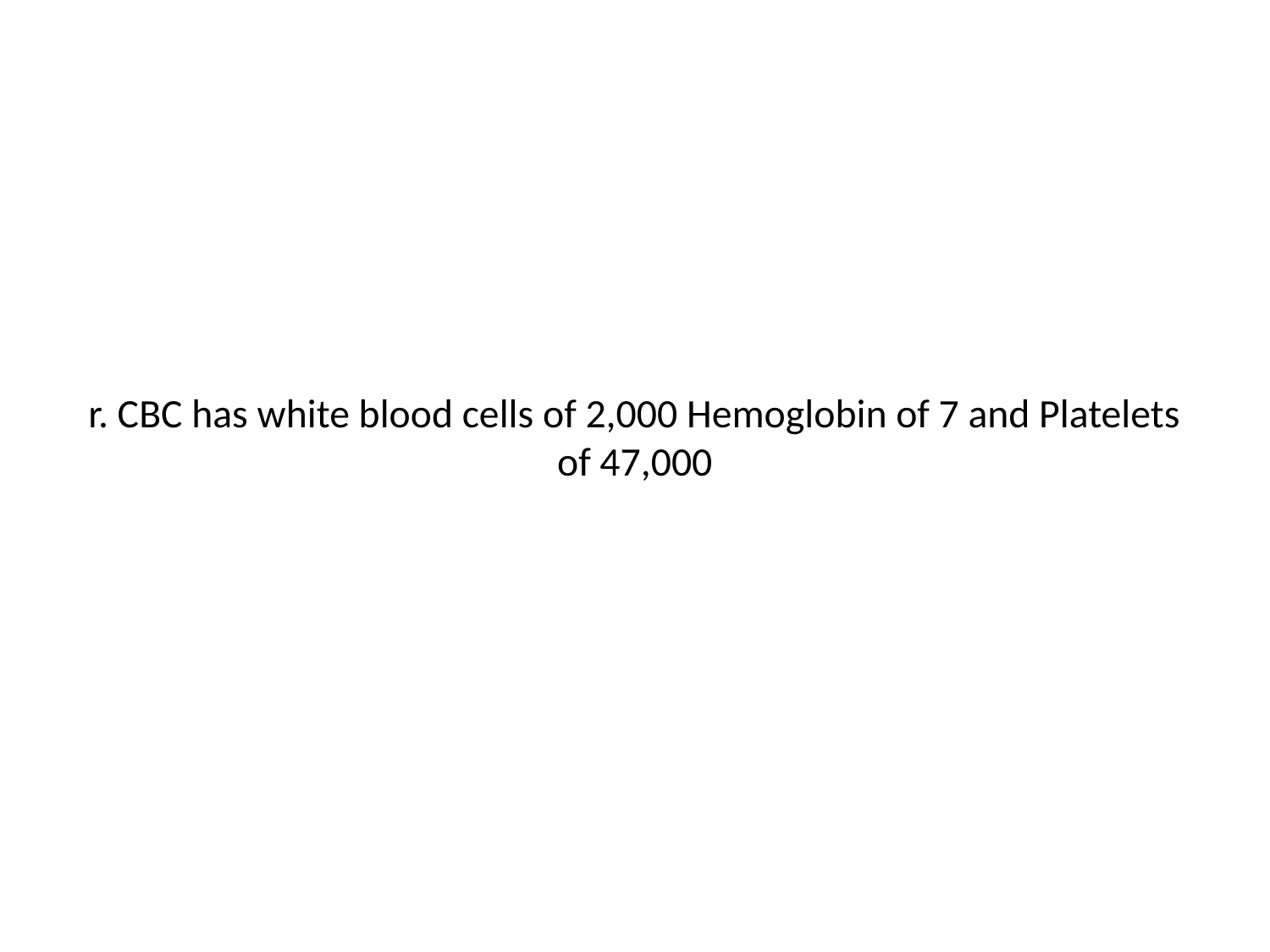

# r. CBC has white blood cells of 2,000 Hemoglobin of 7 and Platelets of 47,000

## Slide 29
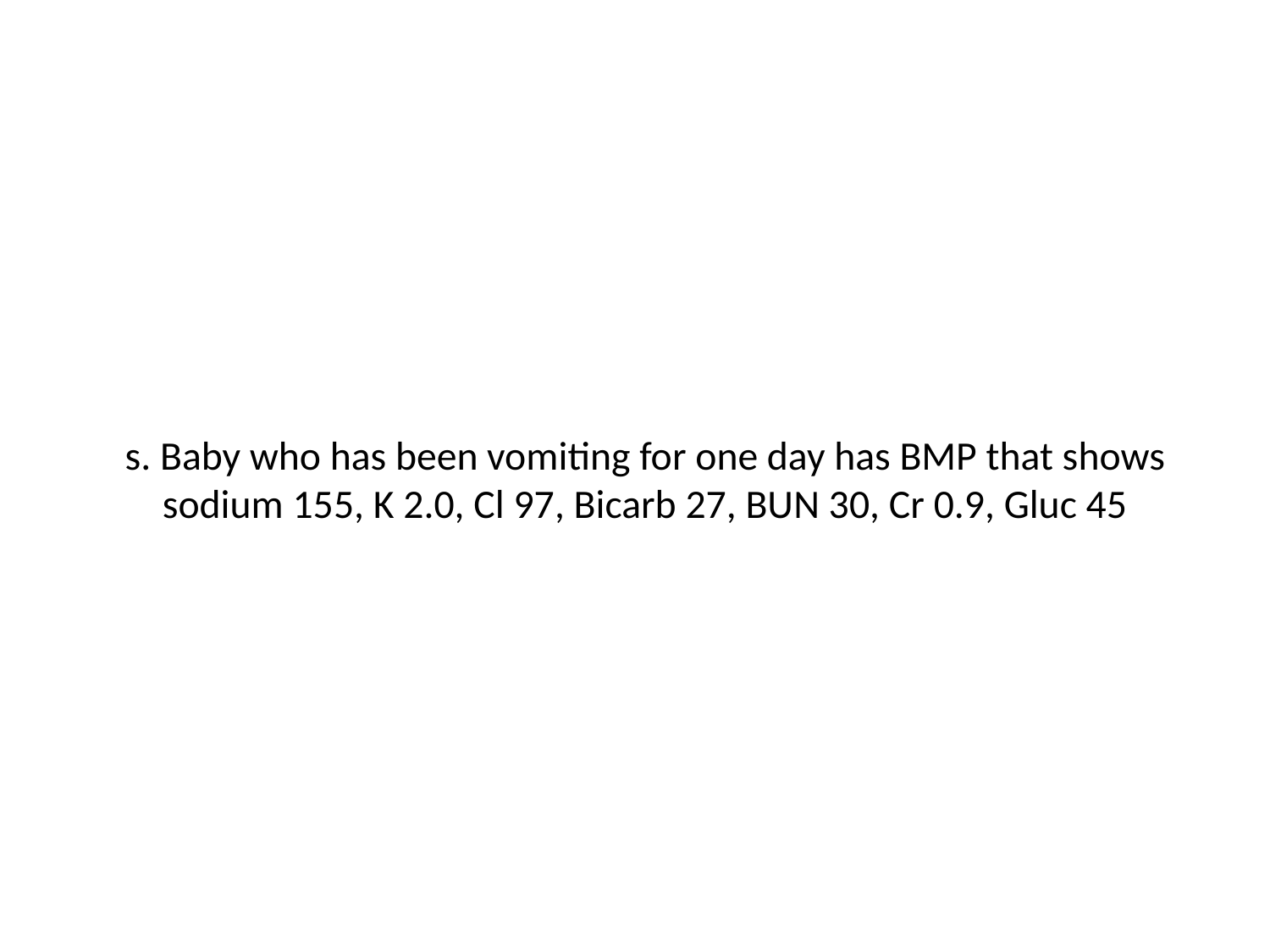

# s. Baby who has been vomiting for one day has BMP that shows sodium 155, K 2.0, Cl 97, Bicarb 27, BUN 30, Cr 0.9, Gluc 45

## Slide 30
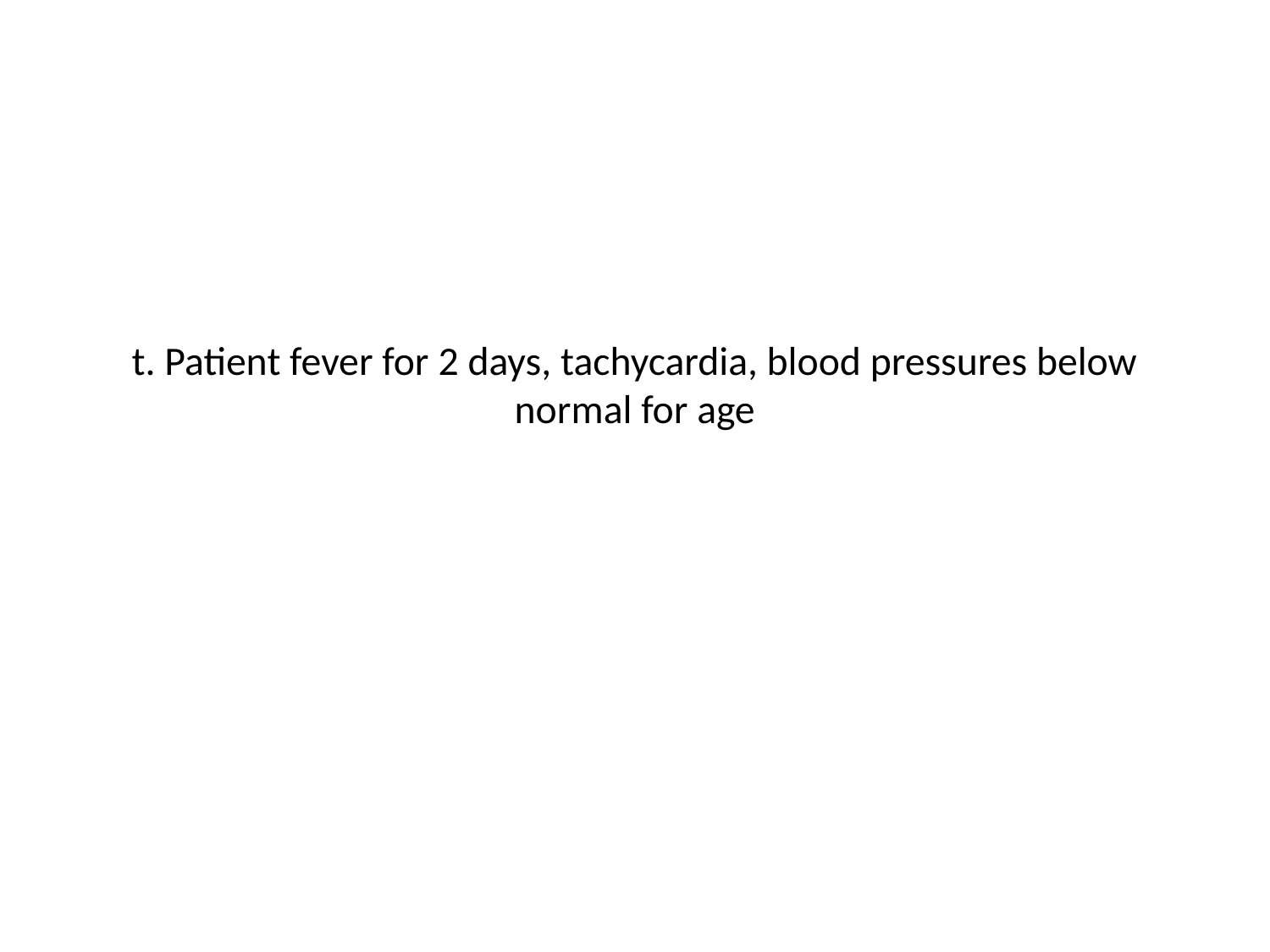

# t. Patient fever for 2 days, tachycardia, blood pressures below normal for age

## Slide 31
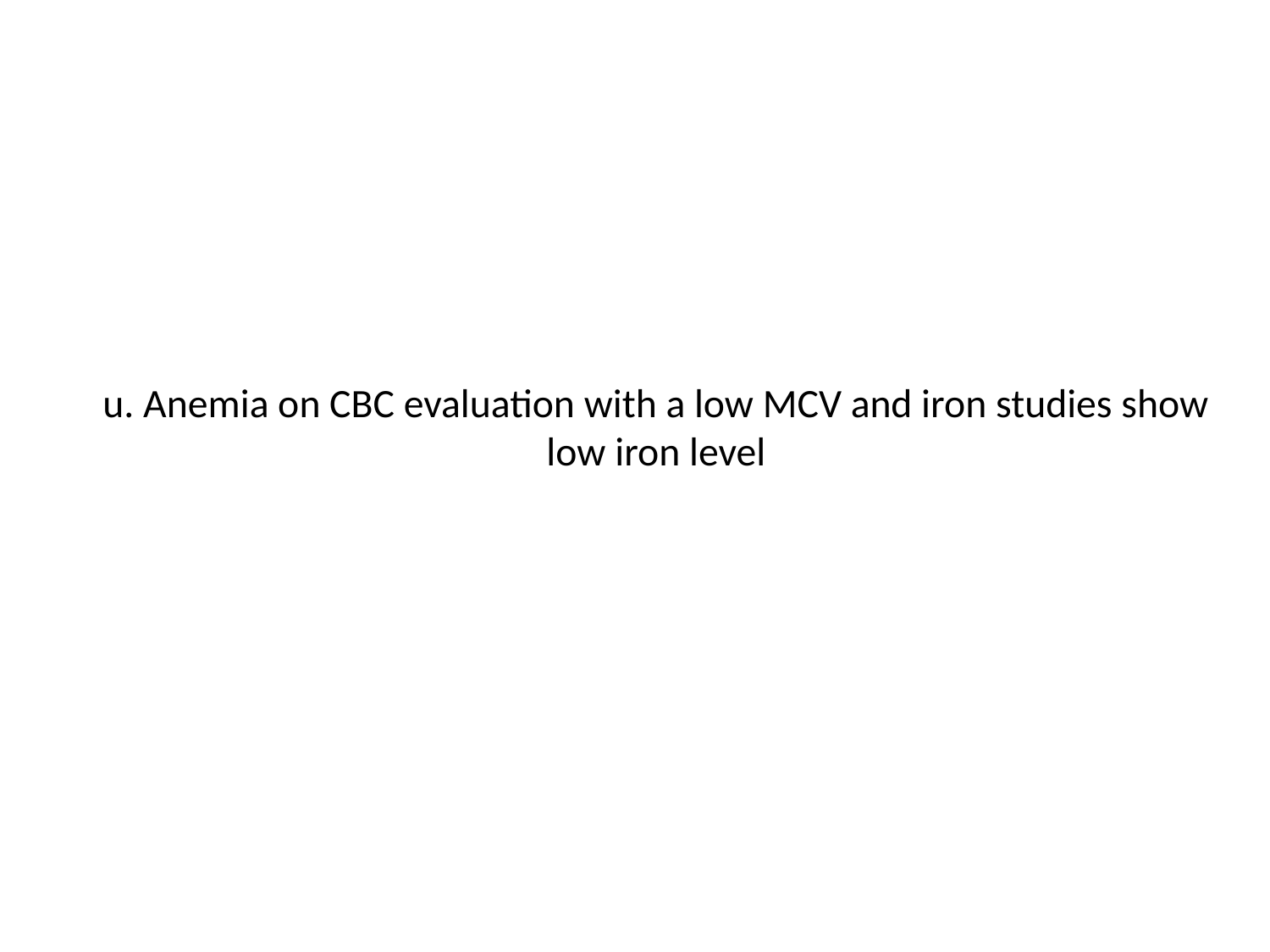

# u. Anemia on CBC evaluation with a low MCV and iron studies show low iron level

## Slide 32
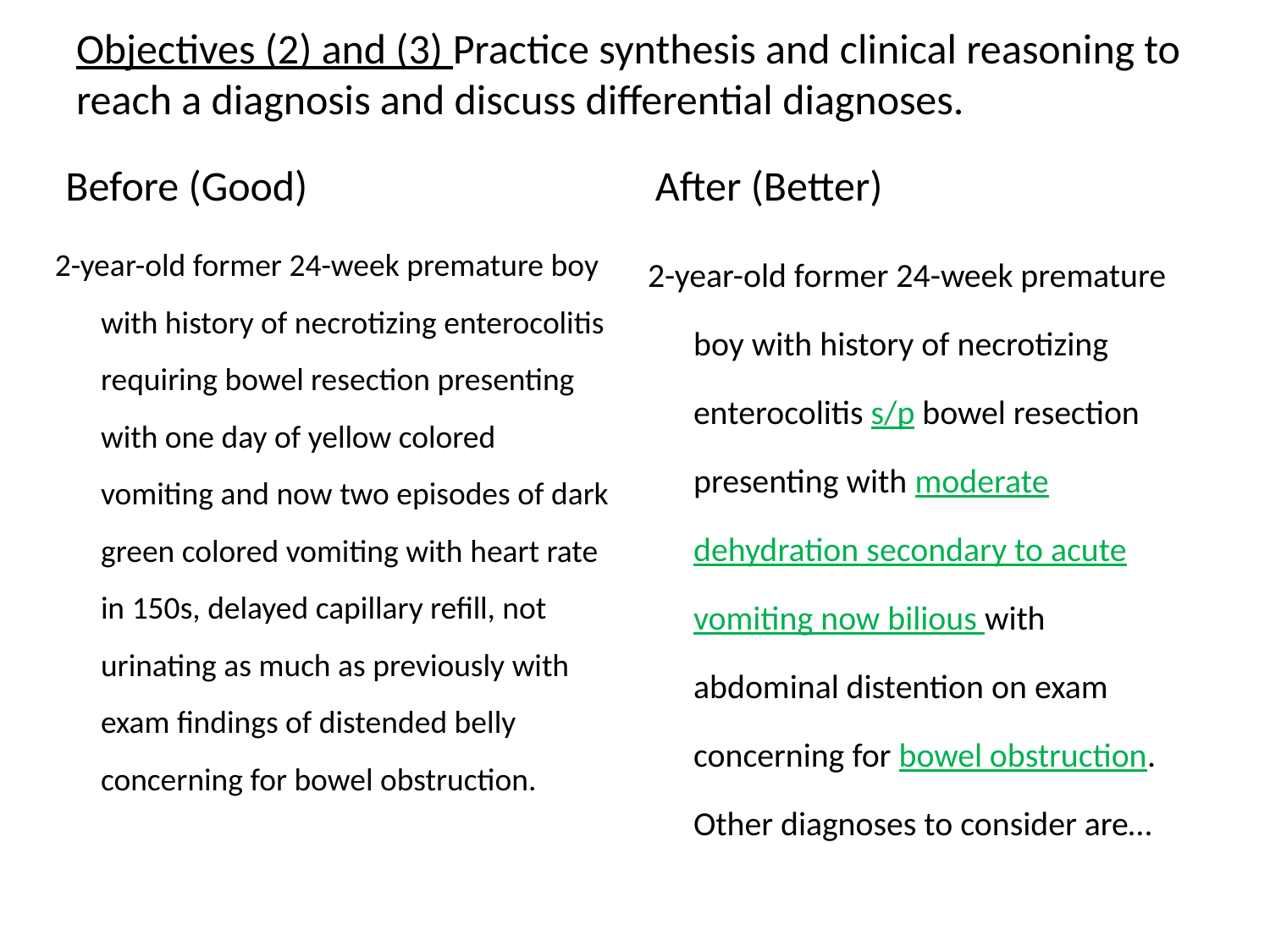

# Objectives (2) and (3) Practice synthesis and clinical reasoning to reach a diagnosis and discuss differential diagnoses.
Before (Good)
After (Better)
2-year-old former 24-week premature boy with history of necrotizing enterocolitis s/p bowel resection presenting with moderate dehydration secondary to acute vomiting now bilious with abdominal distention on exam concerning for bowel obstruction. Other diagnoses to consider are…
2-year-old former 24-week premature boy with history of necrotizing enterocolitis requiring bowel resection presenting with one day of yellow colored vomiting and now two episodes of dark green colored vomiting with heart rate in 150s, delayed capillary refill, not urinating as much as previously with exam findings of distended belly concerning for bowel obstruction.

## Slide 33
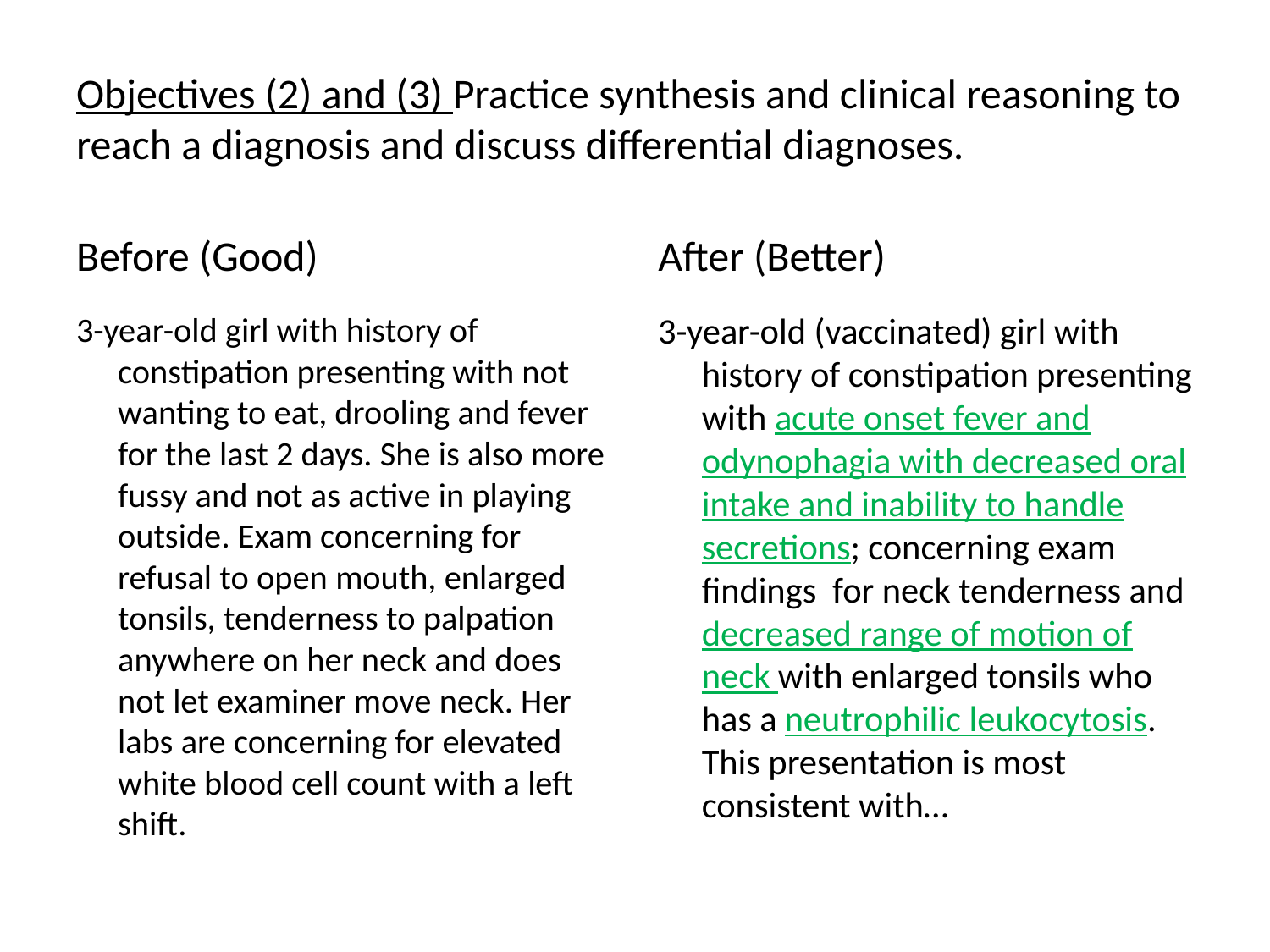

# Objectives (2) and (3) Practice synthesis and clinical reasoning to reach a diagnosis and discuss differential diagnoses.
Before (Good)
After (Better)
3-year-old girl with history of constipation presenting with not wanting to eat, drooling and fever for the last 2 days. She is also more fussy and not as active in playing outside. Exam concerning for refusal to open mouth, enlarged tonsils, tenderness to palpation anywhere on her neck and does not let examiner move neck. Her labs are concerning for elevated white blood cell count with a left shift.
3-year-old (vaccinated) girl with history of constipation presenting with acute onset fever and odynophagia with decreased oral intake and inability to handle secretions; concerning exam findings for neck tenderness and decreased range of motion of neck with enlarged tonsils who has a neutrophilic leukocytosis. This presentation is most consistent with…

## Slide 34
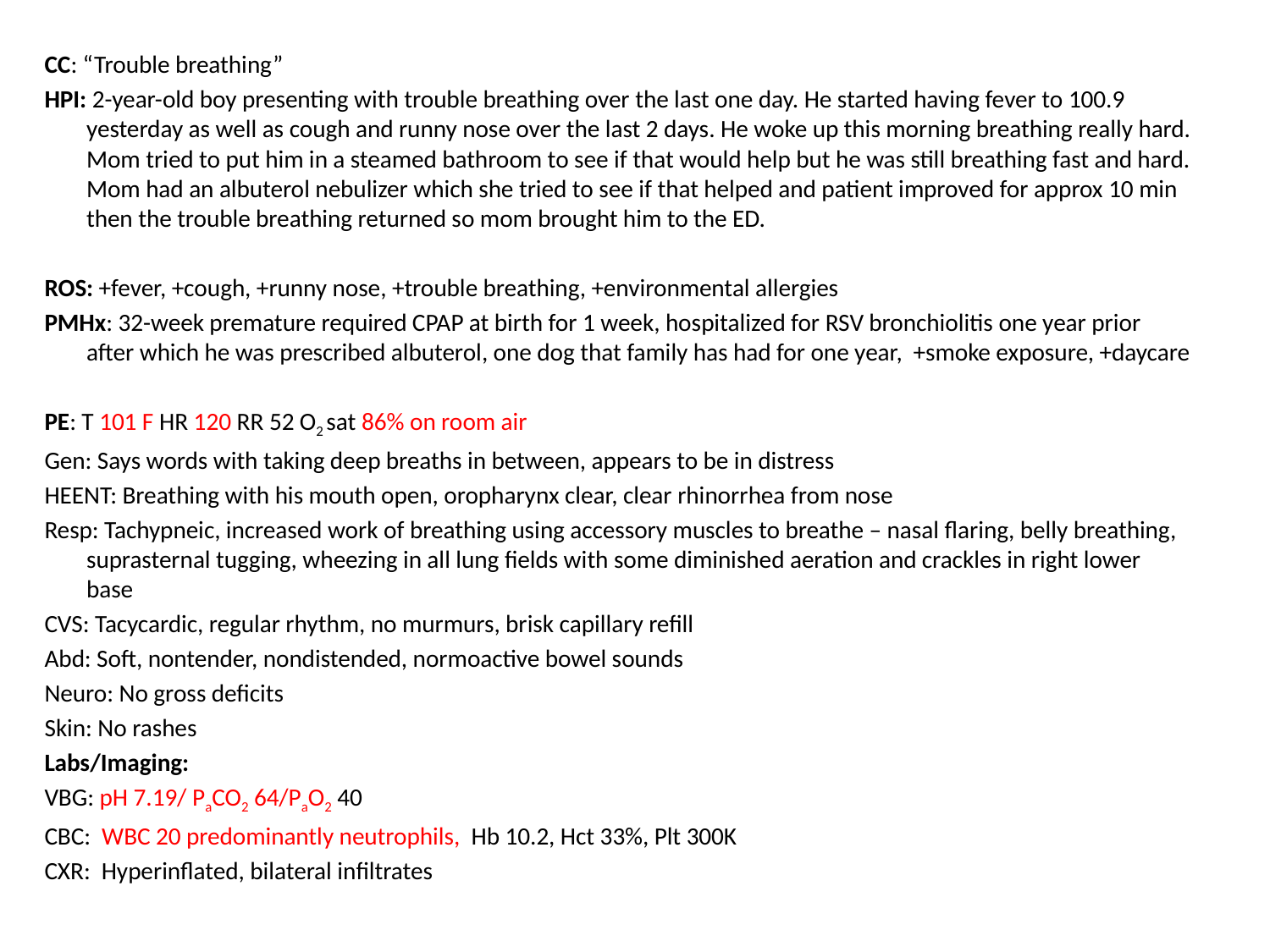

CC: “Trouble breathing”
HPI: 2-year-old boy presenting with trouble breathing over the last one day. He started having fever to 100.9 yesterday as well as cough and runny nose over the last 2 days. He woke up this morning breathing really hard. Mom tried to put him in a steamed bathroom to see if that would help but he was still breathing fast and hard. Mom had an albuterol nebulizer which she tried to see if that helped and patient improved for approx 10 min then the trouble breathing returned so mom brought him to the ED.
ROS: +fever, +cough, +runny nose, +trouble breathing, +environmental allergies
PMHx: 32-week premature required CPAP at birth for 1 week, hospitalized for RSV bronchiolitis one year prior after which he was prescribed albuterol, one dog that family has had for one year, +smoke exposure, +daycare
PE: T 101 F HR 120 RR 52 O2 sat 86% on room air
Gen: Says words with taking deep breaths in between, appears to be in distress
HEENT: Breathing with his mouth open, oropharynx clear, clear rhinorrhea from nose
Resp: Tachypneic, increased work of breathing using accessory muscles to breathe – nasal flaring, belly breathing, suprasternal tugging, wheezing in all lung fields with some diminished aeration and crackles in right lower base
CVS: Tacycardic, regular rhythm, no murmurs, brisk capillary refill
Abd: Soft, nontender, nondistended, normoactive bowel sounds
Neuro: No gross deficits
Skin: No rashes
Labs/Imaging:
VBG: pH 7.19/ PaCO2 64/PaO2 40
CBC: WBC 20 predominantly neutrophils, Hb 10.2, Hct 33%, Plt 300K
CXR: Hyperinflated, bilateral infiltrates

## Slide 35
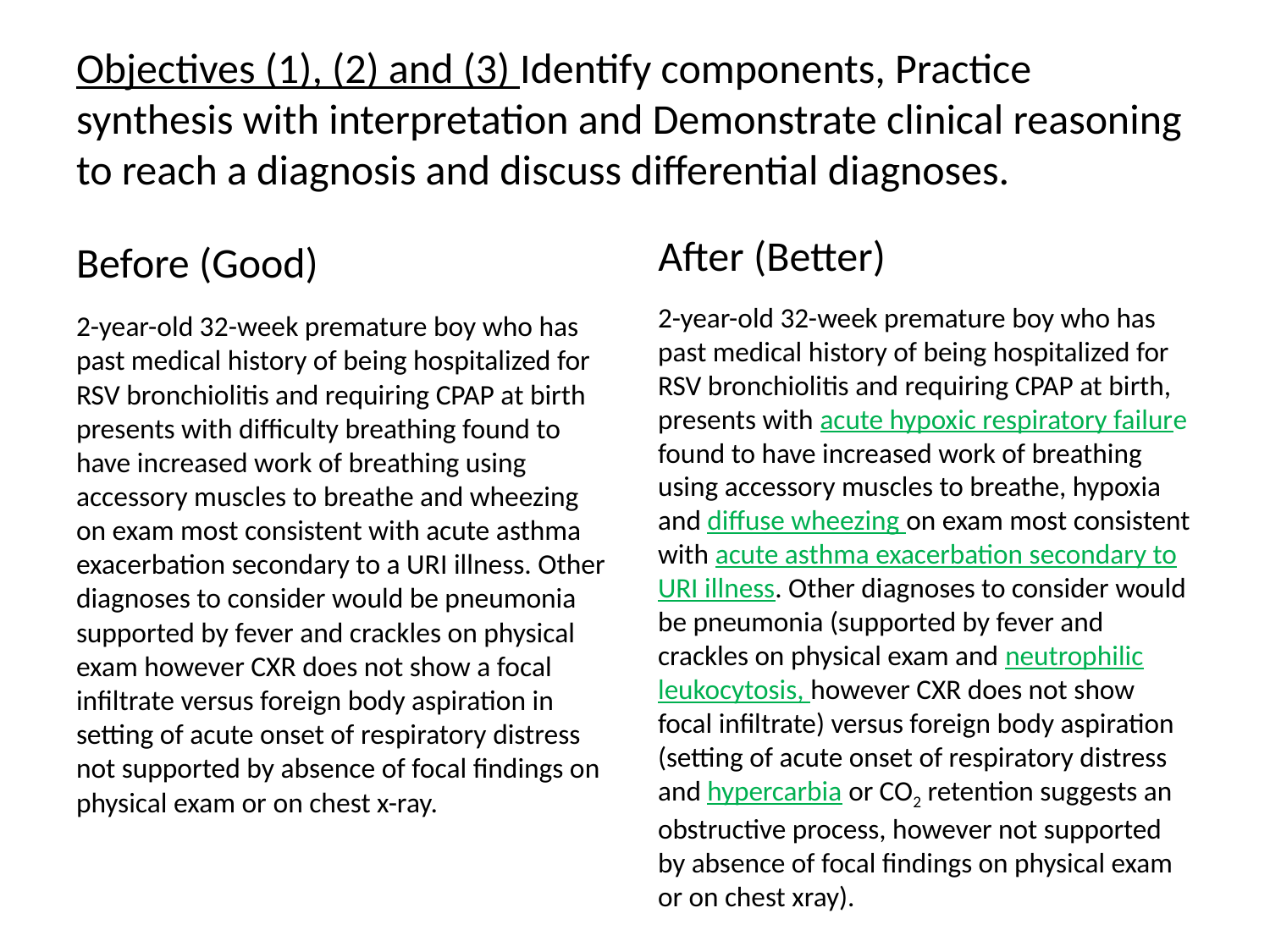

# Objectives (1), (2) and (3) Identify components, Practice synthesis with interpretation and Demonstrate clinical reasoning to reach a diagnosis and discuss differential diagnoses.
After (Better)
Before (Good)
2-year-old 32-week premature boy who has past medical history of being hospitalized for RSV bronchiolitis and requiring CPAP at birth, presents with acute hypoxic respiratory failure found to have increased work of breathing using accessory muscles to breathe, hypoxia and diffuse wheezing on exam most consistent with acute asthma exacerbation secondary to URI illness. Other diagnoses to consider would be pneumonia (supported by fever and crackles on physical exam and neutrophilic leukocytosis, however CXR does not show focal infiltrate) versus foreign body aspiration (setting of acute onset of respiratory distress and hypercarbia or CO2 retention suggests an obstructive process, however not supported by absence of focal findings on physical exam or on chest xray).
2-year-old 32-week premature boy who has past medical history of being hospitalized for RSV bronchiolitis and requiring CPAP at birth presents with difficulty breathing found to have increased work of breathing using accessory muscles to breathe and wheezing on exam most consistent with acute asthma exacerbation secondary to a URI illness. Other diagnoses to consider would be pneumonia supported by fever and crackles on physical exam however CXR does not show a focal infiltrate versus foreign body aspiration in setting of acute onset of respiratory distress not supported by absence of focal findings on physical exam or on chest x-ray.

## Slide 36
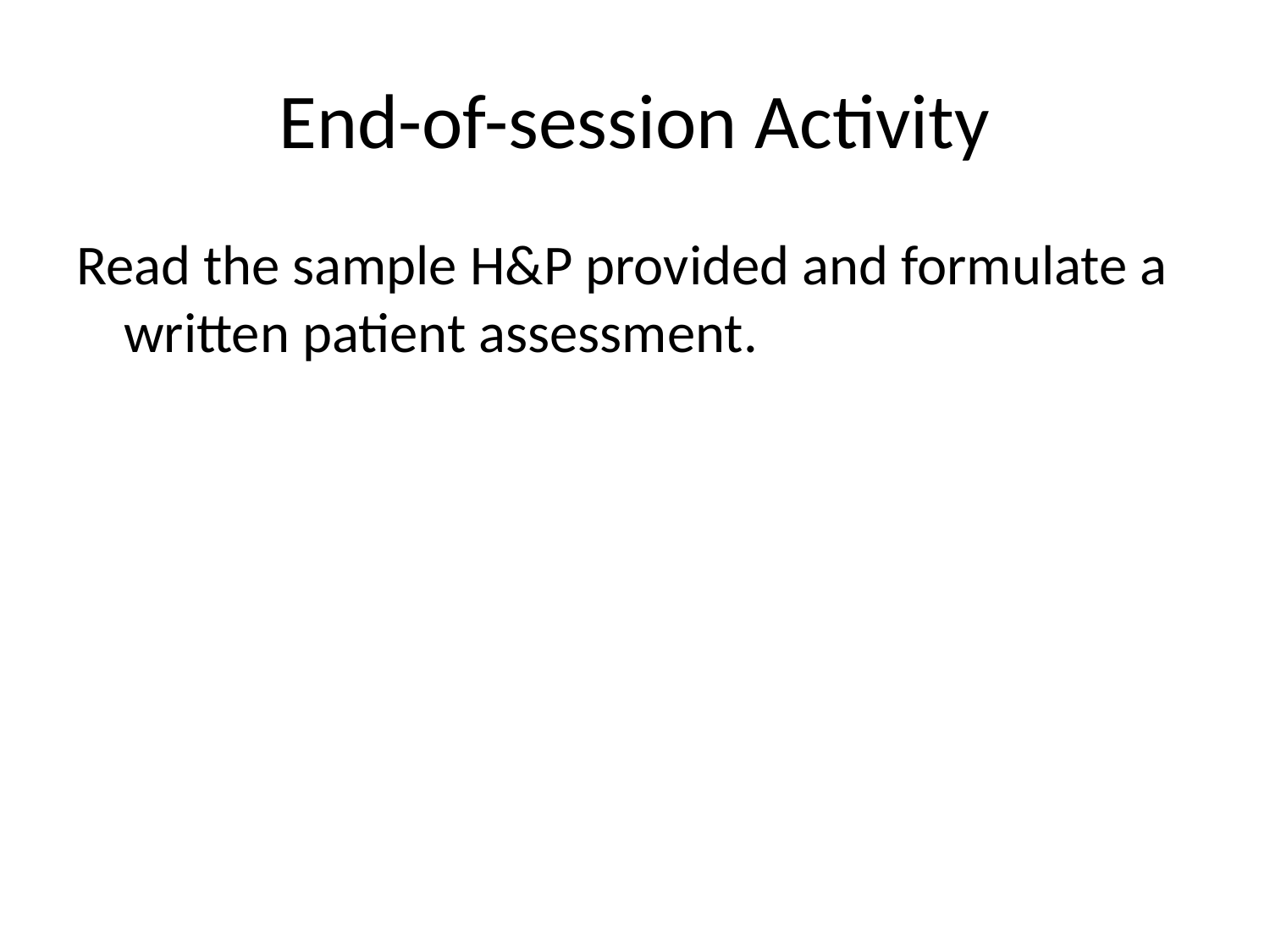

# End-of-session Activity
Read the sample H&P provided and formulate a written patient assessment.

## Slide 37
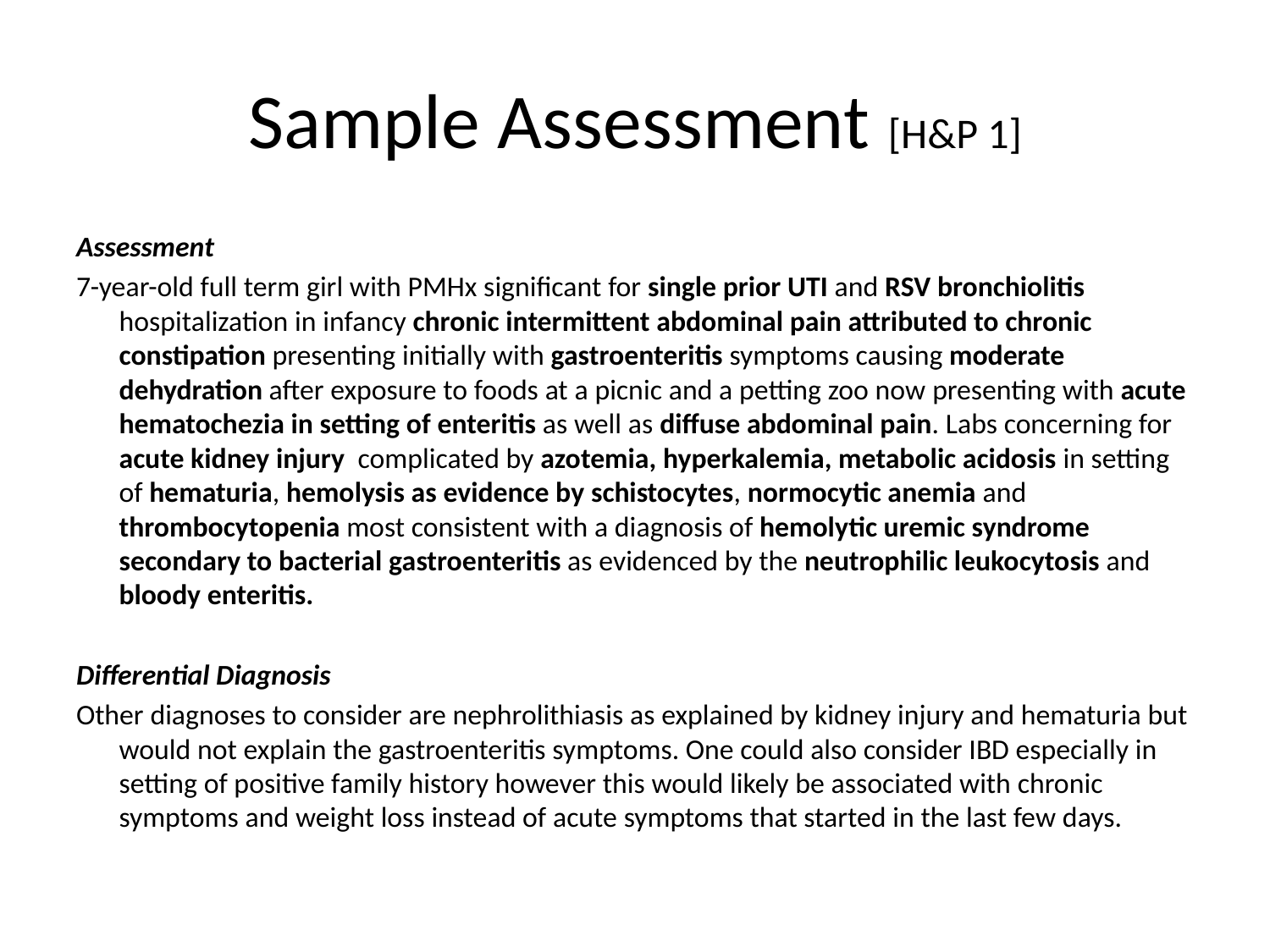

# Sample Assessment [H&P 1]
Assessment
7-year-old full term girl with PMHx significant for single prior UTI and RSV bronchiolitis hospitalization in infancy chronic intermittent abdominal pain attributed to chronic constipation presenting initially with gastroenteritis symptoms causing moderate dehydration after exposure to foods at a picnic and a petting zoo now presenting with acute hematochezia in setting of enteritis as well as diffuse abdominal pain. Labs concerning for acute kidney injury complicated by azotemia, hyperkalemia, metabolic acidosis in setting of hematuria, hemolysis as evidence by schistocytes, normocytic anemia and thrombocytopenia most consistent with a diagnosis of hemolytic uremic syndrome secondary to bacterial gastroenteritis as evidenced by the neutrophilic leukocytosis and bloody enteritis.
Differential Diagnosis
Other diagnoses to consider are nephrolithiasis as explained by kidney injury and hematuria but would not explain the gastroenteritis symptoms. One could also consider IBD especially in setting of positive family history however this would likely be associated with chronic symptoms and weight loss instead of acute symptoms that started in the last few days.

## Slide 38
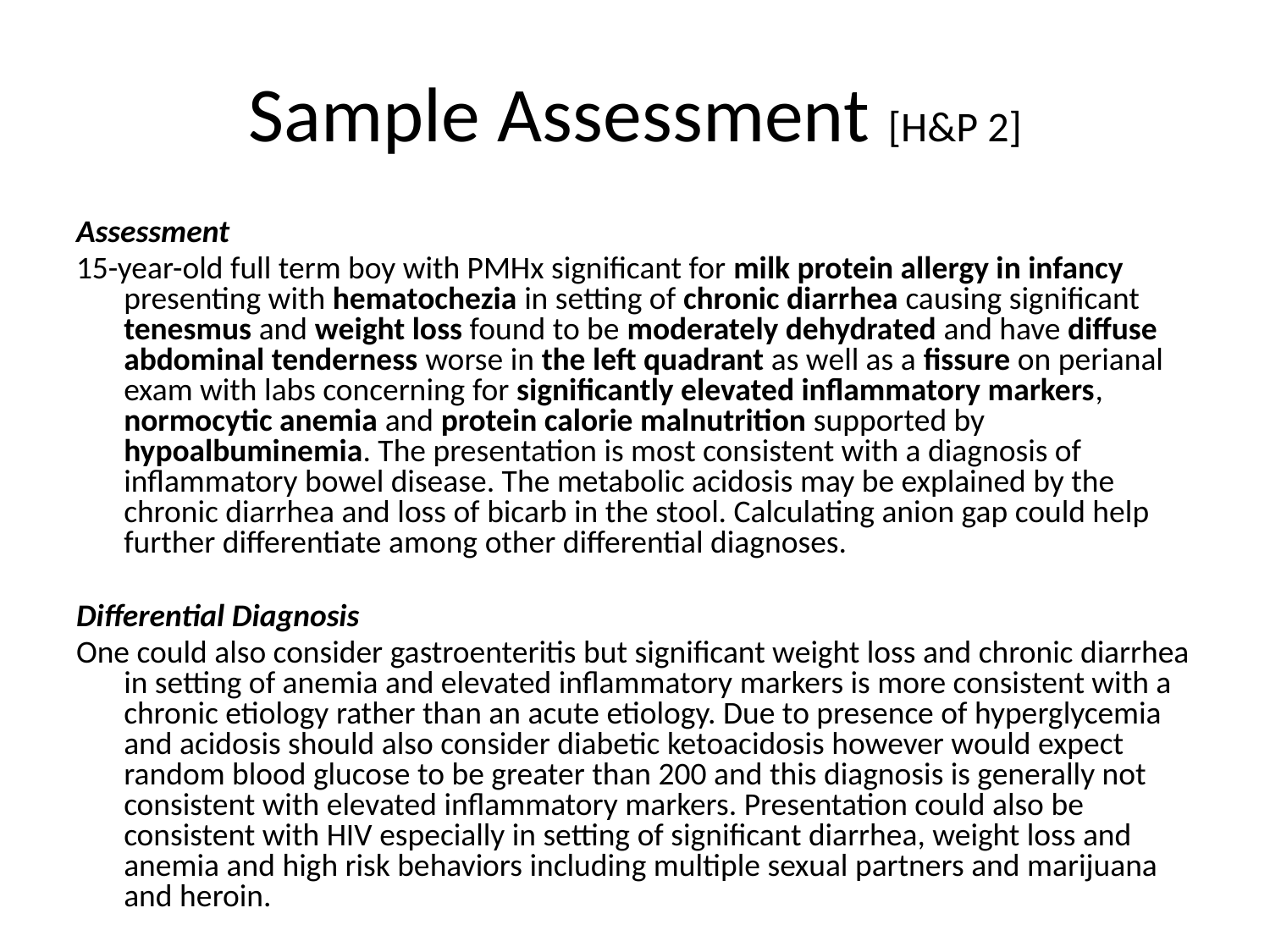

# Sample Assessment [H&P 2]
Assessment
15-year-old full term boy with PMHx significant for milk protein allergy in infancy presenting with hematochezia in setting of chronic diarrhea causing significant tenesmus and weight loss found to be moderately dehydrated and have diffuse abdominal tenderness worse in the left quadrant as well as a fissure on perianal exam with labs concerning for significantly elevated inflammatory markers, normocytic anemia and protein calorie malnutrition supported by hypoalbuminemia. The presentation is most consistent with a diagnosis of inflammatory bowel disease. The metabolic acidosis may be explained by the chronic diarrhea and loss of bicarb in the stool. Calculating anion gap could help further differentiate among other differential diagnoses.
Differential Diagnosis
One could also consider gastroenteritis but significant weight loss and chronic diarrhea in setting of anemia and elevated inflammatory markers is more consistent with a chronic etiology rather than an acute etiology. Due to presence of hyperglycemia and acidosis should also consider diabetic ketoacidosis however would expect random blood glucose to be greater than 200 and this diagnosis is generally not consistent with elevated inflammatory markers. Presentation could also be consistent with HIV especially in setting of significant diarrhea, weight loss and anemia and high risk behaviors including multiple sexual partners and marijuana and heroin.

## Slide 39
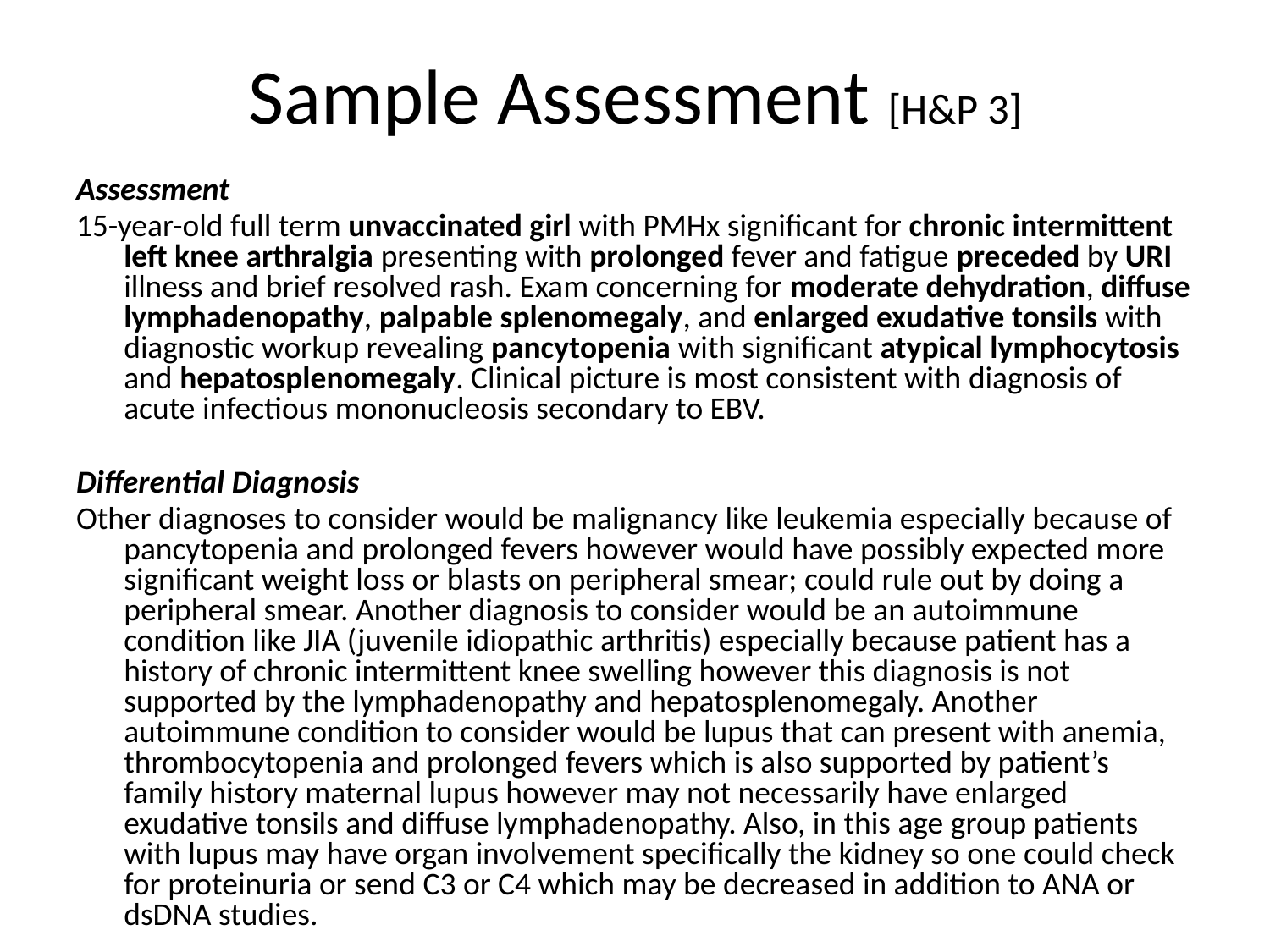

# Sample Assessment [H&P 3]
Assessment
15-year-old full term unvaccinated girl with PMHx significant for chronic intermittent left knee arthralgia presenting with prolonged fever and fatigue preceded by URI illness and brief resolved rash. Exam concerning for moderate dehydration, diffuse lymphadenopathy, palpable splenomegaly, and enlarged exudative tonsils with diagnostic workup revealing pancytopenia with significant atypical lymphocytosis and hepatosplenomegaly. Clinical picture is most consistent with diagnosis of acute infectious mononucleosis secondary to EBV.
Differential Diagnosis
Other diagnoses to consider would be malignancy like leukemia especially because of pancytopenia and prolonged fevers however would have possibly expected more significant weight loss or blasts on peripheral smear; could rule out by doing a peripheral smear. Another diagnosis to consider would be an autoimmune condition like JIA (juvenile idiopathic arthritis) especially because patient has a history of chronic intermittent knee swelling however this diagnosis is not supported by the lymphadenopathy and hepatosplenomegaly. Another autoimmune condition to consider would be lupus that can present with anemia, thrombocytopenia and prolonged fevers which is also supported by patient’s family history maternal lupus however may not necessarily have enlarged exudative tonsils and diffuse lymphadenopathy. Also, in this age group patients with lupus may have organ involvement specifically the kidney so one could check for proteinuria or send C3 or C4 which may be decreased in addition to ANA or dsDNA studies.
