# Supplementary material for: Skill Session on Writing Patient Assessments for Pediatric Clerkship Students
Source: MedEdPORTAL. 2020 Nov 9;16:11029. doi: 10.15766/mep_2374-8265.11029 (PMC7666838; doi:10.15766/mep_2374-8265.11029)
Supplement: Supplementary file 1 — PowerPoint Presentation.pptxInstructor Script.docxSample H&P 1.docxSample H&P 2.docxSample H&P 3.docxP-HAPEE Isolated Scoring Tool.docxAssessment Examples for Sample H&Ps.docxMedical Semantics Crossword.pdfCrossword Puzzle Answers.docx [file mep_2374-8265.11029-s001.zip › B. Instructor Script.docx]

**Appendix B**

**Skill Session: Formulating a Patient Assessment**

**Instructor Script**

Slide 1 Title of Session

The instructor can introduce him/herself on this slide and share an experience regarding how this topic became important to them. Example: *Academic physicians have the responsibility of grading student documentation. You may have personally noticed repeatedly that the patient assessment portion of the H&P is an area where students struggle -summarizing the patient and precisely stating their understanding of the patient by interpreting the key aspects of the case.*

Slide 2 Pre-session patient assessment

Start the session by having the students write a patient assessment for one of the sample H&Ps (Appendix C, D, or E). For a 90-minute session, students can complete this pre-session patient assessment at the beginning of the skill session. For a 60-minute session, students should complete this pre-session patient assessment prior to the skill session. Students can type in a document on their laptops or the instructor can provide a space on a learning management system like Canvas where written patient assessments can be completed, the latter being preferred. For accountability, participation, and later peer review it is encouraged that the instructor require completion of this activity in a learning management system.

 Prior to starting the pre-session activity it may be helpful to give a written definition of a patient assessment. Example:

*“An assessment statement is the synthesis of critical history, physical, and diagnostic findings described in succinct summative medical semantics leading to a most probable diagnosis and its differential. The differential diagnoses are compared and contrasted by discriminating features related to the patient’s presentation. Read the sample H&P provided and formulate your own written patient assessment.”*

Most students will be done in approximately 15 to 20 minutes. As the instructor you may walk around the room or get a raise of hands for students who have completed the pre-session activity and when majority of students are done you may collect the sample H&Ps and transition to the next slide.

Slide 3 Learning Objectives

Instructor can state the learning objectives and may say: “We will now go through the process of formulating an assessment. The objectives of this session are:

- First, to identify the essential components of a patient assessment.
- Second, instead of restating those components or information obtained during an H&P, learn to synthesize and interpret that information using precise medical semantics.
- Lastly, to practice using clinical reasoning to commit to a diagnosis and compare and contrast a differential by utilizing clinical reasoning rather than just listing a differential.”

A metaphor is not needed but sometimes can help the students relate more to what the learning objectives are trying to accomplish. Instructor may say

“Think of formulating an assessment as a process like cooking something flavorful where certain ingredients are the components and the specific order brings about the distinct flavor to reach a unique taste just like certain semantics produce a specific clinical scenario and clinical severity. Why is it that Remy from the Disney movie Ratatouille makes better dishes than Colette? Because although Remy uses most of the same ingredients he has a specific way in which he precisely places the ingredients which produces a more flavorful dish.”

Slide 4 Restatement of objectives in a simplified format

If instructor chooses to use a metaphor like that in Slide 3 then here in this slide you may simplify the objectives of this skill session by rephrasing the metaphor by saying

“Just like when cooking, we will first learn the *ingredients* of a patient assessment, then practice interpreting the patient presentation to make the assessment *more flavorful* by using precise medical terms, and lastly critique our own assessment recipe or statement by using clinical reasoning to compare and contrast a differential.”

The last part of this metaphor means that the recipe culminates to the diagnosis. If we were to alter the ingredients we may end up with a different dish. Similarly, we will discuss how to illustrate clinical reasoning using supporting or refuting information to discuss other differential diagnoses.

Slide 5 Definition of patient assessment

To engage the students you may first ask them what they think ‘patient assessment’ means or encompasses. Slide 5 provides some examples of phrases that refer to inquiring about the assessment of the patient. Instructor may choose to say

“In a clinical setting attending physicians are often asking for the patient assessment when saying these statements: ‘Give me the one-liner’, which is like a partial assessment when the diagnosis is known. Or when an attending asks ‘What is going on with the patient and why do you think that?’, they are actually asking for your patient assessment including your clinical reasoning.”

It is important to emphasize to the students that

“How well and thoroughly you state the patient assessment often shows how well you know your patient. Also, the clinical reasoning you provide when synthesizing the patient and discussing a differential diagnosis depicts a picture of your thought process and your knowledge base.”

Slide 6 Identify the essential components of a patient assessment (Objective 1)

The instructor may say here

“Starting with 0bjective 1, let’s identify the essential components of a patient assessment.”

This is a key slide that provides structure or a template for what information is gathered from the patient to formulate a complete patient assessment.

“The general order of information starts with age (include gestational age if premature often seen in pediatrics patients), sex, past medical history, pertinent summary of symptoms that were obtained from subjective portions of the history, signs which includes pertinent objective information like the interpreted physical exam, lab and imaging findings that all lead to a plausible diagnosis. Then, compare and contrast differential diagnoses coinciding with why you considered them and what in the H&P supports the differential diagnosis and what refutes it.”

This slide can be turned into an interactive activity. Some examples of how the instructor can turn this into an activity are: scramble the phrases written in red brackets on the slide and have students place them in the correct order or students can fill in the blanks by selecting phrases from a word bank.

 Because this slide gives a general template for students, provide this slide as a take home point or as reference post skill session that students can have access to later.

Slide 7 What makes a written patient assessment “Better”? (Objective 2)

This slide focuses on not only identifying or extracting the essential components needed to make a complete patient assessment but also summarizing and interpreting that information while constructing the patient assessment. The instructor may say

“It may seem easy to plug in or regurgitate all the information from the history and physical which is ‘good’ because you have all the essential components we just discussed in the last slide. However, how to make your patient assessment ‘better’ is actually to synthesize the pertinent information and interpret the patient data all while using precise medical terminology instead of just repeating what you may have already written or presented in the other portions of the patient presentation.”

“What makes a differential diagnosis ‘better’ is actually comparing and contrasting diagnoses by referencing pertinent negatives or pertinent positives from the case that support or refute the stated diagnosis rather than just listing a differential without stating why you think those diagnoses could also fit the clinical presentation, in other words, your clinical reasoning.”

Instructor and students can reference the P-HAPEE rubric tool, specifically sections/questions 8 and 9 in the rubric, used for grading of written H&Ps (from which is where this slide is adapted, also available in Appendix F) [Ref: King MA, Phillipi CA, Buchanan PM, Lewin LO. Developing validity evidence for the written pediatric history and physical exam evaluation rubric. Academic pediatrics. 2017 Jan 1;17(1):68-73.]

Slide 8 Defining medical terms and semantic qualifiers

Bridging from Slide 7 the instructor can say,

“Well, how can we synthesize and interpret the patient information rather than just restating the information we have obtained from the patient or the patient’s chart? One way to do that is by using cohesive medical terms and semantic qualifiers. Semantics means language that is used to achieve a desired effect on an audience especially through the use of words. Semantics provide precision of the problems, diagnoses, and presenting symptoms and signs.”

Referring back to the metaphor used earlier the instructor may say,

“In Ratatouille^©^, Remy’s dishes are more flavorful than Colette’s even though they use all the same ingredients. Its Remy’s precision with which he prepares the food and the slight differences in presentation that make it that way. In written patient assessments the semantic qualifiers can identify the clinical severity and may lead the reader to a specific diagnosis; for example, acute abdominal pain versus chronic abdominal pain paint a different differential picture in the reader’s mind . The words ‘acute’ and ‘chronic’ are semantic qualifiers. Medical terms often translate to diagnoses and problems. Medical terms can also overlap with semantic qualifiers. For example, ‘moderate’ is a semantic qualifier to dehydration, however, when used as ‘moderate dehydration’ this phrase is also now a more precise and specific medical diagnosis that indicates a different management plan (i.e. moderate dehydration indicates hospitalization is needed whereas mild dehydration may not). Not all medical terms may have or need a semantic qualifier. And, not all semantic qualifiers are necessarily followed by a medical term or diagnosis or problem. We will practice with medical terms and semantic qualifiers in the following slides.”

Instructor can point out the semantic qualifiers if present in the following examples.

Slide 9 Practice using precise medical semantics and synthesizing terminology when describing subjective findings in the patient assessment (Objective 2)

This slide lists several common examples that will provide the students practice with using medical terminology and semantic qualifiers. It may be unnecessary to show this slide and is listed here for the instructor’s reference. Each of the fragments of patient information is listed individually in Slides 10-18. These practice examples increase somewhat in the level of complexity.

Each practice example can be made as an interactive exercise using an audience response system like PollEverywhere in which each fragment of patient information is listed as an open-ended question type and students can respond by free-texting how they would synthesize or interpret the words or phrases in the example when summarizing and translating the findings in their patient assessment.

Slide 10-18 Practice summarizing subjective information using medical terms and semantic qualifiers.

These prompts/fragments of patient information are all listed here as in slide 9. The answers (the synthesized interpreted phrase using medical terminology and semantic qualifiers) are not on slide 9 but listed here for the instructor’s reference in **bold**. Instructor can ask the students for each slide, “How would you synthesize this subjective information obtained from a patient history?” Instructor can refer to student responses to point out correct answers or how to make those free text answers more precise. After some students have responded the instructor can say, “One way to synthesize this information in a patient assessment is [reference bolded text from list below].”

1. 3 days of cough, runny nose, congestion

**Acute onset URI symptoms**

1. Belly pain above the belly button

**Epigastric abdominal pain**

1. Whole body shaking episode, episode was 5 minutes after which the patient was sleepy

**Generalized seizure like episode followed by a post-ictal state**

1. Red rash started in neck area now down to legs

**Erythematous rash progressing in a cephalo-caudal pattern**

1. Difficulty breathing started today

**Acute onset respiratory distress secondary to...**

1. Patient always requires BIPAP support who comes in with one day of retractions, increased respiratory rate, requiring higher settings on respiratory support device

**Acute on chronic respiratory failure**

1. Diarrhea since birth; now stools have become bloody over the last 2 days

**Acute onset hematochezia in setting of chronic diarrhea**

1. A girl who had a UTI with fever due to multidrug resistant E. coli 3 months ago who comes in again with another UTI with fever

**Recurrent multidrug resistant E. coli pyelonephritis**

1. Sore throat, unable to swallow due to pain as evidenced by significant drooling and erythematous tonsils on exam

**Pharyngitis at risk of dehydration secondary to odynophagia**

Slide 10

1. 3 days of cough, runny nose, congestion

Instructor can ask the students,

“How would you synthesize this subjective information obtained from a patient history, ‘3 days of cough, runny nose, congestion’ in the assessment?”

Students can free text in an open ended question type in PollEverywhere what they think could be a synthesis using medical terms and precise descriptors. Instructor can refer to student responses to point out correct answers or how to make those free text answers more precise. After some students have responded the instructor can say,

“One way to synthesize this information in a patient assessment is ‘Acute onset URI symptoms’, acute onset being the semantic qualifier.”

Slide 11

1. Belly pain above the belly button

Instructor can ask the students,

“How would you say this subjective information obtained from the patient history more precisely using medical terms, ‘belly pain above the belly button’ in the assessment?”

Students can free text in an open ended question type in PollEverywhere what they think could be a more precise way of saying this pertinent information while using medical terms and precise descriptors. Instructor can refer to student responses to point out correct answers or how to make those free text answers more precise. After some students have responded the instructor can say,

“One way to state this subjective information more precisely and using appropriate medical terminology in a patient assessment is ‘Epigastric abdominal pain’, epigastric being like a semantic qualifier painting a differential in the reader’s mind and making the abdominal pain more specific.”

Slide 12

1. Whole body shaking episode, episode was 5 minutes after which the patient was sleepy

Instructor can ask the students,

“How would you summarize this subjective information that a patient or patient’s family has described when obtaining the history of present illness in the patient assessment ‘whole body shaking episode, episode was 5 minutes after which the patient was sleepy’?”

Students can free text in an open ended question type in PollEverywhere what they think could be a summarization and interpretation of this information using appropriate medical terms and precise descriptors. Instructor can refer to student responses to point out correct answers or how to make those answers more cohesive and precise. After some students have responded the instructor can say,

“One way to relay this subjective information in a patient assessment is ‘Generalized seizure-like episode of 5 minutes duration followed by a post-ictal state’, generalized being the semantic qualifier differentiating the episode from a focal episode. And, seizure-ike being the appropriate interpretation of the patient’s description of the episode, and post-ictal being an appropriate interpretation of the sleepiness afterwards.”

Slide 13

1. Red rash started in neck area now down to legs

Instructor can ask the students,

“How would you describe this subjective information using appropriate and precise medical terminology obtained from a patient in the patient assessment, ‘red rash started in neck area now down to legs’?”

Students can free text in an open ended question type in PollEverywhere what they think could be an appropriate translation using medical terms and semantic qualifiers. Instructor can refer to student responses to point out correct answers or how to make those answers more appropriate and descriptive. After some students have responded the instructor can say,

“One way to state this pertinent subjective and objective information using descriptive medical terminology in a patient assessment is ‘Erythematous rash spreading or progressing in a cephalo-caudal pattern’. Progressing is used as a semantic qualifier that describes the rash more clearly and as an indicator of clinical severity. Cephalo-caudal is used as an appropriate medical term that depicts a specific differential diagnosis in the listener’s/reader’s mind.”

Of note the instructor may state that this practice example also incorporates some aspects of the pertinent physical exam or objective findings as well which is okay because then the subjective and objective findings are being synthesized and described more succinctly in the patient assessment.

Slide 14

1. Difficulty breathing started today

Instructor can ask the students,

“How would you precisely state this subjective information obtained from the patient in your assessment, ‘difficulty breathing started today’?”

Remind students that these are common phrases that patients may tell the student when obtaining a history. The physician is supposed to interpret or summarize these pertinent phrases using precise medical terms that depict the presentation accurately. Students can free text in an open ended question type in PollEverywhere what they think could be an interpretation of this information using medical terms and precise descriptors. Instructor can refer to student responses to point out correct answers or how to make those answers more appropriate and descriptive. After some students have responded the instructor can say,

“One way to state this information more precisely using medical terminology in a patient assessment is ‘Acute onset respiratory distress’. Acute onset is used as a semantic qualifier specifically differentiating from chronic respiratory symptoms.”

This is also a good example for the instructor to point out that the acute onset respiratory distress will be a primary problem but is often secondary to an underlying cause such as an asthma exacerbation or an infection like pneumonia or croup. So students can say, ‘The acute onset respiratory distress secondary to… what process is underlying and causing the respiratory distress’.

Slide 15

1. Patient always requires BIPAP support who comes in with one day of retractions, increased respiratory rate, requiring higher settings on respiratory support device

Instructor can ask the students,

“How would you synthesize this subjective and objective information obtained from both the history, past medical history, and physical exam using precise and appropriate medical terms in the patient assessment, ‘patient always requires BIPAP support who comes in with one day of retractions, increased respiratory rate, requiring higher settings on respiratory support device in the assessment’?”

Students can free text in an open ended question type in PollEverywhere what they think could be an interpretation and synthesis of this information using medical terms and precise descriptors. Instructor can refer to student responses to point out correct answers or how to make those answers more appropriate, succinct, and descriptive. After some students have responded the instructor can say,

“One way to summarize and interpret this information in a patient assessment is ‘Acute on chronic respiratory failure’. Acute on chronic is the phrase being used as a semantic qualifier indicating the chronic need for respiratory support but acute worsening indicating clinical severity. Respiratory failure is the interpretation that the patient requires respiratory support to breathe.”

Remind the students that they need not restate all the findings that they may have already relayed when presenting the history, past medical history and physical exam but interpret what those findings mean in the patient assessment. For example, the current acute respiratory distress may be the interpretation of the use of accessory muscles to breathe (i.e. the retractions) and tachypnea (i.e. the increased respiratory rate) already stated in the physical exam portion of the patient presentation. This is also an example that combines subjective and objective components.

Slide 16

1. Diarrhea since birth but now have become bloody over the last 2 days

Instructor can ask the students,

“How would you synthesize the subjective information using appropriate medical terminology and semantic qualifiers in the patient assessment, ‘diarrhea since birth but now stools have become bloody over the last 2 days’?”

Students can free text responses in open ended question type in PollEverywhere what they think could be a synthesis of this information using medical terms and precise descriptors. Instructor can refer to student responses to point out correct answers or how to make those answers more appropriate, succinct, and descriptive. After some students have responded the instructor can say,

“One way to more precisely synthesize and translate this subjective information in a patient assessment with appropriate medical terminology and semantic qualifiers is ‘Acute onset hematochezia in setting of chronic diarrhea’. Hematochezia is the appropriate medical term differentiating the bloody stool from melena. Acute onset and chronic being the semantic qualifiers describing the time course of the symptoms more precisely.”

Slide 17

1. A girl who had a UTI with fever due to multidrug resistant E. coli 3 months ago who comes in again with another UTI with fever

Instructor can ask students,

“How would you summarize this subjective information obtained from the patient and likely from some chart review of her past history, ‘a girl who had a UTI with fever due to multidrug resistant E. coli 3 months ago who comes in again with another UTI with fever’?”

Students can free text responses in open ended question type in PollEverywhere what they think could be a more succinct and precise summary of this information using appropriate descriptive medical terms. Instructor can refer to student responses to point out correct answers or how to make those answers more appropriate, succinct, and descriptive. After some students have responded the instructor can say,

“One way to succinctly summarize this information in a patient assessment is ‘Recurrent multidrug resistant (MDR) E. coli pyelonephritis or upper urinary tract infection or urosepsis’. Recurrent is used as a more succinct semantic qualifier which also quickly identifies clinical severity making this diagnosis or problem concerning because it has occurred again. Presence of fever indicates either urosepsis or upper pole of kidney/ureter involvement as opposed to a lower urinary tract.”

Slide 18

1. Sore throat, unable to swallow due to pain as evidenced by significant drooling and erythematous tonsils on exam

Instructor can ask students,

“How would you summarize this subjective and objective patient information in the patient assessment using appropriate descriptive medical terminology ‘sore throat, unable to swallow due to pain as evidence by significant drooling and erythematous tonsils on exam’?”

Students can free text responses in open ended question type in PollEverywhere what they think could be an interpretation of this information using medical terms and precise descriptors. Instructor can refer to student responses to point out correct answers or how to make those answers more appropriate, succinct, and descriptive. After some students have responded the instructor can say,

“This is another example that also combines history and physical exam findings. One way to state this information in a patient assessment is ‘Pharyngitis at risk of dehydration secondary to odynophagia’. A semantic qualifier phrase in this example is ‘at risk of’ indicating clinical severity that necessitates hospitalization.”

This example is also more complex because it addresses some clinical severity while describing the subjective and objective findings. This example also illustrates that the primary problem may be the pharyngitis but a secondary problem to be addressed is the risk of dehydration.

Slide 19 Practice using precise medical semantics when interpreting objective findings in the assessment (0bjective 2)

This slide lists several common examples that will provide the students practice with interpretation of physical exam findings and laboratory data while using medical terminology and semantic qualifiers. It may be unnecessary to show this slide and is listed here for the instructor’s reference. Each of the fragments of patient information is listed individually in Slides 20-31. These practice examples increase somewhat in the level of complexity.

Each practice example can be made as an interactive exercise using an audience response system like PollEverywhere in which each fragment of patient information is listed as an open-ended question type and students can respond by free-texting how they would synthesize or interpret the words or phrases in the example when summarizing and translating the findings in their patient assessment.

Instructor may say on this slide,

“Although some of the prior practice examples did combine aspects of the physical exam, now we will do more practice examples interpreting commonly found physical exam and laboratory findings.”

Slides 20-31 Practice interpreting objective information using medical terms and semantic qualifiers.

These prompts/fragments of patient information are all listed here as in slide 19. The answers (synthesized interpreted phrases using descriptive medical terminology and semantic qualifiers) are not on slide 19 but listed here for the instructor’s reference in **bold**. Instructor can ask students “How would you state your interpretation of these objective findings using precise medical terminology in the patient assessment?” Instructor can refer to student responses to point out correct answers or how to make those answers more the interpretation rather than repeating the exam finding and demonstrate how the answers could be more descriptive. After some students have responded the instructor can say, “One way to state this objective information in a patient assessment is [refer to appropriate bolded information from list below].”

1. Wheezing throughout on exam

**Diffuse wheezing**

1. Tenderness to palpation on abdominal exam but mostly on the right side

**Diffuse abdominal tenderness localizing to the right quadrant**

1. 3/5 strength in legs

**Diminished strength in lower extremities**

1. Resistance appreciated when moving ankle

**Decreased passive range of motion of the right ankle joint**

1. On exam baby not crying tears, delayed capillary refill, and tachycardia noted on vitals

**Moderate dehydration**

1. Infant using all accessory muscles to breathe –  tachypnea, retractions, nasal flaring, who during your exam starts to have grunting and becoming more sleepy not interested in feeding or playing

**Moderate to severe respiratory distress impending respiratory failure**

1. Venous blood gas shows 7.12, CO_2_ of 60

**Uncompensated respiratory acidosis concerning for acute hypercapneic respiratory failure**

1. Urine analysis has bacteria and microscopy has 25-30 white blood cells per high power field

**Bacteriuria and pyuria concerning for UTI**

1. CBC has white blood cells of 2,000 Hemoglobin of 7 and Platelets of 47,000

**Pancytopenia**

1. Baby who has been vomiting for one day has BMP that shows sodium 155, K 2.0, Cl 97, Bicarb 27, BUN 30, Cr 0.9, Gluc 45

**Acute onset vomiting causing electrolyte derangements showing hypernatremic hypokalemic hypochloremic metabolic alkalosis with dehydration as evidenced by prerenal azotemia**

1. Patient fever for 2 days, tachycardia, blood pressures below normal for age

**Septic shock (if blood pressure not affected then meets criteria for SIRS or systemic inflammatory response syndrome)**

1. Anemia on CBC evaluation with a low MCV and iron studies show low iron level

**Microcytic anemia secondary to iron deficiency**

Slide 20

1. Wheezing throughout on exam

Instructor can ask students,

“How would you state your interpretation of this physical exam finding using precise medical terminology in the patient assessment, ‘wheezing throughout on exam’?”

Students can free text responses in open ended question type in PollEverywhere what they think could be an interpretation of this objective information using appropriate medical terms. Instructor can refer to student responses to point out correct answers or how to make those answers more the interpretation rather than repeating the exam finding and demonstrate how the answers could be more descriptive. After some students have responded the instructor can say,

“One way to state this objective information in a patient assessment is ‘Diffuse wheezing’ differentiating from local or focal wheezing that would trigger thought processes on other differentials.”

Slide 21

1. Tenderness to palpation on abdominal exam but mostly on the right side

Instructor can ask students,

“How would you summarize and interpret these objective physical exam findings using appropriate and descriptive medical terminology in the patient assessment, ‘tenderness to palpation on abdominal exam but mostly on the right side’ in the patient assessment?”

Students can free text responses in open ended question type in PollEverywhere what they think could be a descriptive interpretation of this information using appropriate medical terminology. Instructor can refer to student responses to point out correct answers or how to make those answers more the interpretation and summary rather than repeating the exam finding and demonstrate how those answers could be made more descriptive. After some students have responded the instructor can say,

“One way to summarize and interpret this information in a patient assessment is “Diffuse abdominal tenderness localizing to right quadrant. Localizing and diffuse are the semantic qualifier terms that describe the abdominal pain more precisely triggering thought processes for certain differential diagnoses.”

Slide 22

1. 3/5 strength in legs

Instructor can ask students,

“How would you interpret ‘3/5 strength in the legs’ in the patient assessment instead of just restating the numeral exam findings?”

Students can free text responses in open ended question type in PollEverywhere what they think could be an interpretation of this information using appropriate descriptive medical terms. Instructor can refer to student responses to point out correct answers or how to make those answers more interpretative rather than repeating the exam finding. After some students have responded the instructor can say,

“One way to state this information by interpreting without repeating the exam findings in a patient assessment is ‘Diminished strength in lower extremities’.”

This example may seem very simple but it emphasizes to the students that specific numerals whether they are part of the vital signs or part of the exam findings do not need to be repeated rather should be interpreted to what those numbers or numerals mean/indicate in the patient’s written assessment.

Slide 23

1. Resistance appreciated when moving ankle

Instructor can ask students,

“How would you interpret this physical exam finding ‘resistance appreciated when moving ankle’ in the patient assessment?”

Students can free text responses in open ended question type in PollEverywhere what they think could be an interpretation of this information using appropriate medical terms. Instructor can refer to student responses to point out correct answers or how to make those answers more interpretative rather than repeating the exam finding. After some students have responded the instructor can say,

“One way to interpret this information in a patient assessment is ‘Decreased passive range of motion of right ankle joint indicating limited/restricted mobility’.”

Slide 24

1. On exam baby not crying tears, delayed capillary refill, and tachycardia noted on vitals

Instructor can ask students,

“How would you synthesize and interpret these physical exam findings into a cohesive phrase in the patient assessment, ‘on exam baby not crying tears, delayed capillary refill, and tachycardia noted on vitals’?”

Students can free text responses in open ended question type in PollEverywhere what they think could be an interpretation of this information using medical terms and precise descriptors. Instructor can refer to student responses to point out correct answers or how to make those answers more a synthesis and an interpretation of those findings rather than repeating the exam finding. After some students have responded the instructor can say,

“One way to synthesize and interpret without repeating this information in a patient assessment is ‘Moderate to severe dehydration’. Moderate to severe are being used as semantic qualifiers that illustrate a medical diagnosis and indicate clinical severity. The phrase moderate to severe also indicates that there are findings on exam that qualify the patient for having moderate dehydration and some findings on exam that support severe dehydration.”

Slide 25

1. Infant using all accessory muscles to breathe – tachypnea, retractions, nasal flaring, who during your exam starts to have grunting and becoming more sleepy not interested in feeding or playing,

Instructor can ask students

“How would you synthesize and interpret these physical exam findings to reflect what is going on with the infant in the patient assessment, ‘infant using all accessory muscles to breathe- tachypnea, retractions, nasal flaring, who during your exam starts to have grunting and becoming more sleepy not interested in feeding or playing’?”

Students can free text responses in open ended question type in PollEverywhere what they think could be an interpretation and synthesis of this information using appropriate medical terms and semantic qualifiers. Instructor can refer to student responses to point out correct answers or how to make those answers more a synthesis and an interpretation of these findings rather than repeating the exam findings. After some students have responded the instructor can say,

“One way to synthesize and interpret this information in a patient assessment is ‘Moderate to severe respiratory distress impending respiratory failure’. Moderate to severe is a phrase used as the semantic qualifier describes more precisely the respiratory distress and the word impending being a semantic qualifier that indicates and depicts the clinical severity.”

Slide 26

1. Venous blood gas shows 7.12, CO_2_ of 60

Instructor can ask students,

“How would you interpret the lab data from this venous blood gas in the patient assessment without repeating the numbers?”

Students can free text responses in open ended question type in PollEverywhere what they think could be an interpretation of this information using appropriate medical terms. Instructor can refer to student responses to point out correct answers or how to make those answers a descriptive interpretation of the lab findings rather than repeating the lab data. After some students have responded the instructor can say,

“One way to interpret and describe these objective lab data findings in a patient assessment is uncompensated respiratory acidosis concerning for acute hypercapneic respiratory failure’. Uncompensated and acute are examples of semantic qualifiers.”

Again, this example emphasizes the need to interpret the lab data and what it may indicate in the written patient assessment rather than repeating the numbers again.

Slide 27

1. Urine analysis has bacteria and microscopy has 25-30 white blood cells per high power field

Instructor can ask students,

“How would you interpret this urine analysis ‘bacteria and microscopy has 25-30 white blood cells per high power field’ without restating the numbers in the patient assessment?”

Students can free text responses in open ended question type in PollEverywhere what they think could be an interpretation of this information using appropriate medical terms and precise descriptors. Instructor can refer to student responses to point out correct answers or how to make those answers a descriptive interpretation of the lab fin,dings rather than repeating the lab data. After some students have responded the instructor can say

“One way to state this information in a patient assessment is ‘Urine analysis with bacteriuria and pyuria consistent with urinary tract infection’; bacteriuria and pyuria are the medical terms describing the lab findings and interpreting those finding by saying they are consistent with a UTI.”

Slide 28

1. CBC has white blood cells of 2,000 Hemoglobin of 7 and Platelets of 47,000

Instructor can ask students,

“How would you interpret these CBC findings using appropriate medical terminology without restating the numbers in the patient assessment?”

Students can free text responses in open ended question type in PollEverywhere what they think could be an interpretation of this information using medical terms and precise descriptors. Instructor can refer to student responses to point out correct answers or how to make those answers an interpretation of the lab findings rather than repeating the lab data. After some students have responded the instructor can say,

“One way to interpret this information in a patient assessment is the CBC shows ‘pancytopenia’. You can further indicate clinical severity and use semantic qualifiers to describe neutropenia severity (mild, moderate, severe) by stating your calculated absolute neutrophil count (ANC). If patient is symptomatic from anemia you can use the semantic qualifier ‘symptomatic’ anemia.”

Slide 29

1. Baby who has been vomiting for one day has BMP that shows sodium 155, K 2.0, Cl 97, Bicarb 27, BUN 30, Cr 0.9, Gluc 45

Instructor can ask students,

“How would you summarize the subjective report from the family that the infant has been vomiting for one day and interpret these objective laboratory data into the patient assessment using appropriate medical terminology?”

Students can free text responses in open ended question type in PollEverywhere what they think could be an interpretation of this information using medical terms and precise descriptors. Instructor can refer to student responses to point out correct answers or how to make those answers a better summary and more descriptive interpretation of the subjective and objective patient information. After some students have responded the instructor can say,

“One way to summarize and interpret all this information from the history of present illness and the objective laboratory data in a patient assessment is ‘Infant with acute onset vomiting with sequelae concerning for hypernatremic dehydration as evidenced by prerenal azotemia and acute kidney injury with multiple electrolyte derangements including hypokalemic, hypochloremic metabolic acidosis, in addition to hypoglycemia’. These qualifying phrases and medical terminology along with interpretation of the patient data describe all the medical problems that the patient has rather than just restating all the numbers. This shows your understanding and interpretation of the problems that this patient has currently.”

This is a complex practice example of how to combine both subjective and objective patient information so that it accurately describes your interpretation of the patient’s problems. The primary problem being the vomiting that then caused all the secondary problems.

Slide 30

1. Patient fever for 2 days, tachycardia, blood pressures below normal for age

Instruct0r can ask students,

“How would you combine and interpret the subjective information that the patient has had fever for 2 days and objective physical exam findings of tachycardia and blood pressures being below normal for age without just restating these words in the patient assessment?”

Students can free text responses in open ended question type in PollEverywhere what they think could be an interpretation of this information using medical terms and precise descriptors. Instructor can refer to student responses to point out correct answers or how to make those answers a synthesis of the subjective and objective patient information. After some students have responded the instructor can say,

“One way to synthesize this information in a patient assessment is ‘Septic shock secondary to….’ This example illustrates that the interpretation of the subjective and objective information using appropriate medical terms describe the problems for the patient. The primary problem in this patient being the septic shock and the secondary problem may be the infection that the septic shock is due to, for example, viremia or meningitis or E. coli urinary tract infection, etc..”

Slide 31

1. Anemia on CBC evaluation with a low MCV and iron studies show low iron level

Instructor can ask students,

“How would you interpret and synthesize this objective patient information in the patient assessment, ‘anemia on CBC with low MCV and iron level being low’?”

Students can free text responses in open ended question type in PollEverywhere what they think could be a synthesis and interpretation of this information using medical terms and precise descriptors. Instructor can refer to student responses to point out correct answers or how to make those answers more concise using medical terminology. After some students have responded the instructor can say,

“One way to synthesize and interpret this information in a patient assessment is ‘Microcytic anemia secondary to iron deficiency’. Microcytic is the semantic qualifier; however, microcytic anemia is also a more precise medical diagnosis.”

Slides 32 and 33 Analyze a ‘Good’ written patient assessment and make it ‘Better’ by using synthesized and interpretive medical terms and semantic qualifiers (Objectives 2 and 3)

As a group, the instructor and students will have dialogue about other differential diagnoses that could be considered. Students are expected to support or refute their offered diagnosis with their clinical reasoning. Instructor can reference the P-HAPEE grading rubric sections/questions 8 and 9 again to discuss what score the before and after examples would receive (Appendix F).

Slide 32 Practice synthesis and clinical reasoning to discuss differential diagnosis (Objectives 2 and 3)

Although this slide can be done as an oral discussion, another way to make it interactive is to copy the ‘Before’ patient assessment example into an image [Ctrl-Alt-PrtSc🡪 paste into paint🡪 save image 🡪 insert image into Clickable Image in PollEverywhere].

The first task of this example addresses Objective 2. Students can drop arrows to areas where they think the wording could better reflect synthesized medical terms and semantic qualifiers. Discuss the arrows by asking students to state their suggestions at how those areas could be changed to reflect more precise medical terminology. After this group discussion the ‘After’ patient assessment example can be revealed. In the PowerPoint the underlined and green font phrases in the ‘After’ patient assessment indicate patient information that are the improved phrases using medical semantics. The following is the ‘After’ patient assessment. The underlined words and phrases here reflect the summarized and descriptive medical terminology to make the assessment better.

2-year-old former 24-week premature boy with history of necrotizing enterocolitis s/p bowel resection presenting with moderate dehydration secondary to acute vomiting now bilious with abdominal distention on exam concerning for surgical abdomen consistent with bowel obstruction.

The second purpose of this example is to practice Objective 3. Have students offer other diagnoses that could possibly be considered given the clinical presentation in the patient assessment example. Have students suggesting those diagnoses state their clinical reasoning in how those diagnoses may or may not be supported describing discriminating features between those diagnoses. For the example in this slide, the possible differential diagnoses and their coinciding clinical reasoning could be: “Gastroenteritis is a possible diagnosis supported by the presence of significant vomiting and the acute nature of the onset; however, the absence of diarrhea in the clinical presentation does not support this diagnosis. Pyloric stenosis is a possible diagnosis supported by the patient’s significant vomiting; however, the patient’s age is not the typical age at which this disorder usually presents and this diagnosis is also refuted by the presence of bilious vomiting. Patients with pyloric stenosis generally do not have bilious vomiting.”

 It is important to mention here that as students are summarizing the patient presentation in the assessment, the precise medical terms being used also often identify the problems and diagnoses that the patient has. These problems can be listed as separate diagnoses or problems that need to be addressed usually by severity (#1 being the most acute problem that needs to be addressed first). So for the example in this slide, the patient assessment example has a primary diagnosis or problem of bowel obstruction. The secondary diagnosis or problem is moderate dehydration from acute bilious vomiting.

Slide 33 Practice synthesis and clinical reasoning to discuss differential diagnosis (Objectives 2 and 3)

Although this slide can be done as an oral discussion, another way to make it interactive is to copy the ‘Before’ patient assessment example into an image [Ctrl-Alt-PrtSc🡪 paste into paint🡪 save image 🡪 insert image into Clickable Image in PollEverywhere].

The first task of this example addresses Objective 2. Students can drop arrows to areas where they think the wording could better reflect synthesized medical terms and semantic qualifiers. Discuss the arrows by asking students to state their suggestions at how those areas could be changed to reflect more precise medical terminology. After group discussion the ‘After’ patient assessment example can be revealed. In the PowerPoint the underlined and green font phrases in the ‘After’ patient assessment indicate the improved phrases using medical semantics. The following is the ‘After’ patient assessment. The underlined words and phrases reflect suggestions to make a better more descriptive and summative patient assessment.

3-year-old (vaccinated) girl with history of constipation presenting with acute onset fever and odynophagia with decreased oral intake and inability to handle secretions; concerning exam findings for neck tenderness and decreased range of motion of neck with enlarged tonsils who has a neutrophilic leukocytosis. This presentation is most consistent with…

The second purpose of this example is to practice Objective 3. Have students offer other diagnoses that could possibly be considered given the clinical presentation in the patient assessment example. Have students suggesting those diagnoses state their clinical reasoning in how those diagnoses may or may not be supported describing discriminating features between those diagnoses. For the example in this slide the possible differential diagnoses and their coinciding clinical reasoning could be: “Peritonsillar abscess is a possible diagnosis supported by odynophagia; however, absence of an asymmetrically enlarged tonsil on physical exam does not support this diagnosis. Meningitis is a diagnosis to consider and is supported by the presence of fever and decreased range of motion of the neck; however, meningitis is usually associated with more irritability. Meningitis may still be a possibility which may warrant further studies like x,y, and z. Bacterial tracheitis is a possible diagnosis supported by the inability to handle secretions and the patient appearing more toxic; even though currently the presentation is not consistent with bacterial tracheitis the patient could progress to bacterial tracheitis. Epiglottitis is a diagnosis to consider supported by the drooling and inability to handle secretions; however, not supported by the absence of tripod or dog sniffing positioning. This diagnosis would also be more common in an unvaccinated patient. An EBV infection can also be considered as it is a common cause of pharyngitis as this patient has; however, not supported by the absence of prolonged fever and symptoms and the absence of atypical lymphocytosis on the CBC”.

Slide 34 Applying Objectives 1, 2, and 3 to a sample H&P

Give the students some time to read the H&P and after 5 minutes ask for a volunteer student to suggest a patient assessment to the sample H&P which the instructor may write up on a white board or type up to make visible to all the students. Students participate in suggesting areas of the stated assessment that could reflect more summarizing medical terminology and utilize more clinical reasoning similar to how it was done in the prior two examples.

Have students verbally discuss further differential diagnoses they have suggested and ask them to provide their clinical reasoning by stating supporting or absence of supporting information.

Slide 35 Sample H&P from Slide 34 Example Patient Assessments from ‘Good’ to ‘Better’

Using the assessment provided by the student volunteer, ask students how to make this a ‘Better’ written assessment using more summarizing and interpretive medical terms and semantic qualifiers.

A sample ‘Good’ patient assessment to the sample H&P in Slide 34 is offered here:

2-year-old 32-week premature boy who has past medical history of being hospitalized for RSV bronchiolitis and requiring CPAP at birth presents with difficulty breathing found to have increased work of breathing using accessory muscles to breathe and wheezing on exam most consistent with acute asthma exacerbation secondary to a URI illness. Other diagnoses to consider would be pneumonia supported by fever and crackles on physical exam; however, CXR does not show a focal infiltrate versus foreign body aspiration in setting of acute onset of respiratory distress but not supported by absence of focal findings on physical exam or on chest xray.

The underlined green font areas in the PowerPoint correlate with suggested modifications using more precise medical terminology.

2-year-old 32-week premature boy who has past medical history of being hospitalized for RSV bronchiolitis and requiring CPAP at birth presents with acute hypoxic respiratory failure found to have increased work of breathing using accessory muscles to breathe, hypoxia and diffuse wheezing on exam most consistent with acute asthma exacerbation secondary to URI illness. Other diagnoses to consider would be pneumonia supported by fever, crackles on physical exam and neutrophilic leukocytosis; however, the CXR does not show a focal infiltrate. Another diagnosis to consider is foreign body aspiration in setting of acute onset of respiratory distress and the patient’s age as well as the hypercarbia or CO_2_ retention suggests an obstructive process; however, this diagnosis is not supported by the absence of focal findings on physical exam or on chest x-ray.

Then the instructor can ask the students to offer other plausible differential diagnoses with coinciding clinical reasoning from the sample H&P in Slide 34 that could support or refute those diagnoses.

Slide 36 End of session activity

At the end of the skill session have students apply their learning to the same or another selected sample H&P (Appendices C, D, or E) and formulate a written patient assessment. This portion of the skill session takes about 15 minutes for students to formulate one patient assessment and another 5 minutes for the instructor to review a ‘sample answer’. All sample answers are provided in the slides for completeness. It is preferential that students do this end of session patient assessment during the skill session rather than on their own time because the following slides review suggested sample answers and allow for further discussion with the students on how they may have written some aspects differently. The discussion after students have completed their patient assessments also allows for further application of Objective 3 where students suggest other differential diagnoses not mentioned in the sample answers with associated clinical reasoning.

Slides 37-39 Provide examples of written patient assessments with associated differential diagnoses. Apply Objectives 1, 2, and 3. These are all also provided in Appendix G. The bolded text in the presentation and in the Appendix demonstrate application of Objective 2, phrases utilizing summarizing and interpretive medical terms with semantic qualifiers to depict the patient’s presentation.

Slide 37 Sample Answer/Example Written Patient Assessment and discussion of Differential Diagnosis to Sample H&P 1

Instructor can have students further demonstrate Objective 3 by having a verbal discussion with the students about suggesting other differential diagnoses and how those would be supported and/or not supported practicing stating their clinical reasoning.

Slide 38 Sample Answer/Example Written Patient Assessment and discussion of Differential Diagnosis to Sample H&P 2

Instructor can have students further demonstrate Objective 3 by having a verbal discussion with the students about suggesting other differential diagnoses and how those would be supported and/or not supported practicing stating their clinical reasoning.

Slide 39 Sample Answer/Example Written Patient Assessment and discussion of Differential Diagnosis to Sample H&P 3

Instructor can have students further demonstrate Objective 3 by having a verbal discussion with the students about suggesting other differential diagnoses and how those would be supported and/or not supported practicing stating their clinical reasoning.
