# Supplementary material for: Skill Session on Writing Patient Assessments for Pediatric Clerkship Students
Source: MedEdPORTAL. 2020 Nov 9;16:11029. doi: 10.15766/mep_2374-8265.11029 (PMC7666838; doi:10.15766/mep_2374-8265.11029)
Supplement: Supplementary file 1 — PowerPoint Presentation.pptxInstructor Script.docxSample H&P 1.docxSample H&P 2.docxSample H&P 3.docxP-HAPEE Isolated Scoring Tool.docxAssessment Examples for Sample H&Ps.docxMedical Semantics Crossword.pdfCrossword Puzzle Answers.docx [file mep_2374-8265.11029-s001.zip › C. Sample H&P 1.docx]

**Sample H&P (1)**

**Formulate Your Own Assessment**

**Instructions: Read the following H&P. Synthesize the patient presentation and use clinical reasoning to formulate your own assessment and differential diagnosis. Arrows denote the direction of an abnormal value.**

**CC:** “very dark urine”

**HPI**: 7-year-old girl who presents with one day history of not urinating as much as before and dark colored urine. Mom states that patient’s illness began with symptoms of vomiting one week prior. She started having diarrhea and abdominal pain around the same time. Vomiting now resolved but was occurring 3-4 times a day or whenever she would try to eat something. Diarrhea is occurring 3-4 times a day. The diarrhea became bloody on day of admission. Prior to onset of symptoms patient was at a birthday party at a park where there were several food items like barbecued hamburger patties, potato salad, and fruit. The birthday party hosts also had a small petting zoo where all the children played. Mom has also noticed that the patient has been urinating less throughout her illness but attributed it to her not drinking as much. Her urine is darker now and smells bloody. The patient had been complaining of abdominal pain since the onset of vomiting and diarrhea. The patient has prior history of abdominal pain every now and then. Her PCP diagnosed her with constipation in the past as a cause for her abdominal pain. Mom is not aware if any of the other children at the party are also ill with similar illness.

**ROS**:

General: +fever for last one day, more tired in the last week

HENT: +headache in the last 1 day, no sore throat, no history of ear infections

Eyes: no eye drainage, no vision problems

GI: +vomiting, +diarrhea, +bloody stools today, history of constipation, +abdominal pain

Cardiac: deny any murmurs

Pulmonary: no difficulty breathing,

GU: +urine is darker in color concerning for blood, +urinating less than previously

Neuro: +headache

Heme: no bruising noted but patient appears more pale than previously

Endo: normal newborn screens

Allergy: no known allergies

**PMH**:

Birth: 38 weeks gestation, no NICU stay but required phototherapy for one day

Other diagnoses and surgeries: RSV as infant hospitalized for 3 days, constipation, diagnosed with urinary tract infection when she was 5 years old for which she received antibiotics, no other surgeries

Social: attends 2^nd^ grade, one dog at home

Meds: Miralax for constipation, Tylenol and ibuprofen for fever, multivitamin

Family History: Dad with ulcerative colitis, older teenage brother and mom with history of kidney stones

**Physical Exam**:

Vitals: T. **103 F** **↑**, HR **140** bpm **↑**, RR 24, BP **120/72↑**, O_2_ sat 97% on room air

General: awake and interactive but looks like she doesn’t feel well, pale appearance

HEENT: normocephalic, atraumatic; tacky mucous membranes, oropharynx without erythema or any lesions, TMs clear bilaterally, nasal turbinates not boggy or inflamed

Lymph: no palpable lymphadenopathy

Respiratory: clear to auscultation throughout, no nasal flaring, no retractions or belly breathing

Cardiac: tachycardic, normal S1 and S2, soft systolic murmur appreciated, capillary refill approximately 4 seconds

GI: soft, flat, tenderness to palpation in epigastrium, middle of abdomen, and below the belly button, no masses appreciated, no enlarged liver or spleen palpated

Back: right sided CVA (costovertebral angle) tenderness

GU: Tanner Stage 1 female, no skin tags or fissures noted on perianal exam

Neuro: strength 5/5 in arms, 5/5 in legs, deep tendon reflexes 2+ , cranial nerves in tact

Skin: no rashes, no birthmarks, no nevi, two faint 3 cm bruises/ecchymoses noted both on lower extremities over bony surfaces

**Labs/Imaging**:

Na 135, K **5.3↑**, Cl 103, Bicarb **14↓**, BUN **42↑**, Cr **1.5↓**, Gluc 76, Ca 9.0

WBC: **21,000 ↑** diff showing **80% neutrophils↑**, 19% lymphocytes, 1% monophils,

Hemoglobin **7.2** **↓** MCV 87 (nl)

Platelets **45,000↓**

CBC smear **+schistocytes**

UA: Spec gravity **1.044**↑, **2+blood, +ketones, +protein, +leukocyte esterase**, negative nitrites, negative glucose
