# Supplementary material for: Skill Session on Writing Patient Assessments for Pediatric Clerkship Students
Source: MedEdPORTAL. 2020 Nov 9;16:11029. doi: 10.15766/mep_2374-8265.11029 (PMC7666838; doi:10.15766/mep_2374-8265.11029)
Supplement: Supplementary file 1 — PowerPoint Presentation.pptxInstructor Script.docxSample H&P 1.docxSample H&P 2.docxSample H&P 3.docxP-HAPEE Isolated Scoring Tool.docxAssessment Examples for Sample H&Ps.docxMedical Semantics Crossword.pdfCrossword Puzzle Answers.docx [file mep_2374-8265.11029-s001.zip › D. Sample H&P 2.docx]

**Sample H&P (2)**

**Formulate Your Own Assessment**

**Instructions: Read the following H&P. Synthesize the patient presentation and use clinical reasoning to formulate your own assessment and differential diagnosis. Arrows denote the direction of an abnormal value.**

**CC:** “bloody stools”

**HPI**: 15-year-old boy presents with bloody stools. Mom states that patient has been having bloody stools that started three days ago. Mom does note that patient has had diarrhea on and off for the last 3-4 months. She says that he has complained of some belly pain sometimes associated with the diarrhea. Mom has been giving the patient Ibuprofen and Pepto-Bismol to help with the belly pain and diarrhea. The diarrhea would get better so mom thought maybe it was due to something he was eating outside the home since he would often go over to a friend’s house after school to play video games. Now since the onset of the bloody stools the belly pain has gotten more intense but the patient states that the belly pain improves after he has a bowel movement. He has been having 6-7 stools a day whereas before he would only stool 2-3 times a day. He reports having to get up at night to have a bowel movement over the last two days. No vomiting. Some nausea when he has the belly pain. He has not recently travelled anywhere. No known sick contacts. Did eat some exotic seafood like shell fish at a recent family party one week prior.

**ROS**:

General: no fever, +significant weight loss mom thinks approx 15-20 pounds in the last year since his last physical exam by PCP, +fatigue

HENT: no history of ear infections, no head trauma, +sore throat, no cough or URI symptoms

Eyes: no eye drainage, no vision problems

GI: +diarrhea, +bloody stools as per HPI

Cardiac: deny any murmurs

Pulmonary: no difficulty breathing

GU: +urine is darker in color, +urinating less than previously

Neuro: no delays, denies any seizures

Heme: no bruising noted but patient appears more pale than previously

Endo: normal newborn screens

Allergy: does not eat dairy, no environmental allergies

**PMH**:

Birth: 38 weeks gestation, no NICU stay but required phototherapy for one day

Other diagnoses and surgeries: milk protein allergy as infant, broken elbow while playing on monkey bars at age 9

Social: attends 10th grade, one dog at home,

HEADDS exam: pertinent for patient not doing well in school, frequently absent, smokes marijuana reports trying heroin once, sexually active with 2 female partners in the last year

Meds: none

Family History: cousin with ulcerative colitis, mom with irritable bowel syndrome, hypothyroidism, cousin with Type 1 diabetes mellitus

**Physical Exam**:

Vitals: T. 99 F HR **115** **↑**bpm, RR 20, BP 100/70, O_2_ sat 99% on room air

General: awake and interactive in no acute distress, pale and thin appearance

HEENT: normocephalic, atraumatic; tacky mucous membranes, oropharynx mildly erythematous with ulcers noted on tongue, TMs clear bilaterally, nasal turbinates not boggy or inflamed

Lymph: palpable lymph nodes approx 2-3 cm in cervical, axillary, and inguinal region

Respiratory: clear to auscultation throughout, no nasal flaring, no retractions or belly breathing

Cardiac: tachycardic, normal S1 and S2, II/VI systolic ejection murmur appreciated, capillary refill approximately 4 seconds

GI: soft, flat, tenderness to palpation of entire abdomen but worse in left quadrant, no masses appreciated, no enlarged liver or spleen palpated

GU: Tanner Stage 3 male, +fissure at12:00 o’clock on perianal exam

Neuro: strength 5/5 in arms, 5/5 in legs, deep tendon reflexes 2+ , cranial nerves in tact

Skin: no rashes, no birthmarks , no nevi, no bruises

**Labs/Imaging**:

Na 137, K 3.0, Cl 103, Bicarb **16↓**, Anion gap 18 (nl), BUN 25, Cr 0.9, Gluc **185↑**, Ca 9.0, Albumin **2.2↓**

WBC: 12,000, diff showing 60% neutrophils, 34% lymphocytes, 3% monophils, 3% eosinophils

Hemoglobin **8.0↓,** MCV 89 (nl), Platelets 400K

**CRP elevated**

**ESR elevated**

**VBG: 7.28 (pH)↓/**45 (CO_2_)/55 (O_2_)**/16 (Bicarb)↓**
