# Supplementary material for: Skill Session on Writing Patient Assessments for Pediatric Clerkship Students
Source: MedEdPORTAL. 2020 Nov 9;16:11029. doi: 10.15766/mep_2374-8265.11029 (PMC7666838; doi:10.15766/mep_2374-8265.11029)
Supplement: Supplementary file 1 — PowerPoint Presentation.pptxInstructor Script.docxSample H&P 1.docxSample H&P 2.docxSample H&P 3.docxP-HAPEE Isolated Scoring Tool.docxAssessment Examples for Sample H&Ps.docxMedical Semantics Crossword.pdfCrossword Puzzle Answers.docx [file mep_2374-8265.11029-s001.zip › E. Sample H&P 3.docx]

**Sample H&P (3)**

**Formulate Your Own Assessment**

**Instructions: Read the following H&P. Synthesize the patient presentation and use clinical reasoning to formulate your own assessment and differential diagnosis. Arrows denote the direction of an abnormal value.**

**CC:** “fever”

**HPI**: 15-year-old girl who presents with fever. Per mom patient has had daily fever up to 103 F for the last 9 days. She states that the fever does go down when she gives her Tylenol or ibuprofen but then returns. The fever occurs at any time during the day. Mom also notes that the patient has been more tired lately and has not been able to get up and go to school because of her fatigue. She went on a high school trip with her band group in the last month. Per mom she was well during that trip. She states another child in the group recently developed Lyme disease. She is worried that her child may have that as well. Patient also started with some cough, runny nose, and sore throat one week ago close to the time the fevers started and still has these symptoms. Mom states that initially when fever started patient had a faint pink to red colored rash but that resolved after the first couple of days and she has not seen that again.

**ROS**:

General: + daily fever as per HPI, +weight loss mom thinks approx 5 pounds since she has been so tired and not eating

HENT: no history of ear infections, no head trauma, +sore throat, +cough,+ runny nose

Eyes: no eye drainage, no vision problems

GI: no diarrhea, no bloody stools

Cardiac: deny any history of murmurs

Pulmonary: no difficulty breathing

GU: +urine is darker in color, +urinating less than previously

Neuro: no delays, denies any seizures

Heme: no bruising noted but patient appears more pale than previously

Endo: normal newborn screens

Allergy: no known allergies

**PMH**:

Birth: 38 weeks gestation, no NICU stay, born vaginally, came home with mom

Other diagnoses and surgeries: history of left knee swelling (this occurred 4 times in the last year) that resolves with ibuprofen, mom thought likely due to injuries while playing soccer; broken elbow while playing on monkey bars at age 10

Social: attends 11th grade, competitive year round soccer player for the last 3 years, one dog at home HEADDS exam: patient states that she is sexually active and had unprotected sex this year, no drug or alcohol use, doing well in school

Vaccines: patient is NOT vaccinated (only received hepatitis B at birth); mom chose not to vaccinate her kids after her sister was diagnosed with Autism

Meds: none

Family History: younger sister diagnosed with leukemia 3 years ago; maternal aunt with Autism; mom with lupus

**Physical Exam**:

Vitals: T. **103 F ↑**, **HR 110 bpm ↑**, RR 20, BP 100/65, O_2_ sat 99% on room air

General: awake and interactive in no acute distress, pale appearance

HEENT: normocephalic, atraumatic; tacky mucous membranes, erythematous tonsils enlarged to 3+ with some exudates, TMs clear bilaterally, nasal turbinates not inflamed

Lymph: multiple enlarged palpable lymph nodes approx 2-3 cm in cervical region, axillary, and inguinal region

Respiratory: clear to auscultation throughout, no nasal flaring, no retractions or belly breathing

Cardiac: tachycardic, normal S1 and S2, II/VI systolic ejection murmur appreciated, capillary refill approximately 4 seconds

GI: soft, flat, tenderness to palpation in left abdomen, +palpable spleen in left lower quadrant, and patient seems to wince when you palpate her right upper quadrant

GU: Tanner Stage 4 female, no fissures or skin tags on perianal exam, no genital lesions

Neuro: strength 5/5 in arms, 5/5 in legs, deep tendon reflexes 2+ , cranial nerves intact

Skin: no rashes, no birthmarks, no nevi, no bruises

**Labs/Imaging**:

Na 137, K 4.0, Cl 103, Bicarb 18, BUN 25, Cr 0.9 ,Gluc 90, Ca 9.0, Albumin 3.0

WBC: **3,000↓,** diff showing **26% neutrophils ↓**, **70% atypical lymphocytes↑**, 4% monophils,

Hemoglobin **9.0↓** MCV 89 (nl), Platelets **100,000↓**

**Monospot positive**

Abdominal Ultrasound: **enlarged liver and spleen**
