# Supplementary material for: Skill Session on Writing Patient Assessments for Pediatric Clerkship Students
Source: MedEdPORTAL. 2020 Nov 9;16:11029. doi: 10.15766/mep_2374-8265.11029 (PMC7666838; doi:10.15766/mep_2374-8265.11029)
Supplement: Supplementary file 1 — PowerPoint Presentation.pptxInstructor Script.docxSample H&P 1.docxSample H&P 2.docxSample H&P 3.docxP-HAPEE Isolated Scoring Tool.docxAssessment Examples for Sample H&Ps.docxMedical Semantics Crossword.pdfCrossword Puzzle Answers.docx [file mep_2374-8265.11029-s001.zip › F. P-HAPEE Isolated Scoring Tool.docx]

**Appendix F**

Adapted from the Pediatric History and Physical Exam Evaluation (P-HAPEE)^†^ rubric that graders were trained to use when scoring students’ pre and post skill session written patient assessment and differential diagnosis.

***Assessment***

| 1 | 2 | 3 | 4 | 5 |
| --- | --- | --- | --- | --- |
| Either no assessment given or assessment is missing essential components like physical exam or diagnostic study findings. Restates all relevant and irrelevant subjective and objective data without synthesizing or extracting only pertinent information. |  | Assessment has all the essential components of the subjective and objective patient data but lacks synthesis and interpretation using medical terms. Some use of precise medical terms and semantic qualifiers further characterizing findings. |  | Assessment has all the essential components from the subjective and objective portions of the patient data. Uses synthesizing, interpretative medical terminology. Uses appropriate precise semantic qualifiers to depict the clinical presentation concisely. |

***Differential Diagnosis***

| 1 | 2 | 3 | 4 | 5 |
| --- | --- | --- | --- | --- |
| May not state or commit to a diagnosis, if diagnosis is stated it is not supported with appropriate patient information. |  | Commits to a working diagnosis and includes several plausible differential diagnoses. Demonstrates clinical reasoning utilizing appropriate subjective and objective patient data. |  | Commits to an accurate working diagnosis, compares and contrasts appropriate differential diagnoses utilizing clinical reasoning with relevant supporting or refuting information. References literature when appropriate. |

^†^King MA, Phillipi CA, Buchanan PM, Lewin LO. Developing validity evidence for the written pediatric history and physical exam evaluation (P-HAPEE) rubric. Academic pediatrics. 2017 Jan 1;17(1):68-73.
