# Supplementary material for: Skill Session on Writing Patient Assessments for Pediatric Clerkship Students
Source: MedEdPORTAL. 2020 Nov 9;16:11029. doi: 10.15766/mep_2374-8265.11029 (PMC7666838; doi:10.15766/mep_2374-8265.11029)
Supplement: Supplementary file 1 — PowerPoint Presentation.pptxInstructor Script.docxSample H&P 1.docxSample H&P 2.docxSample H&P 3.docxP-HAPEE Isolated Scoring Tool.docxAssessment Examples for Sample H&Ps.docxMedical Semantics Crossword.pdfCrossword Puzzle Answers.docx [file mep_2374-8265.11029-s001.zip › G. Assessment Examples for Sample H&Ps.docx]

**Appendix F: Assessment Examples for Sample H&Ps**

**Sample H&P 1**

*Assessment*

7-year-old full term girl with PMHx significant for **single prior UTI** and **RSV bronchiolitis** hospitalization in infancy, **chronic intermittent abdominal pain attributed to chronic constipation** presenting initially with **gastroenteritis** symptoms causing **moderate dehydration** after exposure to foods at a picnic and a petting zoo, now presenting with **acute hematochezia in setting of enteritis** as well as **diffuse abdominal pain**. Labs concerning for **acute kidney injury** complicated by **azotemia, hyperkalemia, metabolic acidosis** in setting of **hematuria**, **hemolysis as evidence by schistocytes**, **normocytic anemia** and **thrombocytopenia** most consistent with a diagnosis of **hemolytic uremic syndrome secondary to bacterial gastroenteritis** as evidenced by the **neutrophilic leukocytosis** and **bloody enteritis.**

*Differential Diagnosis*

Other diagnoses to consider are nephrolithiasis as explained by kidney injury and hematuria but would not explain the gastroenteritis symptoms. One could also consider IBD especially in setting of positive family history however this would likely be associated with chronic symptoms and weight loss instead of acute symptoms that started in the last few days.

**Sample H&P 2**

*Assessment*

15-year-old full term boy with PMHx significant for **milk protein allergy in infancy** presenting with **hematochezia** in setting of **chronic diarrhea** causing significant **tenesmus** and **weight loss,** found to be **moderately dehydrated** and have **diffuse abdominal tenderness** worse in **the left quadrant** as well as a **fissure** on perianal exam with labs concerning for **significantly elevated inflammatory markers**, **normocytic anemia** and **protein calorie malnutrition** supported by **hypoalbuminemia**. The presentation is most consistent with a diagnosis of inflammatory bowel disease. The metabolic acidosis may be explained by the chronic diarrhea and loss of bicarb in the stool. Calculating anion gap could help further differentiate among other differential diagnoses.

*Differential Diagnosis*

One could also consider gastroenteritis but significant weight loss and chronic diarrhea in setting of anemia and elevated inflammatory markers is more consistent with a chronic etiology rather than an acute etiology. Due to presence of hyperglycemia and acidosis should also consider diabetic ketoacidosis however would expect random blood glucose to be greater than 200 and this diagnosis is generally not consistent with elevated inflammatory markers. Presentation could also be consistent with HIV especially in setting of significant diarrhea, weight loss and anemia and high risk behaviors including multiple sexual partners and marijuana and heroin.

**Sample H&P 3**

*Assessment*

15-year-old full term **unvaccinated** girl with PMHx significant for **chronic intermittent left knee arthralgia** presenting with **prolonged** fever and fatigue **preceded** by **URI** illness and brief resolved rash. Exam concerning for **moderate dehydration**, **diffuse lymphadenopathy**, **palpable splenomegaly**, and **enlarged exudative tonsils** with diagnostic workup revealing **pancytopenia** with significant **atypical lymphocytosis** and **hepatosplenomegaly**. Clinical picture is most consistent with diagnosis of acute infectious mononucleosis secondary to EBV.

*Differential Diagnosis*

Other diagnoses to consider would be malignancy like leukemia especially because of pancytopenia and prolonged fevers, however would have possibly expected more significant weight loss or blasts on peripheral smear; could rule out by doing a peripheral smear. Another diagnosis to consider would be an autoimmune condition like JIA (juvenile idiopathic arthritis) especially because patient has a history of chronic intermittent knee swelling, however this diagnosis is not supported by the lymphadenopathy and hepatosplenomegaly. Another autoimmune condition to consider would be lupus that can present with anemia, thrombocytopenia and prolonged fevers which is also supported by patient’s family history maternal lupus, however may not necessarily have enlarged exudative tonsils and diffuse lymphadenopathy. Also, in this age group patients with lupus may have organ involvement specifically the kidney so one could check for proteinuria or send C3 or C4 which may be decreased in addition to ANA or dsDNA studies.
