# Supplementary material for: Skill Session on Writing Patient Assessments for Pediatric Clerkship Students
Source: MedEdPORTAL. 2020 Nov 9;16:11029. doi: 10.15766/mep_2374-8265.11029 (PMC7666838; doi:10.15766/mep_2374-8265.11029)
Supplement: Supplementary file 1 — PowerPoint Presentation.pptxInstructor Script.docxSample H&P 1.docxSample H&P 2.docxSample H&P 3.docxP-HAPEE Isolated Scoring Tool.docxAssessment Examples for Sample H&Ps.docxMedical Semantics Crossword.pdfCrossword Puzzle Answers.docx [file mep_2374-8265.11029-s001.zip › H. Medical Semantics Crossword.pdf]

MEDICAL SEMANTICS

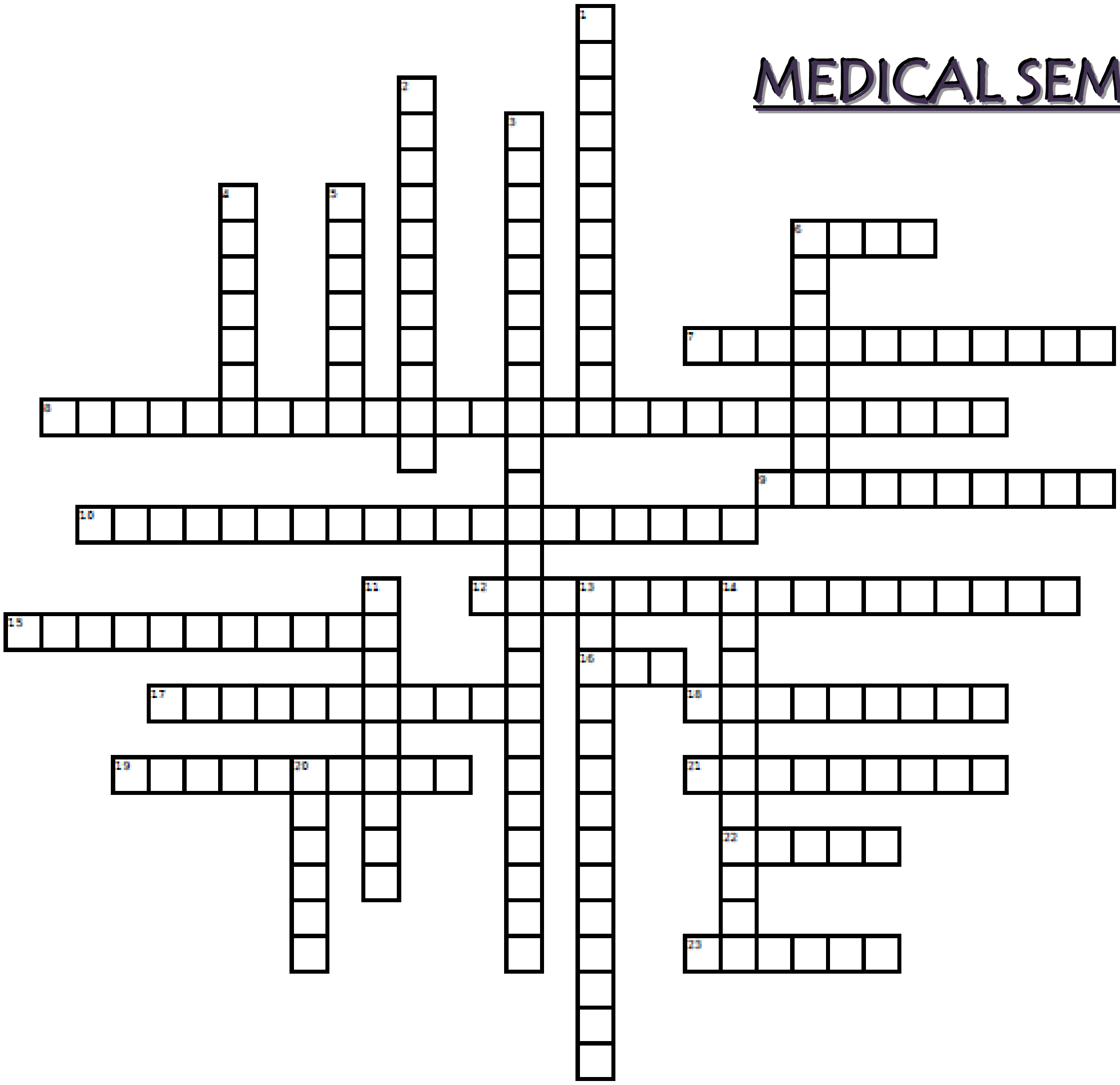

All of the following describe terms that are often used in assessment statements. These terms can either be medical terms/diagnoses that summarize clinical information or terms that are modifiers that further describe a condition.

Down

1. Term used for gross blood in the stools

2. Involving much or all of the body
3. Elevated white blood cells that are predominantly neutrophils (2 words)
4. Spread everywhere in an area
5. Condition that has persisted for a long period of time
6. Average in quality or degree

11. Characterized by black pigment for example in stool or moles
13. Condition that has been present for a long time that has now worsened over a short period of time (3 words)
14. Inability to tolerate oral intake can be phrased as ‘feeding ___’
20. Persistent presentation requiring multiple interventions to 'break' or the position of affairs

Across

6. Term that describes the least spicy salsa or a condition that is concerning but not grave
7. Occurring at irregular intervals, not continuous
8. Significant work of breathing requiring oxygen support (3 words)
9. Continuous symptoms
10. pH less than 7.3 with CO2 above 55 (2 words)
12. pH less than 7.3 with bicarbonate less than 18 (2 words)
15. Increasing in severity or extent
16. Abbreviation for cold symptoms

17. As a result of (2 words)
18. Restricted to a particular area

19. term used for something that has already occurred often used after surgeries (2 words)
21. Symptoms that have occurred before and resolved now occu again
22. Term used to describe of sudden onset or short duration
23. Term describing something intense or grave
