# Supplementary figures and images for: Skill Session on Writing Patient Assessments for Pediatric Clerkship Students
Source: MedEdPORTAL. 2020 Nov 9;16:11029. doi: 10.15766/mep_2374-8265.11029 (PMC7666838; doi:10.15766/mep_2374-8265.11029)

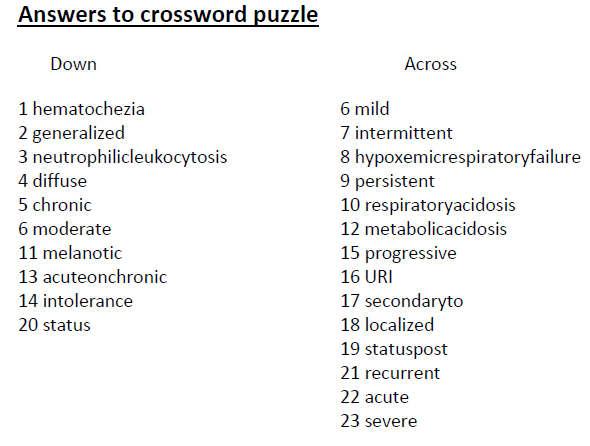


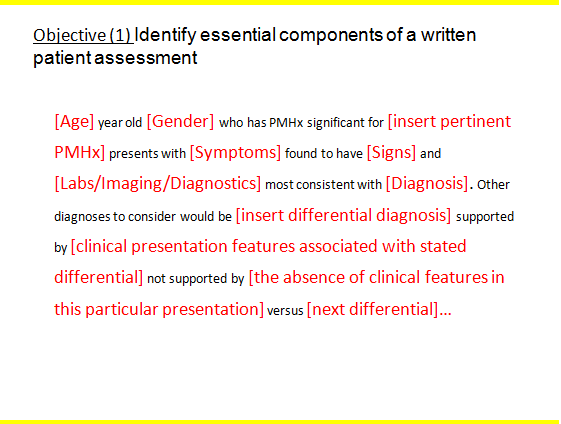

Supplement: Supplementary file 1 — PowerPoint Presentation.pptxInstructor Script.docxSample H&P 1.docxSample H&P 2.docxSample H&P 3.docxP-HAPEE Isolated Scoring Tool.docxAssessment Examples for Sample H&Ps.docxMedical Semantics Crossword.pdfCrossword Puzzle Answers.docx [file mep_2374-8265.11029-s001.zip › I. Crossword Puzzle Answers.docx]
